# Supplementary material for: Diagnostic accuracy of triglyceride to glucose index and triglyceride/high-density lipoprotein index for insulin resistance among children and adolescents: A systematic review
Source: PLoS One. 2025 Jun 25;20(6):e0326179. doi: 10.1371/journal.pone.0326179 (PMC12192287; doi:10.1371/journal.pone.0326179)
Supplement: S2 Table — Complete search strategy used for each database. (DOCX) [file pone.0326179.s002.docx]

**S2 Table. Search strategy**

| Database | Search strategy |
| --- | --- |
| PubMed | #1: (“Triglyceride and high-density lipoprot*”[TIAB] OR “triglyceride to high-density lipoprot*”[TIAB] OR “triglyceride-to-HDL”[TIAB] OR “Triglyceride to HDL”[TIAB] OR “TG/HDL”[TIAB] OR “TG:HDL”[TIAB] OR “Triglyceride-to-High-density Lipoprot*”[TIAB] OR “TGHDL”[TIAB] OR “triglyceride/HDL”[TIAB] OR “Triglyceride/High-Density Lipoprot*”[TIAB] OR “Triglyceride and hdl”[TIAB] OR “Triglycerides and high-density lipoprot*”[TIAB] OR “triglycerides to high-density lipoprot*”[TIAB] OR “triglycerides-to-HDL”[TIAB] OR “Triglycerides to HDL”[TIAB] OR “Triglycerides-to-High-density Lipoprot*”[TIAB] OR “triglycerides/HDL”[TIAB] OR “Triglycerides/High-Density Lipoprot*”[TIAB] OR “Triglycerides and hdl”[TIAB])  #2: (“Triglyceride and glucos*”[TIAB] OR “triglyceride to glucos*”[TIAB] OR “triglyceride-toglucos*”[TIAB] OR “Triglyceride to glucos*”[TIAB] OR “Triglyceride-glucos*”[TIAB] OR “Triglyceride/glucos*”[TIAB] OR tyg[tiab] OR “TriGlycerides and glucos*”[TIAB] OR “triglycerides to glucos*”[TIAB] OR “triglycerides-to-glucos*”[TIAB] OR “Triglycerides to glucos*”[TIAB] OR “Triglycerides-glucos*”[TIAB] OR “Triglycerides/glucos*”[TIAB])  #3: (Insulin Resistance[MH] OR “Insulin Resist*”[tiab] OR “Insulin Sensitiv*”[tiab])  #4: (Adolescent[MH] OR Adolescen*[tiab] OR Teen*[tiab] OR Youth*[tiab] OR Child[MH] OR Child*[tiab])  #5: (#1 OR #2) AND #3 AND #4 |
| Scopus | #1: (TITLE-ABS-KEY(“Triglyceride and high-density lipoprot*” OR “triglyceride to high-density lipoprot*” OR “triglyceride-to-HDL” OR “Triglyceride to HDL” OR “TG/HDL” OR “TG:HDL” OR “Triglyceride-to-High-density Lipoprot*” OR “TG-HDL” OR “triglyceride/HDL” OR “Triglyceride/High-Density Lipoprot*” OR “Triglyceride and hdl” OR “Triglycerides and highdensity lipoprot*” OR “triglycerides to high-density lipoprot*” OR “triglycerides-to-HDL” OR “Triglycerides to HDL” OR “Triglycerides-to-High-density Lipoprot*” OR “triglycerides/HDL” OR “Triglycerides/High-Density Lipoprot*” OR “Triglycerides and hdl”)  #2: TITLE-ABSKEY(“Triglyceride and glucos*” OR “triglyceride to glucos*” OR “triglyceride-to-glucos*” OR “Triglyceride to glucos*” OR “Triglyceride-glucos*” OR “Triglyceride/glucos*” OR tyg OR “TriGlycerides and glucos*” OR “triglycerides to glucos*” OR “triglycerides-to-glucos*” OR “Triglycerides to glucos*” OR “Triglycerides-glucos*” OR “Triglycerides/glucos*”)  #3: TITLEABS-KEY(“Insulin Resist*” OR “Insulin Sensitiv*”)  #4: TITLE-ABS-KEY(Adolescen* OR Teen* OR Youth* OR Child*)  #5: (#1 OR #2) AND #3 AND #4 |
| WOS | #1: TS=(“Triglyceride and high-density lipoprot*” OR “triglyceride to high-density lipoprot*” OR “triglyceride-to-HDL” OR “Triglyceride to HDL” OR “TG/HDL” OR “TG:HDL” OR “Triglyceride-toHigh-density Lipoprot*” OR “TG-HDL” OR “triglyceride/HDL” OR “Triglyceride/High-Density Lipoprot*” OR “Triglyceride and hdl” OR “Triglycerides and high-density lipoprot*” OR “triglycerides to high-density lipoprot*” OR “triglycerides-to-HDL” OR “Triglycerides to HDL” OR “Triglycerides-to-High-density Lipoprot*” OR “triglycerides/HDL” OR “Triglycerides/HighDensity Lipoprot*” OR “Triglycerides and hdl”) OR TI=(“Triglyceride and high-density lipoprot*” OR “triglyceride to high-density lipoprot*” OR “triglyceride-to-HDL” OR “Triglyceride to HDL” OR “TG/HDL” OR “TG:HDL” OR “Triglyceride-to-High-density Lipoprot*” OR “TG-HDL” OR “triglyceride/HDL” OR “Triglyceride/High-Density Lipoprot*” OR “Triglyceride and hdl” OR “Triglycerides and high-density lipoprot*” OR “triglycerides to high-density lipoprot*” OR “triglycerides-to-HDL” OR “Triglycerides to HDL” OR “Triglycerides-to-High-density Lipoprot*” OR “triglycerides/HDL” OR “Triglycerides/High-Density Lipoprot*” OR “Triglycerides and hdl”) OR AK=(“Triglyceride and high-density lipoprot*” OR “triglyceride to high-density lipoprot*” OR “triglyceride-to-HDL” OR “Triglyceride to HDL” OR “TG/HDL” OR “TG:HDL” OR “Triglyceride-toHigh-density Lipoprot*” OR “TG-HDL” OR “triglyceride/HDL” OR “Triglyceride/High-Density Lipoprot*” OR “Triglyceride and hdl” OR “Triglycerides and high-density lipoprot*” OR “triglycerides to high-density lipoprot*” OR “triglycerides-to-HDL” OR “Triglycerides to HDL” OR “Triglycerides-to-High-density Lipoprot*” OR “triglycerides/HDL” OR “Triglycerides/HighDensity Lipoprot*” OR “Triglycerides and hdl”) OR AB=(“Triglyceride and high-density lipoprot*” OR “triglyceride to high-density lipoprot*” OR “triglyceride-to-HDL” OR “Triglyceride to HDL” OR “TG/HDL” OR “TG:HDL” OR “Triglyceride-to-High-density Lipoprot*” OR “TG-HDL” OR “triglyceride/HDL” OR “Triglyceride/High-Density Lipoprot*” OR “Triglyceride and hdl” OR “Triglycerides and high-density lipoprot*” OR “triglycerides to high-density lipoprot*” OR “triglycerides-to-HDL” OR “Triglycerides to HDL” OR “Triglycerides-to-High-density Lipoprot*” OR “triglycerides/HDL” OR “Triglycerides/High-Density Lipoprot*” OR “Triglycerides and hdl”) OR KP=(“Triglyceride and high-density lipoprot*” OR “triglyceride to high-density lipoprot*” OR “triglyceride-to-HDL” OR “Triglyceride to HDL” OR “TG/HDL” OR “TG:HDL” OR “Triglyceride-toHigh-density Lipoprot*” OR “TG-HDL” OR “triglyceride/HDL” OR “Triglyceride/High-Density Lipoprot*” OR “Triglyceride and hdl” OR “Triglycerides and high-density lipoprot*” OR “triglycerides to high-density lipoprot*” OR “triglycerides-to-HDL” OR “Triglycerides to HDL” OR “Triglycerides-to-High-density Lipoprot*” OR “triglycerides/HDL” OR “Triglycerides/HighDensity Lipoprot*” OR “Triglycerides and hdl”)  #2: TS=(“Triglyceride and glucos*” OR “triglyceride to glucos*” OR “triglyceride-to-glucos*” OR “Triglyceride to glucos*” OR “Triglyceride-glucos*” OR “Triglyceride/glucos*” OR tyg OR “TriGlycerides and glucos*” OR “triglycerides to glucos*” OR “triglycerides-to-glucos*” OR “Triglycerides to glucos*” OR “Triglycerides-glucos*” OR “Triglycerides/glucos*”) OR TI=(“Triglyceride and glucos*” OR “triglyceride to glucos*” OR “triglyceride-to-glucos*” OR “Triglyceride to glucos*” OR “Triglyceride-glucos*” OR “Triglyceride/glucos*” OR tyg OR “TriGlycerides and glucos*” OR “triglycerides to glucos*” OR “triglycerides-to-glucos*” OR “Triglycerides to glucos*” OR “Triglycerides-glucos*” OR “Triglycerides/glucos*”) OR AB=(“Triglyceride and glucos*” OR “triglyceride to glucos*” OR “triglyceride-to-glucos*” OR “Triglyceride to glucos*” OR “Triglyceride-glucos*” OR “Triglyceride/glucos*” OR tyg OR “TriGlycerides and glucos*” OR “triglycerides to glucos*” OR “triglycerides-to-glucos*” OR “Triglycerides to glucos*” OR “Triglycerides-glucos*” OR “Triglycerides/glucos*”) OR AK=(“Triglyceride and glucos*” OR “triglyceride to glucos*” OR “triglyceride-to-glucos*” OR “Triglyceride to glucos*” OR “Triglyceride-glucos*” OR “Triglyceride/glucos*” OR tyg OR “TriGlycerides and glucos*” OR “triglycerides to glucos*” OR “triglycerides-to-glucos*” OR “Triglycerides to glucos*” OR “Triglycerides-glucos*” OR “Triglycerides/glucos*”) OR KP=(“Triglyceride and glucos*” OR “triglyceride to glucos*” OR “triglyceride-to-glucos*” OR “Triglyceride to glucos*” OR “Triglyceride-glucos*” OR “Triglyceride/glucos*” OR tyg OR “TriGlycerides and glucos*” OR “triglycerides to glucos*” OR “triglycerides-to-glucos*” OR “Triglycerides to glucos*” OR “Triglycerides-glucos*” OR “Triglycerides/glucos*”)  #3: TS=(“Insulin Resist*” OR “Insulin Sensitiv*”) OR TI=(“Insulin Resist*” OR “Insulin Sensitiv*”) OR AB=(“Insulin Resist*” OR “Insulin Sensitiv*”) OR AK=(“Insulin Resist*” OR “Insulin Sensitiv*”) OR KP=(“Insulin Resist*” OR “Insulin Sensitiv*”)  #4: TS=(Adolescen* OR Teen* OR Youth* OR Child*) OR TI=(Adolescen* OR Teen* OR Youth* OR Child*) OR AB=(Adolescen* OR Teen* OR Youth* OR Child*) OR AK=(Adolescen* OR Teen* OR Youth* OR Child*) OR KP=(Adolescen* OR Teen* OR Youth* OR Child*)  #5: (#1 OR #2) AND #3 AND #4 |
| Embase | #1: (‘Triglyceride and high-density lipoprot*’ OR ‘triglyceride to high-density lipoprot*’ OR ‘triglyceride-to-HDL’ OR ‘Triglyceride to HDL’ OR ‘TG/HDL’ OR ‘TG:HDL’ OR ‘Triglyceride-toHigh-density Lipoprot*’ OR ‘TG-HDL’ OR ‘triglyceride/HDL’ OR ‘Triglyceride/High-Density Lipoprot*’ OR ‘Triglyceride and hdl’ OR ‘Triglycerides and high-density lipoprot*’ OR ‘triglycerides to high-density lipoprot*’ OR ‘triglycerides-to-HDL’ OR ‘Triglycerides to HDL’ OR ‘Triglycerides-to-High-density Lipoprot*’ OR ‘triglycerides/HDL’ OR ‘Triglycerides/High-Density Lipoprot*’ OR ‘Triglycerides and hdl’)  #2: (‘Triglyceride and glucos*’ OR ‘triglyceride to glucos*’ OR ‘triglyceride-to-glucos*’ OR ‘Triglyceride to glucos*’ OR ‘Triglyceride-glucos*’ OR ‘Triglyceride/glucos*’ OR tyg OR ‘TriGlycerides and glucos*’ OR ‘triglycerides to glucos*’ OR ‘triglycerides-to-glucos*’ OR ‘Triglycerides to glucos*’ OR ‘Triglycerides-glucos*’ OR ‘Triglycerides/glucos*’)  #3: ('insulin resistance'/exp OR 'insulin resistan*' OR 'resistance, insuline' OR 'Insulin Sensitiv*')  #4: ('child'/exp OR 'child*' OR 'adolescent'/exp OR 'adolescen*' OR 'teen*' OR 'adolescence'/exp OR 'adolescence' OR 'teenage' OR 'youth*')  #5: (#1 OR #2) AND #3 AND #4 |
