# Supplementary material for: Diagnostic accuracy of triglyceride to glucose index and triglyceride/high-density lipoprotein index for insulin resistance among children and adolescents: A systematic review
Source: PLoS One. 2025 Jun 25;20(6):e0326179. doi: 10.1371/journal.pone.0326179 (PMC12192287; doi:10.1371/journal.pone.0326179)
Supplement: S3 Table — Adaptation created by authors to assess certainty of evidence in narrative syntheses. (DOCX) [file pone.0326179.s003.docx]

**S3 Table. Decision table based on GRADE system (Adaptation by authors).**

| **Start with** | **Criteria** | **Risk of bias** | **Inconsistency** | **Indirectness** | **Imprecision** | **Publication bias** | |
| --- | --- | --- | --- | --- | --- | --- | --- |
| High confidence:  Studies included were cross-sectional with the main objective of measuring prevalence | Not serious | <30% of the evaluated population have high risk of bias | No heterogeneity noted | The inclusion criteria correspond to the population | 95% CI range (upper limit minus lower limit) is less than 10% | No estimated or methodological publication bias | |
| Low confidence: Included cross-sectional studies without the main objective of measuring prevalence | Serious | >30% and <50% of the evaluated population included have high risk of bias | Heterogeneity among studies | The inclusion criteria do not correspond to the population | The 95% CI range is between 10% and 20% | Analyze if the search was exhaustive |  |
|  | Very serious | >50% of the evaluated population have a high risk of bias |  |  | The 95% CI range is greater than 20% |  |  |
