# Supplementary material for: Diagnostic accuracy of triglyceride to glucose index and triglyceride/high-density lipoprotein index for insulin resistance among children and adolescents: A systematic review
Source: PLoS One. 2025 Jun 25;20(6):e0326179. doi: 10.1371/journal.pone.0326179 (PMC12192287; doi:10.1371/journal.pone.0326179)
Supplement: S4 Table — Includes all studies reviewed for inclusion, with reasons for exclusion when applicable. (DOCX) [file pone.0326179.s004.docx]

**S4 Table. Studies evaluated in the selection process.**

| **N°** | **Title** | **Year** | **Journal** | **Reason for exclusion** |
| --- | --- | --- | --- | --- |
| 1 | Serum magnesium level and its relationship with insulin resistance in obese children | 2010 | Guncel pediatri | This study was excluded because it did not provide outcomes on the diagnostic performance of Tg/glucose or Tg/HDL index. |
| 2 | Metabolic Alterations During Valproic Acid Treatment: A Prospective Study | 2009 | Pediatric neurology | This study was excluded because it did not provide outcomes on the diagnostic performance of Tg/glucose or Tg/HDL index. |
| 3 | Relationships between plasma lipoprotein concentrations and insulin action in an obese hyperinsulinemic population | 1987 | Diabetes | This study was excluded because it did not provide outcomes on the diagnostic performance of Tg/glucose or Tg/HDL index. |
| 4 | The effect of concentrated pomegranate juice consumption on risk factors of cardiovascular diseases in women with polycystic ovary syndrome: A randomized controlled trial | 2021 | Phytotherapy research | This study was excluded because it did not provide outcomes on the diagnostic performance of Tg/glucose or Tg/HDL index. |
| 5 | Lipid Accumulation Product (LAP) and Visceral Adiposity Index (VAI) as Markers of Insulin Resistance and Metabolic Associated Disturbances in Young Argentine Women with Polycystic Ovary Syndrome | 2017 | Hormone and metabolic research | This study was excluded because it did not provide outcomes on the diagnostic performance of Tg/glucose or Tg/HDL index. |
| 6 | Omentin-1: Novel Target in Childhood Obesity | 2016 | Research journal of pharmaceutical biological and chemical sciences | This study was excluded because it did not provide outcomes on the diagnostic performance of Tg/glucose or Tg/HDL index. |
| 7 | Correlations of advanced glycation end products and their receptors with adiposity and adiposity-related co-morbidity risk are different in children than in adults | 2012 | Diabetes | This study was excluded because it did not provide outcomes on the diagnostic performance of Tg/glucose or Tg/HDL index. |
| 8 | A study Of sdLDL-C and insulin resistance in apparently healthy obese young adults of southern part of indian subcontinent | 2015 | Clinical chemistry | This study was excluded because it did not provide outcomes on the diagnostic performance of Tg/glucose or Tg/HDL index. |
| 9 | Cardiovascular risk factors, nutritional status on HOMA-IR, QUICKI and TG/c-HDL indexes in pubertal adolescents | 2013 | Acta bioquimica clinica latinoamericana | This study was excluded because it did not provide outcomes on the diagnostic performance of Tg/glucose or Tg/HDL index. |
| 10 | Relationships of serum leptin to clinical and anthropometric findings in obese patients | 2002 | Obesity surgery | This study was excluded because it did not provide the exposure on Tg/glucose or Tg/HDL index values. |
| 11 | Maternal lipid profile in early pregnancy is associated with foetal growth and the risk of a child born large-for-gestational age: a population-based prospective cohort study Maternal lipid profile in early pregnancy and foetal growth | 2020 | Bmc medicine | This study was excluded because it did not provide the exposure on Tg/glucose or Tg/HDL index values. |
| 12 | Linking definition of childhood and adolescent obesity to current health outcomes | 2010 | Int j pediatr obes | This study was excluded because it did not provide the exposure on Tg/glucose or Tg/HDL index values. |
| 13 | Adiponectin status in different metabolic phenotypes on insulin resistance | 2018 | Annals of Nutrition and Metabolism | This study was excluded because it did not provide the exposure on Tg/glucose or Tg/HDL index values. |
| 14 | Association between Leptin, Adiponectin, and Leptin/Adiponectin Ratio with Clustered Metabolic Risk Factors in Portuguese Adolescents: The LabMed Physical Activity Study | 2017 | Annals of Nutrition and Metabolism | This study was excluded because it did not provide the exposure on Tg/glucose or Tg/HDL index values. |
| 15 | Triglycerides/High density lipoprotein cholesterol ratio as a cardiometabolic risk marker in children and adolescents from Merida city, Venezuela | 2018 | Endocrinologia diabetes y nutricion | This study was excluded because it did not provide outcomes on the diagnostic performance of Tg/glucose or Tg/HDL index. |
| 16 | Triglycerides/High density lipoprotein cholesterol ratio as a cardiometabolic risk marker in children and adolescents from Mérida city, Venezuela | 2018 | Endocrinologia, Diabetes y Nutricion | This study was excluded because it did not provide outcomes on the diagnostic performance of Tg/glucose or Tg/HDL index. |
| 17 | Metabolic syndrome in Iranian youths: a population-based study on junior and high schools students in rural and urban areas | 2013 | J diabetes res | This study was excluded because it did not provide the exposure on Tg/glucose or Tg/HDL index values. |
| 18 | Long-term prednisone versus hydrocortisone treatment in children with classic congenital adrenal hyperplasia (CAH) and a brief review of the literature | 2019 | Acta biomedica | This study was excluded because it did not provide outcomes on the diagnostic performance of Tg/glucose or Tg/HDL index. |
| 19 | Association of Muscular Endurance and Cardiopulmonary Fitness to Insulin Resistance and the Risk Factors of CVD Among Male High School Students | 2010 | The Korean Society of Living Environmental System | This study was excluded because it did not provide outcomes on the diagnostic performance of Tg/glucose or Tg/HDL index. |
| 20 | Prevalence of obstructive sleep apnea among overweight adolescents and children in Korea: A single-center, retrospective study | 2019 | Sleep | This study was excluded because it did not provide outcomes on the diagnostic performance of Tg/glucose or Tg/HDL index. |
| 21 | Metabolic syndrome in young children: definitions and results of the IDEFICS study | 2014 | International journal of obesity | This study was excluded because it did not provide the exposure on Tg/glucose or Tg/HDL index values. |
| 22 | Lipoprotein(a), tissue plasminogen activator and plasminogen activator inhibitor 1 levels in hyperlipidaemic patients in Kuwait | 1997 | European journal of clinical investigation | This study was excluded because it did not provide the exposure on Tg/glucose or Tg/HDL index values. |
| 23 | Potential Association of Triglyceride Glucose Index with Cardiac Autonomic Neuropathy in Type 2 Diabetes Mellitus Patients | 2017 | Journal of Korean medical science | This study was excluded because it did not provide an adolescent or paediatric population. |
| 24 | Is there a relationship between cardiovascular risk factors and dehydroepiandrosterone sulfate levels in childhood obesity? | 2015 | Journal of Pediatric Endocrinology and Metabolism | This study was excluded because it did not provide the exposure on Tg/glucose or Tg/HDL index values. |
| 25 | The epidemic of obesity and metabolic syndrome among children and adolescents in the Emirates of Abu Dhabi: Hepatic abnormalities and comorbid health implications. A single tertiary centre experience | 2021 | Journal of Pediatric Gastroenterology and Nutrition | This study was excluded because it did not provide the exposure on Tg/glucose or Tg/HDL index values. |
| 26 | Genetic and metabolic determinants of increased plasma plasminogen activator inhibitor-1 activity in children with renal transplants | 2003 | Pediatric nephrology | This study was excluded because it did not provide the exposure on Tg/glucose or Tg/HDL index values. |
| 27 | Postprandial Effect of a High-Fat Meal on Endotoxemia in Arab Women with and without Insulin-Resistance-Related Diseases | 2015 | Nutrients | This study was excluded because it did not provide the exposure on Tg/glucose or Tg/HDL index values. |
| 28 | Parathyroid hormone is associated with biomarkers of chronic inflammation, independent of vitamin D status, in obese adolescents | 2011 | Endocrine reviews | This study was excluded because it did not provide the exposure on Tg/glucose or Tg/HDL index values. |
| 29 | Parathyroid hormone is associated with biomarkers of insulin resistance and inflammation, independent of vitamin D status, in obese adolescents | 2012 | Metabolic Syndrome and Related Disorders | This study was excluded because it did not provide the exposure on Tg/glucose or Tg/HDL index values. |
| 30 | Uric acidinduced low grade inflammation is mediated by parathyroid hormone: 25-hydroxyvitamin d ratio in obese adolescents | 2014 | Endocrine reviews | This study was excluded because it did not provide the exposure on Tg/glucose or Tg/HDL index values. |
| 31 | Gender differences in the association of insulin resistance and high-sensitivity c-reactive protein in obese adolescents | 2014 | Journal of Diabetes and Metabolic Disorders | This study was excluded because it did not provide the exposure on Tg/glucose or Tg/HDL index values. |
| 32 | Comparison of Apolipoprotein (ApoB/ApoA-1) and Lipoprotein (Total Cholesterol/HDL) Ratios in Obese Adolescents | 2018 | Metabolic Syndrome and Related Disorders | This study was excluded because it did not provide the exposure on Tg/glucose or Tg/HDL index values. |
| 33 | Comparison of apolipoprotein (ApoB/ApoA-1) and Lipoprotein (Total Cholesterol/HDL) Ratios in Obese Adolescents | 2017 | Diabetes | This study was excluded because it did not provide the exposure on Tg/glucose or Tg/HDL index values. |
| 34 | Cardiometabolic risk among Saudi children and adolescents: Saudi childrens overweight, obesity, and lifestyles (S.Ch.O.O.Ls) study | 2014 | Ann saudi med | This study was excluded because it did not provide the exposure on Tg/glucose or Tg/HDL index values. |
| 35 | Insulinaemia and insulin resistance in Caucasian general paediatric population aged 2 to 10 years: Associated risk factors | 2018 | Pediatric diabetes | This study was excluded because it did not provide the exposure on Tg/glucose or Tg/HDL index values. |
| 36 | The correct formula to calculate triglyceride-glucose index (TyG) | 2020 | Journal of pediatric endocrinology & metabolism | This study was excluded because it did not provide outcomes on the diagnostic performance of Tg/glucose or Tg/HDL index. |
| 37 | Pre-hypertension and hypertension in college students in Kuwait: a neglected issue | 2012 | Journal of family & community medicine | This study was excluded because it did not provide outcomes on the diagnostic performance of Tg/glucose or Tg/HDL index. |
| 38 | Circulating microRNAs and adipokines as markers of metabolic syndrome in adolescents with obesity | 2019 | Clin nutr | This study was excluded because it did not provide outcomes on the diagnostic performance of Tg/glucose or Tg/HDL index. |
| 39 | Obesity indices and major components of metabolic syndrome in young adult Arab subjects | 2004 | Annals of nutrition and metabolism | This study was excluded because it did not provide outcomes on the diagnostic performance of Tg/glucose or Tg/HDL index. |
| 40 | When traditional measures are not sufficient to manage the severe metabolic disturbances in patients with lipodystrophy syndromes, what's next? | 2017 | Endocrine reviews | This study was excluded because it did not provide outcomes on the diagnostic performance of Tg/glucose or Tg/HDL index. |
| 41 | Vitamin D3 deficiency is associated with more severe insulin resistance and metformin use in patients with type 2 diabetes | 2020 | Minerva endocrinologica | This study was excluded because it did not provide outcomes on the diagnostic performance of Tg/glucose or Tg/HDL index. |
| 42 | Waist-to-height ratio is as reliable as biochemical markers to discriminate pediatric insulin resistance | 2019 | J pediatr | Included |
| 43 | Lipodystrophy syndrome in human immunodeficiency virus-infected children | 2002 | Pediatric infectious disease journal | This study was excluded because it did not provide outcomes on the diagnostic performance of Tg/glucose or Tg/HDL index. |
| 44 | Metabolic syndrome in youths | 2007 | Pediatric diabetes | This study was excluded because it did not provide outcomes on the diagnostic performance of Tg/glucose or Tg/HDL index. |
| 45 | How does treating sleep-disordered breathing affect the lipid profile in obese children and adolescents? | 2012 | Endocrine reviews | This study was excluded because it did not provide outcomes on the diagnostic performance of Tg/glucose or Tg/HDL index. |
| 46 | Epistatic interactions of CDKN2B-TCF7L2 for risk of type 2 diabetes and of CDKN2B-JAZF1 for triglyceride/high-density lipoprotein ratio longitudinal change: evidence from the Framingham Heart Study | 2009 | BMC proceedings | This study was excluded because it did not provide outcomes on the diagnostic performance of Tg/glucose or Tg/HDL index. |
| 47 | Validity of triglyceride-glucose index as an indicator for metabolic syndrome in children and adolescents: the CASPIAN-V study | 2018 | Eating and weight disorders-studies on anorexia bulimia and obesity | This study was excluded because it did not provide outcomes on the diagnostic performance of Tg/glucose or Tg/HDL index. |
| 48 | Are non-high-density lipoprotein fractions associated with pediatric metabolic syndrome? The CASPIAN-V study | 2018 | Lipids health dis | This study was excluded because it did not provide outcomes on the diagnostic performance of Tg/glucose or Tg/HDL index. |
| 49 | Study of Insulin Resistance in Patients with β Thalassemia Major and Validity of Triglyceride Glucose (TYG) Index | 2018 | Journal of Pediatric Hematology/Oncology | This study was excluded because it did not provide outcomes on the diagnostic performance of Tg/glucose or Tg/HDL index. |
| 50 | Does Cardiorespiratory Fitness Modify the Association between Birth Weight and Insulin Resistance in Adult Life? | 2013 | Plos ONE | This study was excluded because it did not provide outcomes on the diagnostic performance of Tg/glucose or Tg/HDL index. |
| 51 | Indices of Central and Peripheral Obesity | 2015 | Balkan med j | This study was excluded because it did not provide outcomes on the diagnostic performance of Tg/glucose or Tg/HDL index. |
| 52 | Reduced insulin sensitivity and the presence of cardiovascular risk factors in short prepubertal children born small for gestational age (SGA) | 2005 | Clinical endocrinology | This study was excluded because it did not provide outcomes on the diagnostic performance of Tg/glucose or Tg/HDL index. |
| 53 | The rs822396 Polymorphism of the ADIPOQ Gene Is Associated with Anthropometric, Clinical, and Biochemical Alterations Related to the Metabolic Syndrome in the Mexican Population | 2020 | Metabolic syndrome and related disorders | This study was excluded because it did not provide outcomes on the diagnostic performance of Tg/glucose or Tg/HDL index. |
| 54 | Physical fitness, cardiometabolic risk and heart rate recovery in Chilean children | 2018 | Nutricion hospitalaria | This study was excluded because it did not provide outcomes on the diagnostic performance of Tg/glucose or Tg/HDL index. |
| 55 | Metabolic risk factors in young adults infected with HIV since childhood compared with the general population | 2018 | Plos ONE | This study was excluded because it did not provide outcomes on the diagnostic performance of Tg/glucose or Tg/HDL index. |
| 56 | Triglycerides/high-density lipoprotein cholesterol is a predictor similar to the triglyceride-glucose index for the diagnosis of metabolic syndrome using International Diabetes Federation criteria of insulin resistance in obese adolescents: A cross-sectional study | 2020 | Journal of Pediatric Endocrinology and Metabolism | This study was excluded because it did not provide outcomes on the diagnostic performance of Tg/glucose or Tg/HDL index. |
| 57 | Periapical Lesions Decrease Insulin Signaling in Rat Skeletal Muscle | 2015 | Journal of endodontics | This study was excluded because it did not provide outcomes on the diagnostic performance of Tg/glucose or Tg/HDL index. |
| 58 | The effect of ileal interposition in rats with metabolic syndrome | 2019 | Acta physiologica | This study was excluded because it did not provide outcomes on the diagnostic performance of Tg/glucose or Tg/HDL index. |
| 59 | Severe Obesity Is Associated With Impaired Arterial Smooth Muscle Function in Young Adults | 2011 | Obesity | This study was excluded because it did not provide an adolescent or paediatric population. |
| 60 | Polymorphisms in the C-reactive protein (CRP) gene are associated with non-alcoholic fatty liver disease (NAFLD) in Western Australian adolescents independent of insulin resistance | 2009 | Journal of Gastroenterology and Hepatology | This study was excluded because it did not provide the exposure on Tg/glucose or Tg/HDL index values. |
| 61 | Polymorphisms in adiponectin gene are associated with non-alcoholic fatty liver disease (NAFLD) in Western Australian adolescents | 2009 | Journal of Gastroenterology and Hepatology | This study was excluded because it did not provide the exposure on Tg/glucose or Tg/HDL index values. |
| 62 | Familial Aggregation of the Metabolic Syndrome: Tehran Lipid and Glucose Study | 2009 | Annals of nutrition and metabolism | This study was excluded because it did not provide the exposure on Tg/glucose or Tg/HDL index values. |
| 63 | Nonalcoholic fatty liver disease (NAFLD) outcome post-bariatric surgery in the teen-longitudinal assessment of bariatric surgery (teen-labs) consortium | 2018 | Diabetes | This study was excluded because it did not provide the exposure on Tg/glucose or Tg/HDL index values. |
| 64 | Targeting arginase-II enzyme with antagonist and the use of anti-inflammatory, antioxidants therapy in insulin resistance and heart diseases | 2013 | Journal of Diabetes | This study was excluded because it did not provide the exposure on Tg/glucose or Tg/HDL index values. |
| 65 | To study the efficacy of intervention strategies on features of metabolic syndrome in asian urban adolescent girls with pcos | 2012 | International Journal of Gynecology and Obstetrics | This study was excluded because it did not provide the exposure on Tg/glucose or Tg/HDL index values. |
| 66 | The prevalence of metabolic syndrome in obese adolescents of South Bronx: Comparison of IDF and NCEP criteria | 2011 | Endocrine reviews | This study was excluded because it did not provide the exposure on Tg/glucose or Tg/HDL index values. |
| 67 | Serum FGF21 Levels in Obese Korean Children and Adolescents | 2017 | Journal of obesity & metabolic syndrome | This study was excluded because it did not provide the exposure on Tg/glucose or Tg/HDL index values. |
| 68 | Associations between cardiorespiratory fitness, physical activity and clustered cardiometabolic risk in children and adolescents: the HAPPY study | 2012 | European journal of pediatrics | This study was excluded because it did not provide the exposure on Tg/glucose or Tg/HDL index values. |
| 69 | The triglyceride to high-density lipoprotein ratio identifies children who may be at risk of developing cardiometabolic disease | 2014 | Acta paediatr | This study was excluded because it did not provide the exposure on Tg/glucose or Tg/HDL index values. |
| 70 | Dyslipidemia in pregnant women and the age-rage axis in mothers and their neonates | 2017 | Endocrine practice | This study was excluded because it did not provide the exposure on Tg/glucose or Tg/HDL index values. |
| 71 | The Association Between Six Surrogate Insulin Resistance Indexes and Hypertension: A Population-Based Study | 2019 | Metabolic syndrome and related disorders | This study was excluded because it did not provide the exposure on Tg/glucose or Tg/HDL index values. |
| 72 | Sex specific differences of BCAA metabolism in childhood obesity and insulin resistance | 2020 | Hormone Research in Paediatrics | This study was excluded because it did not provide the exposure on Tg/glucose or Tg/HDL index values. |
| 73 | Insulin resistance in patients with post-adolescent acne | 2015 | International Journal of Dermatology | This study was excluded because it did not provide the exposure on Tg/glucose or Tg/HDL index values. |
| 74 | Assessment of Risk Factors for Metabolic Syndrome in Adolescents with Obesity in Ile-Ife, South West Nigeria | 2020 | West afr j med | This study was excluded because it did not provide the exposure on Tg/glucose or Tg/HDL index values. |
| 75 | Triglycerides/glucose index in obese children and adolescents | 2014 | Archives of Disease in Childhood | This study was excluded because it did not provide the exposure on Tg/glucose or Tg/HDL index values. |
| 76 | Metabolic syndrome in a 15-year-old girl with PCOS and acanthosis nigricans | 2007 | Diabetologia Doswiadczalna i Kliniczna | This study was excluded because it did not provide the exposure on Tg/glucose or Tg/HDL index values. |
| 77 | Association of serum lipid indices with large artery atherosclerotic stroke | 2008 | Neurology | This study was excluded because it did not provide the exposure on Tg/glucose or Tg/HDL index values. |
| 78 | Metabolic syndrome and atherogenic indices in school children: A worrying panorama in Brazil | 2017 | Diabetes metab syndr | This study was excluded because it did not provide the exposure on Tg/glucose or Tg/HDL index values. |
| 79 | Distinguishing between obese patients with type 1 diabetes (T1DMOB) and type 2 diabetes in adolescence (T2DMAD) at presentation | 2020 | Diabetes | This study was excluded because it did not provide the exposure on Tg/glucose or Tg/HDL index values. |
| 80 | Preterm twins with discordant birth weight: Auxological, hormonal and metabolic follow-up during the first two years of life | 2013 | Hormone Research in Paediatrics | This study was excluded because it did not provide the exposure on Tg/glucose or Tg/HDL index values. |
| 81 | Summary measures of the insulin resistance syndrome are adverse among Mexican-American versus non-Hispanic white children - The Corpus Christi Child Heart Study | 1997 | Circulation | This study was excluded because it did not provide the exposure on Tg/glucose or Tg/HDL index values. |
| 82 | Summary measures of the insulin resistance syndrome are adverse among Mexican-American versus non-Hispanic white children: The Corpus Christi Child Heart Study | 1997 | Circulation | This study was excluded because it did not provide the exposure on Tg/glucose or Tg/HDL index values. |
| 83 | Paraoxonase lactonase activity (PON-HTLase), asymmetric dimethylarginine (ADMA) and platelet activating factor-acetylhydrolase (PAF-AH) activity in non-obese women with PCOS | 2012 | Gynecological endocrinology | This study was excluded because it did not provide the exposure on Tg/glucose or Tg/HDL index values. |
| 84 | Evaluation of the prevalence of metabolic obesity and normal weight among the Polish population | 2012 | Endokrynologia polska | This study was excluded because it did not provide the exposure on Tg/glucose or Tg/HDL index values. |
| 85 | Association of serum lipids with β-cell function in obese children and adolescents | 2019 | Endocrine connections | This study was excluded because it did not provide the exposure on Tg/glucose or Tg/HDL index values. |
| 86 | Evaluation of TG-HDL ratio instead of HOMA ratio as insulin resistance marker in overweight and children with obesity | 2019 | Endocr metab immune disord drug targets | Included |
| 87 | Long-term effect of early postnatal overnutrition on insulin resistance and serum fatty acid profiles in male rats | 2015 | Lipids in Health and Disease | This study was excluded because it did not provide the exposure on Tg/glucose or Tg/HDL index values. |
| 88 | Body mass index and waist circumference: Relationship to cardiometabolic risk factors in children - Busselton Health Study 2005-2007 | 2013 | Journal of paediatrics and child health | This study was excluded because it did not provide the exposure on Tg/glucose or Tg/HDL index values. |
| 89 | Effects of nannochloropsis fed on serum and tissue lipids metabolism in obese offspring of overfed dams | 2019 | Current Nutrition and Food Science | This study was excluded because it did not provide the exposure on Tg/glucose or Tg/HDL index values. |
| 90 | Impaired insulin sensitivity and elevated ectopic fat in healthy obese vs. Nonobese prepubertal children | 2012 | Obesity | This study was excluded because it did not provide the exposure on Tg/glucose or Tg/HDL index values. |
| 91 | Insulin resistance syndrome in adolescents | 1996 | Metabolism-clinical and experimental | This study was excluded because it did not provide the exposure on Tg/glucose or Tg/HDL index values. |
| 92 | Classification and clinical characterization of metabolically "healthy" obese children and adolescents | 2016 | Journal of Pediatric Endocrinology and Metabolism | This study was excluded because it did not provide the exposure on Tg/glucose or Tg/HDL index values. |
| 93 | The shape of the plasma glucose curve during an oral glucose tolerance test as an indicator of beta cell function and insulin sensitivity in end-pubertal obese girls | 2015 | Hormone and Metabolic Research | This study was excluded because it did not provide the exposure on Tg/glucose or Tg/HDL index values. |
| 94 | A four-stage model explaining the higher risk of Type 2 diabetes mellitus in South Asians compared with European populations | 2013 | Diabetic medicine | This study was excluded because it did not provide the exposure on Tg/glucose or Tg/HDL index values. |
| 95 | Metabolic unhealthiness increases the likelihood of having metabolic syndrome components in Normoweight young adults | 2019 | International Journal of Environmental Research and Public Health | This study was excluded because it did not provide the exposure on Tg/glucose or Tg/HDL index values. |
| 96 | Evaluation of children with type 1 diabetes mellitus in terms of overweight/obesity in tertiary care hospital | 2021 | Journal of Pediatric Endocrinology and Metabolism | This study was excluded because it did not provide the exposure on Tg/glucose or Tg/HDL index values. |
| 97 | Serum lipids, glucose homeostasis and abdominal adipose tissue distribution in protease inhibitor-treated and naive HIV-infected children | 2003 | Aids | This study was excluded because it did not provide the exposure on Tg/glucose or Tg/HDL index values. |
| 98 | Potential of chlorella as a dietary supplement to promote human health | 2020 | Nutrients | This study was excluded because it did not provide the exposure on Tg/glucose or Tg/HDL index values. |
| 99 | Association between sdb and metabolic syndrome in adolescents: The Penn State Child Cohort | 2016 | Sleep | This study was excluded because it did not provide the exposure on Tg/glucose or Tg/HDL index values. |
| 100 | Lipid profile and left ventricular geometry pattern in obese children | 2020 | Lipids in health and disease | This study was excluded because it did not provide the exposure on Tg/glucose or Tg/HDL index values. |
| 101 | Insulin resistance surrogates and left ventricular hypertrophy in normotensive obese children | 2021 | Cardiology in the Young | This study was excluded because it did not provide the exposure on Tg/glucose or Tg/HDL index values. |
| 102 | Pathogenesis of Lipid Disorders in Insulin Resistance: a Brief Review | 2018 | Current diabetes reports | This study was excluded because it did not provide the exposure on Tg/glucose or Tg/HDL index values. |
| 103 | Insulin sensitivity and complications in type 1 diabetes: New insights | 2015 | World Journal of Diabetes | This study was excluded because it did not provide the exposure on Tg/glucose or Tg/HDL index values. |
| 104 | Brain-Derived Neurotropic Factor, Vascular Endothelial Growth Factor and Matrix Metalloproteinases as Markers of Metabolic Status in Non-Growth Hormone-Treated Girls With Turner Syndrome | 2021 | Frontiers in endocrinology | This study was excluded because it did not provide the exposure on Tg/glucose or Tg/HDL index values. |
| 105 | Association of interleukin-6 polymorphisms with obesity or metabolic traits in young Mexican-Americans | 2018 | Obesity Science and Practice | This study was excluded because it did not provide the exposure on Tg/glucose or Tg/HDL index values. |
| 106 | Metabolic syndrome in childhood: Association with birth weight, maternal obesity, and gestational diabetes mellitus | 2005 | Pediatrics | This study was excluded because it did not provide the exposure on Tg/glucose or Tg/HDL index values. |
| 107 | Hyperinsulinemia and insulin resistance are independently associated with plasma lipids, uric acid and blood pressure in non-diabetic subjects. The GISIR database | 2008 | Nutrition, Metabolism and Cardiovascular Diseases | This study was excluded because it did not provide the exposure on Tg/glucose or Tg/HDL index values. |
| 108 | Association of MTHFR C677T and ABCA1 G656A polymorphisms with obesity among Egyptian children | 2018 | Gene reports | This study was excluded because it did not provide the exposure on Tg/glucose or Tg/HDL index values. |
| 109 | Effects of a program of vigorous-to-intense physical activity in triglycerides and glucose in 3- to 16-year-old schoolchildren | 2018 | Journal of human sport and exercise | This study was excluded because it did not provide the exposure on Tg/glucose or Tg/HDL index values. |
| 110 | Factors influencing insulin resistance in relation to atherogenicity in mood disorders, the metabolic syndrome and tobacco use disorder | 2015 | Journal of Affective Disorders | This study was excluded because it did not provide the exposure on Tg/glucose or Tg/HDL index values. |
| 111 | Birth weight, current anthropometric markers, and high sensitivity C-reactive protein in brazilian school children | 2015 | Journal of Obesity | This study was excluded because it did not provide the exposure on Tg/glucose or Tg/HDL index values. |
| 112 | Thyroid function and metabolic risk factors in obese youth. Changes during follow-up: a preventive mechanism? | 2014 | Experimental and clinical endocrinology & diabetes : official journal, German Society of Endocrinology [and] German Diabetes Association | This study was excluded because it did not provide the exposure on Tg/glucose or Tg/HDL index values. |
| 113 | Ramadan Fasting's Effect on Plasma Leptin, Adiponectin Concentrations, and Body Composition in Trained Young Men | 2008 | International journal of sport nutrition and exercise metabolism | This study was excluded because it did not provide the exposure on Tg/glucose or Tg/HDL index values. |
| 114 | Effects of infliximab treatment in terms of cardiovascular risk and insulin resistance in ankylosing spondylitis patients | 2014 | Modern rheumatology | This study was excluded because it did not provide the exposure on Tg/glucose or Tg/HDL index values. |
| 115 | Association of low birth weight with beta cell and kidney functions in adults without diabetes | 2019 | Diabetology and Metabolic Syndrome | This study was excluded because it did not provide the exposure on Tg/glucose or Tg/HDL index values. |
| 116 | Remnant cholesterol in individuals with type 2 diabetes: Correlation to components of the metabolic syndrome and triglyceride-glucose index in the DIVE and DPV registries | 2020 | Diabetologia | This study was excluded because it did not provide the exposure on Tg/glucose or Tg/HDL index values. |
| 117 | Obese Mexican American Children Have Elevated MCP-1, TNF-alpha, Monocyte Concentration, and Dyslipidemia | 2012 | Pediatrics | This study was excluded because it did not provide the exposure on Tg/glucose or Tg/HDL index values. |
| 118 | Diastolic blood pressure changes during exercise positively correlate with serum cholesterol and insulin resistance | 2000 | Circulation | This study was excluded because it did not provide the exposure on Tg/glucose or Tg/HDL index values. |
| 119 | Use of the triglyceride to HDL cholesterol ratio for assessing insulin sensitivity in overweight and obese children in rural Appalachia | 2016 | J pediatr endocrinol metab | Included |
| 120 | Predictive capacity of triglyceride-glucose (TyG) index for insulin resistance and cardiometabolic risk in children and adolescents: a systematic review | 2021 | Critical reviews in food science and nutrition | This study was excluded because it did not provide the exposure on Tg/glucose or Tg/HDL index values. |
| 121 | TAG-glucose (TyG) index in childhood: an estimate of cut-off points and the relation to cardiometabolic risk in 4- to 9-year-old children | 2021 | Public health nutr | Included |
| 122 | Τriglycerides-glucose (TyG) index is a sensitive marker of insulin resistance in Greek children and adolescents | 2020 | Endocrine | Included |
| 123 | The role of fitness in the association between fatness and cardiometabolic risk from childhood to adolescence | 2013 | Pediatric diabetes | This study was excluded because it did not provide the exposure on Tg/glucose or Tg/HDL index values. |
| 124 | Healthy pre-monopausal women with type 1 diabetes have increased peripheral Insulin resistance compared to non diabetic women | 2012 | Diabetes | This study was excluded because it did not provide the exposure on Tg/glucose or Tg/HDL index values. |
| 125 | Intramyocellular lipid is associated with visceral adiposity, markers of insulin resistance, and cardiovascular risk in prepubertal children: The EPOCH study | 2012 | Journal of Clinical Endocrinology and Metabolism | This study was excluded because it did not provide the exposure on Tg/glucose or Tg/HDL index values. |
| 126 | Utility of Body Mass Index, Waist-to-Height-Ratio and cardiorespiratory fitness thresholds for identifying cardiometabolic risk in 10.4-17.6-year-old children | 2017 | Obes res clin pract | This study was excluded because it did not provide the exposure on Tg/glucose or Tg/HDL index values. |
| 127 | Utility of three anthropometric indices in assessing the cardiometabolic risk profile in children | 2017 | Am j hum biol | This study was excluded because it did not provide the exposure on Tg/glucose or Tg/HDL index values. |
| 128 | Weight loss in obese African American and Caucasian adolescents: secondary analysis of a randomized clinical trial of behavioral therapy plus sibutramine | 2007 | The Journal of cardiovascular nursing | This study was excluded because it did not provide the exposure on Tg/glucose or Tg/HDL index values. |
| 129 | Metabolic risk-factor clustering estimation in obese children | 2007 | Journal of Physiology and Biochemistry | This study was excluded because it did not provide the exposure on Tg/glucose or Tg/HDL index values. |
| 130 | The role of irisin in the relationship between psoriasis and insulin resistance | 2018 | Giornale italiano di dermatologia e venereologia | This study was excluded because it did not provide the exposure on Tg/glucose or Tg/HDL index values. |
| 131 | Intrauterine exposure to diabetes is a determinant of hemoglobin A(1c) and systolic blood pressure in Pima Indian children | 2005 | Journal of clinical endocrinology & metabolism | This study was excluded because it did not provide the exposure on Tg/glucose or Tg/HDL index values. |
| 132 | Surrogate Lipid Markers for Small Dense Low-Density Lipoprotein Particles in Overweight Youth | 2012 | Journal of pediatrics | This study was excluded because it did not provide the exposure on Tg/glucose or Tg/HDL index values. |
| 133 | [Metabolic syndrome prevalence in Chilean children and adolescent with family history of chronic noncommunicable diseases] | 2012 | Arch latinoam nutr | This study was excluded because it did not provide the exposure on Tg/glucose or Tg/HDL index values. |
| 134 | The anthropometric and cardiometabolic profile of adolescence is associated with increased risk of metabolic syndrome in early adulthood | 2016 | Hormone Research in Paediatrics | This study was excluded because it did not provide the exposure on Tg/glucose or Tg/HDL index values. |
| 135 | Cardiometabolic risk in healthy Chilean adolescents: Influence of physical inactivity and sarcopeni1 | 2014 | Pediatric diabetes | This study was excluded because it did not provide the exposure on Tg/glucose or Tg/HDL index values. |
| 136 | Low bone mass gain from adolescence to emerging adulthood is associated with obesity, insulin resistance and cardiometabolic profile at age 16 | 2017 | Hormone Research in Paediatrics | This study was excluded because it did not provide the exposure on Tg/glucose or Tg/HDL index values. |
| 137 | Clustered cardiometabolic risk from adolescence to emerging adulthood in chilean infancy cohort: The role of insulin resistance | 2017 | Hormone Research in Paediatrics | This study was excluded because it did not provide the exposure on Tg/glucose or Tg/HDL index values. |
| 138 | Cardio-metabolic risk profile in Chilean adolescents of mid-low socioeconomic level: Association with sex and obesity | 2013 | Hormone Research in Paediatrics | This study was excluded because it did not provide the exposure on Tg/glucose or Tg/HDL index values. |
| 139 | Cardiovascular risk in chilean adolescents: The role of family history of type 2 diabetes and obesity | 2012 | Hormone Research in Paediatrics | This study was excluded because it did not provide the exposure on Tg/glucose or Tg/HDL index values. |
| 140 | Cardiovascular risk factors in overweight children with family history (FH) for non transmissible chronic diseases | 2010 | Hormone Research in Paediatrics | This study was excluded because it did not provide the exposure on Tg/glucose or Tg/HDL index values. |
| 141 | Association of dietary patterns with insulin resistance and clinically silent carotid atherosclerosis in apparently healthy people | 2013 | European Journal of Clinical Nutrition | This study was excluded because it did not provide the exposure on Tg/glucose or Tg/HDL index values. |
| 142 | Genetic and environmental factors influencing fasting serum adiponectin in hispanic children | 2005 | Journal of Clinical Endocrinology and Metabolism | This study was excluded because it did not provide the exposure on Tg/glucose or Tg/HDL index values. |
| 143 | Quantitative genetic analysis of the metabolic syndrome in Hispanic children | 2005 | Pediatric research | This study was excluded because it did not provide the exposure on Tg/glucose or Tg/HDL index values. |
| 144 | Pediatric Loss-of-Control Eating and Anxiety in Relation to Components of Metabolic Syndrome | 2019 | Journal of pediatric psychology | This study was excluded because it did not provide the exposure on Tg/glucose or Tg/HDL index values. |
| 145 | Comparison of HOMA-IR ratio and TG/HDL-C ratio for the diagnosis of metabolic syndrome in obese 3-5 years old children | 2016 | Hormone Research in Paediatrics | This study was excluded because it did not provide the exposure on Tg/glucose or Tg/HDL index values. |
| 146 | Hemodynamics and Metabolism at Low versus Moderate Altitudes | 2011 | High altitude medicine & biology | This study was excluded because it did not provide the exposure on Tg/glucose or Tg/HDL index values. |
| 147 | Frequency and characteristics of metabolic syndrome and insulin resistance in the first-degree relatives of persons with type 1 diabetes | 2010 | Endocrinologia y Nutricion | This study was excluded because it did not provide the exposure on Tg/glucose or Tg/HDL index values. |
| 148 | Metabolic parameters prior to the onset of psychosis in the north American prodrome longitudinal studies (NAPLS) consortium | 2015 | Schizophrenia bulletin | This study was excluded because it did not provide the exposure on Tg/glucose or Tg/HDL index values. |
| 149 | An assessment of the hemorheological profile in patients with subclinical carotid atherosclerosis divided in relation to the number of cardiovascular risk factors and different degrees of insulin resistance | 2021 | Clinical Hemorheology and Microcirculation | This study was excluded because it did not provide the exposure on Tg/glucose or Tg/HDL index values. |
| 150 | Cardiometabolic outcomes in children and adolescents following discontinuation of long-term risperidone treatment | 2014 | Journal of Child and Adolescent Psychopharmacology | This study was excluded because it did not provide the exposure on Tg/glucose or Tg/HDL index values. |
| 151 | Non-thyroidal illness syndrome and SARS-CoV-2-associated multisystem inflammatory syndrome in children | 2021 | Journal of Endocrinological Investigation | This study was excluded because it did not provide the exposure on Tg/glucose or Tg/HDL index values. |
| 152 | Impaired Glucose-Insulin Metabolism in Multisystem Inflammatory Syndrome Related to SARS-CoV-2 in Children | 2021 | Children-basel | This study was excluded because it did not provide the exposure on Tg/glucose or Tg/HDL index values. |
| 153 | Diabetes type 2 in neurologically impaired children and adolescents without obesity: A new emerging entity? | 2019 | Frontiers in Neurology | This study was excluded because it did not provide the exposure on Tg/glucose or Tg/HDL index values. |
| 154 | Triglyceride Glucose Index as a Surrogate Measure of Insulin Sensitivity in a Caucasian Pediatric Population | 2019 | Journal of clinical research in pediatric endocrinology | Included |
| 155 | Diabetes type 2 in non-obese neurologically impaired children and adolescents: A new emerging entity? | 2019 | Hormone Research in Paediatrics | This study was excluded because it did not provide the exposure on Tg/glucose or Tg/HDL index values. |
| 156 | Sex-Specific Differences in the Relationship between Insulin Resistance and Adiposity Indexes in Children and Adolescents with Obesity | 2021 | Children-basel | This study was excluded because it did not provide the exposure on Tg/glucose or Tg/HDL index values. |
| 157 | Lifestyle Behaviors in Metabolically Healthy and Unhealthy Overweight and Obese Women: A Preliminary Study | 2015 | Plos one | This study was excluded because it did not provide the exposure on Tg/glucose or Tg/HDL index values. |
| 158 | Association of Metabolic Risk with Longitudinal Physical Activity and Fitness: Coronary Artery Risk Development in Young Adults (CARDIA) | 2013 | Metabolic syndrome and related disorders | This study was excluded because it did not provide the exposure on Tg/glucose or Tg/HDL index values. |
| 159 | Physical activity and screen time in metabolically healthy obese phenotypes in adolescents and adults | 2013 | Journal of obesity | This study was excluded because it did not provide the exposure on Tg/glucose or Tg/HDL index values. |
| 160 | Effect of a fruit and vegetable juice concentrate (FVJC) Vs. medical nutrition therapy alone on metabolic syndrome components in obese children: A 6 month pilot double blind placebo-controlled study | 2011 | Faseb journal | This study was excluded because it did not provide the exposure on Tg/glucose or Tg/HDL index values. |
| 161 | Visceral adipose index (VAI) can accurately predict markers of cardiometabolic risk in children | 2018 | Endocrine reviews | This study was excluded because it did not provide the exposure on Tg/glucose or Tg/HDL index values. |
| 162 | Association between lipid profile, BMI, and insulin resistance markers in obese prepubertal children | 2014 | Hormone Research in Paediatrics | This study was excluded because it did not provide the exposure on Tg/glucose or Tg/HDL index values. |
| 163 | The metabolic effects of antipsychotics in the early stage of treatment in first-episode patients with schizophrenia: A real-world study in a naturalistic setting | 2020 | Journal of psychiatric research | This study was excluded because it did not provide the exposure on Tg/glucose or Tg/HDL index values. |
| 164 | Insulin Resistance in Healthy US Adults: Findings from the National Health and Nutrition Examination Survey (NHANES) | 2020 | Cancer epidemiology biomarkers & prevention | This study was excluded because it did not provide the exposure on Tg/glucose or Tg/HDL index values. |
| 165 | Plasma interteukin-6 levels are increased in subjects with impaired glucose tolerance but not in those with impaired fasting glucose in a cohort of Italian Caucasians | 2007 | Diabetes-metabolism research and reviews | This study was excluded because it did not provide the exposure on Tg/glucose or Tg/HDL index values. |
| 166 | Plasma interleukin-6 levels are increased in subjects with impaired glucose tolerance but not in those with impaired fasting glucose in a cohort of Italian Caucasians | 2007 | Diabetes/Metabolism Research and Reviews | This study was excluded because it did not provide the exposure on Tg/glucose or Tg/HDL index values. |
| 167 | Carotid artery intima-media thickness is associated with insulin-mediated glucose disposal in nondiabetic normotensive offspring of type 2 diabetic patients | 2007 | American journal of physiology-endocrinology and metabolism | This study was excluded because it did not provide the exposure on Tg/glucose or Tg/HDL index values. |
| 168 | Dietary Fiber and Nutrient Density Are Inversely Associated with the Metabolic Syndrome in US Adolescents | 2011 | Journal of the american dietetic association | This study was excluded because it did not provide the exposure on Tg/glucose or Tg/HDL index values. |
| 169 | Oxidative stress markers in preschools from 3 to 5 years old with obesity in a CDMX Hospital | 2018 | Obesity facts | This study was excluded because it did not provide the exposure on Tg/glucose or Tg/HDL index values. |
| 170 | The prevalence of obesity and metabolic syndrome in paediatric patients with epilepsy treated in monotherapy with valproic acid | 2015 | Revista de neurologia | This study was excluded because it did not provide the exposure on Tg/glucose or Tg/HDL index values. |
| 171 | Compositional analyses of the associations between sedentary time, different intensities of physical activity, and cardiometabolic biomarkers among children and youth from the United States | 2019 | Plos One | This study was excluded because it did not provide the exposure on Tg/glucose or Tg/HDL index values. |
| 172 | Differential Influence of Diet and Physical Activity on Components of Metabolic Syndrome in a Multiethnic Sample of Children | 2009 | Journal of the american dietetic association | This study was excluded because it did not provide the exposure on Tg/glucose or Tg/HDL index values. |
| 173 | Effects of normalization of GH hypersecretion on lipoprotein(a) and other lipoprotein serum levels in acromegaly | 2000 | Clinical endocrinology | This study was excluded because it did not provide the exposure on Tg/glucose or Tg/HDL index values. |
| 174 | Serum fatty acid-binding protein 4 levels are higher in lean adolescents with insulin resistance | 2021 | Atherosclerosis | This study was excluded because it did not provide the exposure on Tg/glucose or Tg/HDL index values. |
| 175 | Effects of L-thyroxine treatment on early markers of atherosclerotic disease in children with subclinical hypothyroidism | 2016 | European journal of endocrinology | This study was excluded because it did not provide the exposure on Tg/glucose or Tg/HDL index values. |
| 176 | Cardiovascular Risk Factors in Children With Long-Standing Untreated Idiopathic Subclinical Hypothyroidism | 2014 | Journal of clinical endocrinology & metabolism | This study was excluded because it did not provide the exposure on Tg/glucose or Tg/HDL index values. |
| 177 | Adipocytokines, leptin/adiponectin ratio and cardiovascular risk factors in Portuguese adolescents: The labmed physical activity study | 2016 | Atencion primaria | This study was excluded because it did not provide the exposure on Tg/glucose or Tg/HDL index values. |
| 178 | Are metabolic syndrome antecedents in prepubertal children associated with being born idiopathic large for gestational age? | 2013 | Pediatric diabetes | This study was excluded because it did not provide the exposure on Tg/glucose or Tg/HDL index values. |
| 179 | Evaluation of early markers of risk for metabolic syndrome and vascular function in children with intrauterine growth restriction | 2013 | Hormone Research in Paediatrics | This study was excluded because it did not provide the exposure on Tg/glucose or Tg/HDL index values. |
| 180 | Ala54Thr polymorphism of the fatty acid binding protein 2 gene and saturated fat intake in relation to lipid levels and insulin resistance: the Coronary Artery Risk Development in Young Adults (CARDIA) study | 2009 | Metabolism-clinical and experimental | This study was excluded because it did not provide the exposure on Tg/glucose or Tg/HDL index values. |
| 181 | Effect of supervised exercise intervention on metabolic risk factors and physical fitness in Chinese obese children in early puberty | 2008 | Obesity reviews | This study was excluded because it did not provide the exposure on Tg/glucose or Tg/HDL index values. |
| 182 | Evidence in Obese Children: Contribution of Hyperlipidemia, Obesity-Inflammation, and Insulin Sensitivity | 2015 | Plos ONE | This study was excluded because it did not provide the exposure on Tg/glucose or Tg/HDL index values. |
| 183 | Cardiovascular risk markers in metabolically healthy and metabolically unhealthy obese adolescents | 2014 | Hormone Research in Paediatrics | This study was excluded because it did not provide the exposure on Tg/glucose or Tg/HDL index values. |
| 184 | Cross-sectional and longitudinal associations between serum uric acid and metabolic syndrome: Results from Fangchenggang Area Male Health and Examination Survey in China | 2014 | Clin chim acta | This study was excluded because it did not provide the exposure on Tg/glucose or Tg/HDL index values. |
| 185 | Unsupportive parenting moderates the effects of family psychosocial intervention on metabolic syndrome in African American youth | 2014 | Int j obes (lond) | This study was excluded because it did not provide the exposure on Tg/glucose or Tg/HDL index values. |
| 186 | COVID-19 May Increase the Risk of Insulin Resistance in Adult Patients Without Diabetes: A 6-Month Prospective Study | 2021 | Endocrine practice | This study was excluded because it did not provide the exposure on Tg/glucose or Tg/HDL index values. |
| 187 | Relationship between waist circumference and body mass index and metabolic syndrome related traits among middle school students in Beijing | 2021 | Beijing da xue xue bao yi xue ban | This study was excluded because it did not provide the exposure on Tg/glucose or Tg/HDL index values. |
| 188 | Age-related patterns of the clustering of cardiovascular risk variables of Syndrome X from childhood to young adulthood in a population made up of black and white subjects: The Bogalusa Heart Study | 2000 | Diabetes | This study was excluded because it did not provide the exposure on Tg/glucose or Tg/HDL index values. |
| 189 | Age-belated patterns of the clustering of cardiovascular risk variables of Syndrome X from childhood to young adulthood in a population made up of black and white subjects - The Bogalusa Heart Study | 2000 | Diabetes | This study was excluded because it did not provide the exposure on Tg/glucose or Tg/HDL index values. |
| 190 | Cardiovascular risk factors clustering features of insulin resistance syndrome (Syndrome X) in a biracial (Black-White) population of children, adolescents, and young adults: The Bogalusa Heart Study | 1999 | American Journal of Epidemiology | This study was excluded because it did not provide the exposure on Tg/glucose or Tg/HDL index values. |
| 191 | The association of cardiovascular risk factor clustering related to insulin resistance syndrome (Syndrome X) between young parents and their offspring: The Bogalusa Heart Study | 1999 | Atherosclerosis | This study was excluded because it did not provide the exposure on Tg/glucose or Tg/HDL index values. |
| 192 | Cardiovascular risk factors clustering related to insulin resistance syndrome(Syndrome x) in parents and their young offspring: The bogalusa heart study | 1999 | Journal of Investigative Medicine | This study was excluded because it did not provide the exposure on Tg/glucose or Tg/HDL index values. |
| 193 | Clustering of long-term trends in metabolic syndrome variables from childhood to adulthood in Blacks and Whites: The Bogalusa Heart Study | 2007 | American Journal of Epidemiology | This study was excluded because it did not provide the exposure on Tg/glucose or Tg/HDL index values. |
| 194 | Comparative effectiveness of switching antipsychotic drug treatment to aripiprazole or ziprasidone for improving metabolic profile and atherogenic dyslipidemia: a 12-month, prospective, open-label study | 2012 | Journal of psychopharmacology | This study was excluded because it did not provide the exposure on Tg/glucose or Tg/HDL index values. |
| 195 | Association between alpha-fetoprotein and metabolic syndrome in a Chinese asymptomatic population: a cross-sectional study | 2016 | Lipids in health and disease | This study was excluded because it did not provide the exposure on Tg/glucose or Tg/HDL index values. |
| 196 | The effects of muscle mass and muscle quality on cardio-metabolic risk in peripubertal girls: a longitudinal study from childhood to early adulthood | 2018 | International journal of obesity | This study was excluded because it did not provide the exposure on Tg/glucose or Tg/HDL index values. |
| 197 | Vitamin D deficiency is associated with atherosclerosis-promoting risk factor clustering but not vascular damage in children | 2012 | Medical science monitor | This study was excluded because it did not provide the exposure on Tg/glucose or Tg/HDL index values. |
| 198 | The risk of metabolic syndrome across tertile of delta changes in SDS-height during growth hormone therapy in pre-pubertal children with growth hormone deficiency (GHD) | 2017 | Hormone Research in Paediatrics | This study was excluded because it did not provide the exposure on Tg/glucose or Tg/HDL index values. |
| 199 | Evaluation index triglycerides and glucose as a marker of insulin resistance and its comparison with other markers of insulin resistance in obese children | 2016 | Hormone Research in Paediatrics | This study was excluded because it did not provide the exposure on Tg/glucose or Tg/HDL index values. |
| 200 | Tryglicerides /HDL index in a pediatric population from the city of Rosario and its surrounding area | 2018 | Revista Medica de Rosario | This study was excluded because it did not provide the exposure on Tg/glucose or Tg/HDL index values. |
| 201 | Progression of cardio-metabolic risk factors in subjects born small and large for gestational age | 2014 | Plos ONE | This study was excluded because it did not provide the exposure on Tg/glucose or Tg/HDL index values. |
| 202 | Fat distribution, inflammatory biomarkers, and cardiometabolic risk in young adults: The avon longitudinal study of parents and children | 2019 | Circulation | This study was excluded because it did not provide the exposure on Tg/glucose or Tg/HDL index values. |
| 203 | The Metabolic Syndrome in Obese Children | 2004 | Pediatric gastroenterology, hepatology & nutrition | This study was excluded because it did not provide the exposure on Tg/glucose or Tg/HDL index values. |
| 204 | Metabolic syndrome components of normal weight central obese adolescents in Korea stratified by waist-to-height ratio: Results from k-nhanes 2008-2010 | 2015 | Hormone Research in Paediatrics | This study was excluded because it did not provide the exposure on Tg/glucose or Tg/HDL index values. |
| 205 | Association of insulin resistance with near peak bone mass in the femur and lumbar spine of Korean adults aged 25-35: The Korean National Health and Nutrition Examination Survey 2008-2010 | 2017 | Plos ONE | This study was excluded because it did not provide the exposure on Tg/glucose or Tg/HDL index values. |
| 206 | Atherogenic index of plasma is related to arterial stiffness but not to blood pressure in normotensive and never-treated hypertensive subjects | 2019 | Blood pressure | This study was excluded because it did not provide the exposure on Tg/glucose or Tg/HDL index values. |
| 207 | Influence of genetic variation on metabolic outcomes among survivors of pediatric hematopoietic cell transplantation | 2013 | Blood | This study was excluded because it did not provide the exposure on Tg/glucose or Tg/HDL index values. |
| 208 | Waist to hip ratio versus obesity severity in predicting cardiometabolic risk in children | 2016 | Journal of the American College of Cardiology | This study was excluded because it did not provide the exposure on Tg/glucose or Tg/HDL index values. |
| 209 | Adipocytokines concentration and metabolic parameters in obese children | 2011 | Pediatric Endocrinology, Diabetes and Metabolism | This study was excluded because it did not provide the exposure on Tg/glucose or Tg/HDL index values. |
| 210 | Plasma leptin, fatty acids, and tumor necrosis factor-receptor and insulin resistance in children | 2003 | Obesity research | This study was excluded because it did not provide the exposure on Tg/glucose or Tg/HDL index values. |
| 211 | Relationship between plasma adiponectin levels and metabolic risk profiles in Taiwanese children | 2005 | Obesity research | This study was excluded because it did not provide the exposure on Tg/glucose or Tg/HDL index values. |
| 212 | Plasma leptin concentrations and obesity in relation to insulin resistance syndrome components among school children in Taiwan - The Taipei Children Heart Study | 2000 | International Journal of Obesity | This study was excluded because it did not provide the exposure on Tg/glucose or Tg/HDL index values. |
| 213 | Risk assessment of metabolic syndrome in adolescents using the triglyceride/high-density lipoprotein cholesterol ratio and the total cholesterol/high-density lipoprotein cholesterol ratio | 2019 | Annals of Pediatric Endocrinology and Metabolism | This study was excluded because it did not provide the exposure on Tg/glucose or Tg/HDL index values. |
| 214 | Hemoglobin A1c in the Screening of Obesity- Related Diseases in Children and Adolescents | 2012 | Annals of Pediatirc Endocrinology & Metabolism | This study was excluded because it did not provide the exposure on Tg/glucose or Tg/HDL index values. |
| 215 | High triglyceride to high-density lipoprotein cholesterol ratio and arterial stiffness in postmenopausal Korean women | 2019 | Journal of Clinical Hypertension | This study was excluded because it did not provide the exposure on Tg/glucose or Tg/HDL index values. |
| 216 | Changes in serum lipid levels during pregnancy in women with gestational diabetes. A narrative review | 2021 | Biomedical papers of the Medical Faculty of the University Palacky, Olomouc, Czechoslovakia | This study was excluded because it did not provide the exposure on Tg/glucose or Tg/HDL index values. |
| 217 | Red yeast rice improves lipid pattern, high-sensitivity C-reactive protein, and vascular remodeling parameters in moderately hypercholesterolemic Italian subjects | 2013 | Nutrition research | This study was excluded because it did not provide the exposure on Tg/glucose or Tg/HDL index values. |
| 218 | Effects of phytosomal curcumin on anthropometric parameters, insulin resistance, cortisolemia and non-alcoholic fatty liver disease indices: a double-blind, placebo-controlled clinical trial | 2020 | European journal of nutrition | This study was excluded because it did not provide the exposure on Tg/glucose or Tg/HDL index values. |
| 219 | C-Reactive Protein Decrease After Postbariatric Abdominoplasty | 2012 | Inflammation | This study was excluded because it did not provide the exposure on Tg/glucose or Tg/HDL index values. |
| 220 | HMGB1 is increased in adolescents with polycystic ovary syndrome (PCOS) and decreases after treatment with myo-inositol (MYO) in combination with alpha-lipoic acid (ALA) | 2020 | Gynecological endocrinology | This study was excluded because it did not provide the exposure on Tg/glucose or Tg/HDL index values. |
| 221 | High mobility group box 1 (HMGB1) is increased in adolescents with polycystic ovarian syndrome (PCOS) and decreases after treatment with myo-inositol in combination with a-lipoic acid (MYO+ALA) | 2018 | Hormone Research in Paediatrics | This study was excluded because it did not provide the exposure on Tg/glucose or Tg/HDL index values. |
| 222 | Compensatory Hyperinsulinemia in Young Adults and the Risk of Future Diabetes: CARDIA 25-year Follow Up | 2020 | Metabolism: Clinical and Experimental | This study was excluded because it did not provide the exposure on Tg/glucose or Tg/HDL index values. |
| 223 | Association of body mass index, blood pressure and serum levels of triglycerides and high-density lipoprotein cholesterol in childhood with the insulin sensitivity index in young adulthood: A 13-year follow-up | 1996 | Journal of Cardiovascular Risk | This study was excluded because it did not provide the exposure on Tg/glucose or Tg/HDL index values. |
| 224 | Association of RBP4 genetic variants with childhood obesity and cardiovascular risk factors | 2016 | Pediatric diabetes | This study was excluded because it did not provide the exposure on Tg/glucose or Tg/HDL index values. |
| 225 | Associate factors of nonalcoholic fatty liver disease : Results from a population-based study | 2007 | Gastroenterology | This study was excluded because it did not provide the exposure on Tg/glucose or Tg/HDL index values. |
| 226 | Effects on metabolism and growth of Growth Hormone Treatment for three years in 36 children with Prader-Willi Syndrome | 2010 | Hormone Research in Paediatrics | This study was excluded because it did not provide the exposure on Tg/glucose or Tg/HDL index values. |
| 227 | Prevalence of high plasma triglyceride combined with low HDL-C levels and its association with smoking, hypertension, obesity, diabetes, sedentariness and LDL-C levels in the Canadian population | 1999 | Canadian journal of cardiology | This study was excluded because it did not provide the exposure on Tg/glucose or Tg/HDL index values. |
| 228 | Does family history of obesity, cardiovascular, and metabolic diseases influence onset and severity of childhood obesity? | 2018 | Frontiers in Endocrinology | This study was excluded because it did not provide the exposure on Tg/glucose or Tg/HDL index values. |
| 229 | Insulin Sensitivity and Cardiovascular and Anthropometric Profile in Adolescence as Predictors of Non Alcoholic Fatty Liver Disease in Early Adulthood Results from the Longitudinal Study Santiago | 2018 | Hormone research in paediatrics | This study was excluded because it did not provide the exposure on Tg/glucose or Tg/HDL index values. |
| 230 | Early obesity and future cardiometabolic risk in a latino cohort: A matter of timing or tracking? | 2017 | Hormone Research in Paediatrics | This study was excluded because it did not provide the exposure on Tg/glucose or Tg/HDL index values. |
| 231 | [Prevalence of the metabolic syndrome: comparison between ATPIII and IDF criteria in a feminine population with severe obesity] | 2017 | Acta med port | This study was excluded because it did not provide the exposure on Tg/glucose or Tg/HDL index values. |
| 232 | Predictors of the development of cardiometabolic abnormalities in youth with bipolar disorder on ziprasidone or placebo | 2009 | European neuropsychopharmacology | This study was excluded because it did not provide the exposure on Tg/glucose or Tg/HDL index values. |
| 233 | Cardiometabolic Risk of Second-Generation Antipsychotic Medications During First-Time Use in Children and Adolescents | 2009 | Jama-journal of the american medical association | This study was excluded because it did not provide the exposure on Tg/glucose or Tg/HDL index values. |
| 234 | Cardiometabolic Risk in Patients With First-Episode Schizophrenia Spectrum Disorders Baseline Results From the RAISE-ETP Study | 2014 | Jama psychiatry | This study was excluded because it did not provide the exposure on Tg/glucose or Tg/HDL index values. |
| 235 | Effect of growth on cardiometabolic status at 4 y of age | 2009 | American journal of clinical nutrition | This study was excluded because it did not provide the exposure on Tg/glucose or Tg/HDL index values. |
| 236 | Tryglicerides/HDL index is a likely insulin-resistance marker in children and adolescents | 2012 | Hormone Research in Paediatrics | This study was excluded because it did not provide the exposure on Tg/glucose or Tg/HDL index values. |
| 237 | Associations between long chain polyunsaturated fatty acids and cardiovascular risk factors in youth with Type 1 Diabetes: SEARCH nutrition ancillary study | 2016 | Faseb journal | This study was excluded because it did not provide the exposure on Tg/glucose or Tg/HDL index values. |
| 238 | Associations between long chain polyunsaturated fatty acids and cardiovascular lipid risk factors in youth with type 1 diabetes: SEARCH Nutrition Ancillary Study | 2017 | Journal of Diabetes and its Complications | This study was excluded because it did not provide the exposure on Tg/glucose or Tg/HDL index values. |
| 239 | Insulin resistance in youth without diabetes is not related to muscle mitochondrial dysfunction | 2017 | Journal of Clinical Endocrinology and Metabolism | This study was excluded because it did not provide the exposure on Tg/glucose or Tg/HDL index values. |
| 240 | Predicting insulin resistance in adolescent girls with PCOS | 2017 | Endocrine reviews | This study was excluded because it did not provide the exposure on Tg/glucose or Tg/HDL index values. |
| 241 | Sex differences in insulin sensitivity in youth without diabetes exist, but have no relationship with muscle mitochondrial function | 2016 | Diabetes | This study was excluded because it did not provide the exposure on Tg/glucose or Tg/HDL index values. |
| 242 | Insulin-resistance, acanthosis nigricans and obesity in adolescents | 2015 | Boletin de pediatria | This study was excluded because it did not provide the exposure on Tg/glucose or Tg/HDL index values. |
| 243 | An easy way to identify obese children and adolescents with metabolic risk | 2017 | Hormone Research in Paediatrics | This study was excluded because it did not provide the exposure on Tg/glucose or Tg/HDL index values. |
| 244 | Fasting triglycerides and glucose index in an unselected consecutive Italian population of outpatients | 2011 | Rivista Italiana della Medicina di Laboratorio | This study was excluded because it did not provide the exposure on Tg/glucose or Tg/HDL index values. |
| 245 | Combined influence of healthy diet and active lifestyle on cardiovascular disease risk factors in adolescents | 2014 | Scandinavian journal of medicine & science in sports | This study was excluded because it did not provide the exposure on Tg/glucose or Tg/HDL index values. |
| 246 | Insulin resistance in a HIV plus pediatric population | 2008 | Faseb journal | This study was excluded because it did not provide the exposure on Tg/glucose or Tg/HDL index values. |
| 247 | Does body mass index modify the association between physical activity and screen time with cardiometabolic risk factors in adolescents? Findings from a country-wide survey | 2017 | International Journal of Obesity | This study was excluded because it did not provide the exposure on Tg/glucose or Tg/HDL index values. |
| 248 | Assessment of insulin resistance in patients with diabetes mellitus type 1 between 7 and 22 years old | 2018 | Diabetology and Metabolic Syndrome | This study was excluded because it did not provide the exposure on Tg/glucose or Tg/HDL index values. |
| 249 | Metabolically healthy obesity in children enrolled in the CANadian Pediatric Weight management Registry (CANPWR): An exploratory secondary analysis of baseline data | 2021 | Clinical obesity | This study was excluded because it did not provide the exposure on Tg/glucose or Tg/HDL index values. |
| 250 | Prevalence of combined and noncombined dyslipidemia in an Iranian population | 2018 | Journal of clinical laboratory analysis | This study was excluded because it did not provide the exposure on Tg/glucose or Tg/HDL index values. |
| 251 | Diet combined with desserts with a low glycemic index/glycemic load has a positive effect on metabolic syndrome parameters in overweight/obese children | 2011 | Endocrine reviews | This study was excluded because it did not provide the exposure on Tg/glucose or Tg/HDL index values. |
| 252 | Prevalence of insulin resistance and association with metabolic risk factors and food consumption in adolescents â€” Recife/Brazil | 2020 | Revista Paulista de Pediatria | This study was excluded because it did not provide the exposure on Tg/glucose or Tg/HDL index values. |
| 253 | The association between cardiovascular risk factors and anthropometric obesity indicators in university students in Sao Luis in the State of Maranhao, Brazil | 2015 | Ciencia & saude coletiva | This study was excluded because it did not provide the exposure on Tg/glucose or Tg/HDL index values. |
| 254 | Usefulness of triglyceride to high density lipoprotein cholesterol ratio to identify endothelial dysfunction in obese pre-pubertal children | 2012 | Hormone Research in Paediatrics | This study was excluded because it did not provide the exposure on Tg/glucose or Tg/HDL index values. |
| 255 | Triglycerides-to-HDL ratio as a new marker of endothelial dysfunction in obese prepubertal children | 2014 | European Journal of Endocrinology | This study was excluded because it did not provide the exposure on Tg/glucose or Tg/HDL index values. |
| 256 | Could the triglyceride:HDL cholesterol ratio be considered a new marker of cardiovascular risk in obese children? | 2014 | Clinical lipidology | This study was excluded because it did not provide the exposure on Tg/glucose or Tg/HDL index values. |
| 257 | Cortisol and Cortisone in Early Childhood in Very-Low-Birthweight Infants and Term-Born Infants | 2021 | Hormone Research in Paediatrics | This study was excluded because it did not provide the exposure on Tg/glucose or Tg/HDL index values. |
| 258 | Components of the metabolic syndrome in early childhood in very-low-birth-weight infants and term small and appropriate for gestational age infants | 2015 | Pediatric research | This study was excluded because it did not provide the exposure on Tg/glucose or Tg/HDL index values. |
| 259 | Genetic Deletion of Syndecan-4 Alters Body Composition, Metabolic Phenotypes, and the Function of Metabolic Tissues in Female Mice Fed A High-Fat Diet | 2019 | Nutrients | This study was excluded because it did not provide the exposure on Tg/glucose or Tg/HDL index values. |
| 260 | [Serum vitamin D and metabolic risk factors in a group of Spanish schoolchildren] | 2019 | Nutr hosp | This study was excluded because it did not provide the exposure on Tg/glucose or Tg/HDL index values. |
| 261 | Hyperfibrinogenemia in overweight and obese children and adolescents | 2016 | Obesity reviews | This study was excluded because it did not provide the exposure on Tg/glucose or Tg/HDL index values. |
| 262 | The severity of the metabolic syndrome (METS) in childhood is associated with future mets severity, insulin resistance, and oxidative stress as young adults: The bogalusa heart study | 2017 | Hormone Research in Paediatrics | This study was excluded because it did not provide the exposure on Tg/glucose or Tg/HDL index values. |
| 263 | Ability Among Adolescents for the Metabolic Syndrome to Predict Elevations in Factors Associated with Type 2 Diabetes and Cardiovascular Disease: Data from the National Health and Nutrition Examination Survey 1999-2006 | 2010 | Metabolic syndrome and related disorders | This study was excluded because it did not provide the exposure on Tg/glucose or Tg/HDL index values. |
| 264 | Effect of the number of cardiovascular risk factors on the insulin resistance, C reactive protein and alanineaminotransferase in adolescents | 2011 | Journal of Diabetes | This study was excluded because it did not provide the exposure on Tg/glucose or Tg/HDL index values. |
| 265 | Higher metabolic syndrome in obese asthmatic compared to obese nonasthmatic adolescent males | 2010 | Journal of Asthma | This study was excluded because it did not provide the exposure on Tg/glucose or Tg/HDL index values. |
| 266 | Vitamin D status is modestly associated with glycemia and indicators of lipid metabolism in French-Canadian children and adolescents | 2010 | Journal of Nutrition | This study was excluded because it did not provide the exposure on Tg/glucose or Tg/HDL index values. |
| 267 | IGF2 methylation and metabolic risk in obese children | 2012 | Hormone Research in Paediatrics | This study was excluded because it did not provide the exposure on Tg/glucose or Tg/HDL index values. |
| 268 | IGF2 Methylation Is Associated with Lipid Profile in Obese Children | 2013 | Hormone research in paediatrics | This study was excluded because it did not provide the exposure on Tg/glucose or Tg/HDL index values. |
| 269 | High normal post-load plasma glucose, cardiometabolic risk factors and signs of organ damage in obese children | 2014 | Obesity | This study was excluded because it did not provide the exposure on Tg/glucose or Tg/HDL index values. |
| 270 | Usefulness of the high triglyceride-to-HDL cholesterol ratio to identify cardiometabolic risk factors and preclinical signs of organ damage in outpatient children | 2012 | Diabetes care | This study was excluded because it did not provide the exposure on Tg/glucose or Tg/HDL index values. |
| 271 | Impaired fasting glucose and impaired glucose tolerance in children and adolescents with overweight/obesity | 2017 | Journal of Endocrinological Investigation | This study was excluded because it did not provide the exposure on Tg/glucose or Tg/HDL index values. |
| 272 | Comparison of non-HDL-cholesterol versus triglycerides-to-HDL-cholesterol ratio in relation to cardiometabolic risk factors and preclinical organ damage in overweight/obese children: The CARITALY study | 2015 | Nutrition, Metabolism and Cardiovascular Diseases | This study was excluded because it did not provide the exposure on Tg/glucose or Tg/HDL index values. |
| 273 | Is the Oxygen Radical Absorbance Capacity (ORAC) a predictor of cardiovascular risk in obese children and adolescents? | 2021 | Journal of Pediatric Gastroenterology and Nutrition | This study was excluded because it did not provide the exposure on Tg/glucose or Tg/HDL index values. |
| 274 | Prevalence of the metabolic syndrome in a psychiatric hospital in Mexico | 2021 | Actas esp psiquiatr | This study was excluded because it did not provide the exposure on Tg/glucose or Tg/HDL index values. |
| 275 | Cardiorespiratory Fitness and Light-Intensity Physical Activity Are Independently Associated with Reduced Cardiovascular Disease Risk in Urban Black South African Women: A Cross-Sectional Study | 2016 | Metabolic syndrome and related disorders | This study was excluded because it did not provide the exposure on Tg/glucose or Tg/HDL index values. |
| 276 | Obesity as a Mediator of the Influence of Cardiorespiratory Fitness on Cardiometabolic Risk: A Mediation Analysis | 2014 | Diabetes care | This study was excluded because it did not provide the exposure on Tg/glucose or Tg/HDL index values. |
| 277 | Metabolic risk assessment in obese children using Hypertriglyceridemic waist (HTGW) phenotype. Can it be a useful screening marker? | 2019 | Hormone Research in Paediatrics | This study was excluded because it did not provide the exposure on Tg/glucose or Tg/HDL index values. |
| 278 | tau riglycerides-glucose (TyG) index is a sensitive marker of insulin resistance in Greek children and adolescents | 2020 | Endocrine | This study was excluded because it did not provide the exposure on Tg/glucose or Tg/HDL index values. |
| 279 | Î¤riglycerides-glucose (TyG) index is a sensitive marker of insulin resistance in Greek children and adolescents | 2020 | Endocrine | This study was excluded because it did not provide the exposure on Tg/glucose or Tg/HDL index values. |
| 280 | A population-based cross-sectional study of the association between facial morphology and cardiometabolic risk factors in adolescence | 2013 | Bmj open | This study was excluded because it did not provide the exposure on Tg/glucose or Tg/HDL index values. |
| 281 | Associations between adipocyte fatty acid-binding protein and clinical parameters in polycystic ovary syndrome | 2015 | Archives of gynecology and obstetrics | This study was excluded because it did not provide the exposure on Tg/glucose or Tg/HDL index values. |
| 282 | Metabolic Effects Associated to the Highly Active Antiretroviral Therapy (HAART) in AIDS Patients | 2009 | Brazilian journal of infectious diseases | This study was excluded because it did not provide the exposure on Tg/glucose or Tg/HDL index values. |
| 283 | The role of oxidative stress in cardiometabolic risk related to phthalate exposure in elderly diabetic patients from Shanghai | 2018 | Environment international | This study was excluded because it did not provide the exposure on Tg/glucose or Tg/HDL index values. |
| 284 | Duration of breastfeeding is an independent contributor to BMI z-score decrease after 12 months of lifestyle intervention in overweight and obese children | 2016 | Journal of Pediatric Gastroenterology and Nutrition | This study was excluded because it did not provide the exposure on Tg/glucose or Tg/HDL index values. |
| 285 | Metabolic Syndrome in Children: Comparison of the International Diabetes Federation 2007 Consensus with an Adapted National Cholesterol Education Program Definition in 300 Overweight and Obese French Children | 2010 | Hormone research in paediatrics | This study was excluded because it did not provide the exposure on Tg/glucose or Tg/HDL index values. |
| 286 | Associations between Thyroid Hormones Within the Euthyroid Range and Indices of Obesity in Obese Chinese Women of Reproductive Age | 2019 | Metabolic Syndrome and Related Disorders | This study was excluded because it did not provide the exposure on Tg/glucose or Tg/HDL index values. |
| 287 | The relation between salivary cortisol and the metabolic syndrome score in girls | 2013 | Journal of pediatric endocrinology & metabolism | This study was excluded because it did not provide the exposure on Tg/glucose or Tg/HDL index values. |
| 288 | Firefighters' Physical Activity: Relation to Fitness and Cardiovascular Disease Risk | 2011 | Medicine and science in sports and exercise | This study was excluded because it did not provide the exposure on Tg/glucose or Tg/HDL index values. |
| 289 | High Intensity Exercise: Can It Protect You from A Fast Food Diet? | 2017 | Nutrients | This study was excluded because it did not provide the exposure on Tg/glucose or Tg/HDL index values. |
| 290 | Differential effects of sugar-sweetened, artificially sweetened, and unsweetened beverages on taste preference but not CVD risk factors in a 12-month RCT | 2019 | Circulation | This study was excluded because it did not provide the exposure on Tg/glucose or Tg/HDL index values. |
| 291 | Effects of a low-carbohydrate diet on insulin-resistant dyslipoproteinemia-a randomized controlled feeding trial | 2021 | The American journal of clinical nutrition | This study was excluded because it did not provide the exposure on Tg/glucose or Tg/HDL index values. |
| 292 | Effects of raw red onion consumption on metabolic features in overweight or obese women with polycystic ovary syndrome: A randomized controlled clinical trial | 2014 | Journal of obstetrics and gynaecology research | This study was excluded because it did not provide the exposure on Tg/glucose or Tg/HDL index values. |
| 293 | Metabolic syndrome and related insulin levels in obese children | 2006 | Metabolic Syndrome and Related Disorders | This study was excluded because it did not provide the exposure on Tg/glucose or Tg/HDL index values. |
| 294 | Heritability of factors of the insulin resistance syndrome in women twins | 1997 | Genetic epidemiology | This study was excluded because it did not provide the exposure on Tg/glucose or Tg/HDL index values. |
| 295 | Aerobic fitness, body mass index, and CVD risk factors among adolescents: the Quebec family study | 2005 | International journal of obesity | This study was excluded because it did not provide the exposure on Tg/glucose or Tg/HDL index values. |
| 296 | Moderate to Vigorous Physical Activity and Sedentary Time and Cardiometabolic Risk Factors in Children and Adolescents | 2012 | Jama-journal of the american medical association | This study was excluded because it did not provide the exposure on Tg/glucose or Tg/HDL index values. |
| 297 | Genetic, dietary, and non-dietary risk factors of obesity among preparatory-year female students at Taibah University, Saudi Arabia | 2017 | Journal of taibah university for science | This study was excluded because it did not provide the exposure on Tg/glucose or Tg/HDL index values. |
| 298 | Quantitative trait linkage analysis of lipid-related traits in familial type 2 diabetes - Evidence for linkage of triglyceride levels to chromosome 19q | 2002 | Diabetes | This study was excluded because it did not provide the exposure on Tg/glucose or Tg/HDL index values. |
| 299 | Genetic and environmental influences on factors associated with cardiovascular disease and the metabolic syndrome | 2009 | Journal of lipid research | This study was excluded because it did not provide the exposure on Tg/glucose or Tg/HDL index values. |
| 300 | Prevalence of Metabolic Syndrome in Middle School Children and Evaluation of Components of Metabolic Syndrome | 2019 | Medical bulletin of sisli etfal hospital | This study was excluded because it did not provide the exposure on Tg/glucose or Tg/HDL index values. |
| 301 | Insulin resistance is a key factor for metabolic and inflammatory biomarkers in children obesity | 2015 | Gastroenterology | This study was excluded because it did not provide the exposure on Tg/glucose or Tg/HDL index values. |
| 302 | Lipid profiles in type 1 diabetic children | 2017 | Pediatric diabetes | This study was excluded because it did not provide the exposure on Tg/glucose or Tg/HDL index values. |
| 303 | Short-term therapy with combination dipeptidyl peptidase-4 inhibitor saxagliptin/metformin extended release (XR) is superior to saxagliptin or metformin XR monotherapy in prediabetic women with polycystic ovary syndrome: a single-blind, randomized, pilot study | 2017 | Fertility and sterility | This study was excluded because it did not provide the exposure on Tg/glucose or Tg/HDL index values. |
| 304 | Metabolic and Addiction Indices in Patients on Opioid Agonist Medication-Assisted Treatment: A Comparison of Buprenorphine and Methadone | 2020 | Scientific reports | This study was excluded because it did not provide the exposure on Tg/glucose or Tg/HDL index values. |
| 305 | Dietary quality indices in relation to cardiometabolic risk among Finnish children aged 6-8years-The PANIC study | 2016 | Nutrition metabolism and cardiovascular diseases | This study was excluded because it did not provide the exposure on Tg/glucose or Tg/HDL index values. |
| 306 | Prevalence and Predictors of Insulin Resistance in Non-Obese Healthy Young Females in Qatar | 2020 | International journal of environmental research and public health | This study was excluded because it did not provide the exposure on Tg/glucose or Tg/HDL index values. |
| 307 | Evaluation of epicardial fat and carotid intima-media thickness in obese children | 2016 | Iranian Journal of Pediatrics | This study was excluded because it did not provide the exposure on Tg/glucose or Tg/HDL index values. |
| 308 | Association of Trp64Arg polymorphism of the beta(3)-adrenergic receptor gene and no association of Gln223Arg polymorphism of the leptin receptor gene in Japanese schoolchildren with obesity | 2000 | International journal of obesity | This study was excluded because it did not provide the exposure on Tg/glucose or Tg/HDL index values. |
| 309 | TG/HDL ratio as a predictor of insulin resistance in U.S. adolescents | 2012 | Diabetes | This study was excluded because it did not provide the exposure on Tg/glucose or Tg/HDL index values. |
| 310 | Isotretinoin does not induce insulin resistance in patients with acne | 2011 | Clinical and experimental dermatology | This study was excluded because it did not provide the exposure on Tg/glucose or Tg/HDL index values. |
| 311 | Low birth weight in combination with catch-up growth predicts the occurrence of the metabolic syndrome in men at late middle age: The Atherosclerosis and Insulin Resistance study | 2004 | Journal of Internal Medicine | This study was excluded because it did not provide the exposure on Tg/glucose or Tg/HDL index values. |
| 312 | Insulin resistance is associated with impaired olfactory function in adult patients with type 1 diabetes: A cross-sectional study | 2020 | Diabetes-metabolism research and reviews | This study was excluded because it did not provide the exposure on Tg/glucose or Tg/HDL index values. |
| 313 | Identification of the G994T polymorphism in exon 9 of plasma platelet-activating factor acetylhydrolase gene as a risk factor for polycystic ovary syndrome | 2010 | Human reproduction | This study was excluded because it did not provide the exposure on Tg/glucose or Tg/HDL index values. |
| 314 | Carotid intima-media thickness in childhood and adolescent obesity relations to abdominal obesity, high triglyceride level and insulin resistance | 2010 | International Journal of Medical Sciences | This study was excluded because it did not provide the exposure on Tg/glucose or Tg/HDL index values. |
| 315 | Serum calcium, magnesium, phosphorous and lipid profile in healthy Iranian premenopausal women | 2011 | Biochemia medica | This study was excluded because it did not provide the exposure on Tg/glucose or Tg/HDL index values. |
| 316 | Metabolites as novel biomarkers for childhood obesity-related traits in Mexican-American children | 2015 | Pediatric obesity | This study was excluded because it did not provide the exposure on Tg/glucose or Tg/HDL index values. |
| 317 | The relationship of serum triglycerides, serum HDL, and obesity to the risk of wheezing in 85,555 adults | 2015 | Respir med | This study was excluded because it did not provide the exposure on Tg/glucose or Tg/HDL index values. |
| 318 | Relationship between gestational diabetes and metabolic syndrome | 2007 | Revista Medica de Chile | This study was excluded because it did not provide the exposure on Tg/glucose or Tg/HDL index values. |
| 319 | Increased Cholesteryl Ester Transfer Protein and Lipoprotein-Associated Phospholipase A2 Activities in Children and Adolescents Presenting High Triglyceride/High-Density Lipoprotein Cholesterol (TG/HDL-C) Ratio | 2021 | Indian Journal of Pediatrics | This study was excluded because it did not provide the exposure on Tg/glucose or Tg/HDL index values. |
| 320 | Insulin resistance and its relationship with overweight in adolescents from a capital city in Northeastern Brazil | 2020 | Adolescencia e Saude | This study was excluded because it did not provide the exposure on Tg/glucose or Tg/HDL index values. |
| 321 | Do post-menarcheal anthropometric measures of body fat better predict late adolescent lipid levels than pre-menarche values? | 2017 | Faseb journal | This study was excluded because it did not provide the exposure on Tg/glucose or Tg/HDL index values. |
| 322 | Comparison of indirect markers of insulin resistance in adult patients with Double Diabetes | 2020 | Bmc endocrine disorders | This study was excluded because it did not provide the exposure on Tg/glucose or Tg/HDL index values. |
| 323 | Obesity onset on early ages is a predictive factor for higher TG/HDL-c values in women with severe obesity and metabolic syndrome | 2020 | Obesity reviews | This study was excluded because it did not provide the exposure on Tg/glucose or Tg/HDL index values. |
| 324 | Triglyceride-To-High-Density-Lipoprotein-Cholesterol Ratio as a Predictor for Metabolic Syndrome According to Obesity Onset in Women With Severe Obesity | 2021 | Metabolism: Clinical and Experimental | This study was excluded because it did not provide the exposure on Tg/glucose or Tg/HDL index values. |
| 325 | Effects of a lifestyle modification program in HIV-infected patients with the metabolic syndrome | 2006 | Aids | This study was excluded because it did not provide the exposure on Tg/glucose or Tg/HDL index values. |
| 326 | Cardiometabolic risk indicators that distinguish adults with psychosis from the general population, by age and gender | 2013 | Plos ONE | This study was excluded because it did not provide the exposure on Tg/glucose or Tg/HDL index values. |
| 327 | Excess serum uric acid is associated with metabolic syndrome in obese adolescent patients | 2020 | Journal of Diabetes and Metabolic Disorders | This study was excluded because it did not provide the exposure on Tg/glucose or Tg/HDL index values. |
| 328 | [Hypertension in the metabolic syndrome among Caribbean non diabetic subjects] | 2020 | Arch mal coeur vaiss | This study was excluded because it did not provide the exposure on Tg/glucose or Tg/HDL index values. |
| 329 | Genetic epidemiology of cardiometabolic risk factors and their clustering patterns in Mexican American children and adolescents: The SAFARI Study | 2013 | Human genetics | This study was excluded because it did not provide the exposure on Tg/glucose or Tg/HDL index values. |
| 330 | Correlation between visceral adipose tissue and biochemical rates related to obesity in newborns | 2014 | Obstetrics and Gynecology | This study was excluded because it did not provide the exposure on Tg/glucose or Tg/HDL index values. |
| 331 | Childhood Muscular Fitness Phenotypes and Adult Metabolic Syndrome | 2016 | Medicine and science in sports and exercise | This study was excluded because it did not provide the exposure on Tg/glucose or Tg/HDL index values. |
| 332 | Estimates of insulin sensitivity and beta-cell function in children and adolescents with and without components of the metabolic syndrome | 2017 | Pediatric endocrinology, diabetes, and metabolism | This study was excluded because it did not provide the exposure on Tg/glucose or Tg/HDL index values. |
| 333 | Estimates of insulin sensitivity and Î²-cell function in children and adolescents with and without components of the metabolic syndrome | 2017 | Pediatric Endocrinology, Diabetes and Metabolism | This study was excluded because it did not provide the exposure on Tg/glucose or Tg/HDL index values. |
| 334 | Longitudinal changes in risk variables underlying metabolic Syndrome X from childhood to young adulthood in female subjects with a history of early menarche: The Bogalusa Heart Study | 2003 | International Journal of Obesity | This study was excluded because it did not provide the exposure on Tg/glucose or Tg/HDL index values. |
| 335 | [Non-linear canonical correlation analysis between anthropometric indicators and multiple metabolic abnormalities] | 2003 | Wei sheng yan jiu | This study was excluded because it did not provide the exposure on Tg/glucose or Tg/HDL index values. |
| 336 | Nutritional Status Influences Oxidative Stress and Insulin Resistance in Preschool Children | 2021 | Metabolic syndrome and related disorders | This study was excluded because it did not provide the exposure on Tg/glucose or Tg/HDL index values. |
| 337 | Insulin resistance in women after pregnancy is associated with changes in the serum nonesterified fatty acid profile | 2014 | Diabetologie und Stoffwechsel | This study was excluded because it did not provide the exposure on Tg/glucose or Tg/HDL index values. |
| 338 | Association of whole-grain and dietary fiber intake with cardiometabolic risk in children and adolescents | 2014 | Nutr health | This study was excluded because it did not provide the exposure on Tg/glucose or Tg/HDL index values. |
| 339 | The Association Of The Metabolic Syndrome With The Presence Of Cardiovascular Episodes In Patients Under Regular Medical Examination | 2021 | Metabolism: Clinical and Experimental | This study was excluded because it did not provide the exposure on Tg/glucose or Tg/HDL index values. |
| 340 | Associations between two single nucleotide polymorphisms of the adiponectin gene, its circulating concentrations and cardiometabolic risk factors in prepubertal children with and without abdominal obesity | 2013 | Journal of endocrinological investigation | This study was excluded because it did not provide the exposure on Tg/glucose or Tg/HDL index values. |
| 341 | The role of haemoglobin A1c in screening obese children and adolescents for glucose intolerance and type 2 diabetes | 2015 | Acta medica portuguesa | This study was excluded because it did not provide the exposure on Tg/glucose or Tg/HDL index values. |
| 342 | Distribution and correlates of non-high-density lipoprotein cholesterol and triglycerides in Lebanese school children | 2016 | Journal of clinical lipidology | This study was excluded because it did not provide the exposure on Tg/glucose or Tg/HDL index values. |
| 343 | Comparison of differents procedures of evaluation of insulin resistance in children and adolescents in a mexican population | 2011 | Journal of Diabetes | This study was excluded because it did not provide the exposure on Tg/glucose or Tg/HDL index values. |
| 344 | Advanced glycation end products and their receptors did not show any association with body mass parameters in metabolically healthy adolescents | 2018 | Acta Paediatrica, International Journal of Paediatrics | This study was excluded because it did not provide the exposure on Tg/glucose or Tg/HDL index values. |
| 345 | Diagnostic accuracy of triglyceride/glucose and triglyceride/HDL index as predictors for insulin resistance in children with and without obesity | 2019 | Diabetes metab syndr | Included |
| 346 | Cardiovascular risk factors associated with the metabolic syndrome in obese adolescents | 2011 | Journal of Diabetes | This study was excluded because it did not provide the exposure on Tg/glucose or Tg/HDL index values. |
| 347 | Insulin resistance--a risk factor for coronary heart disease? | 1983 | Scandinavian journal of clinical and laboratory investigation | This study was excluded because it did not provide the exposure on Tg/glucose or Tg/HDL index values. |
| 348 | Insulin resistance - a risk factor for coronary heart disease?? | 1983 | Scandinavian Journal of Clinical and Laboratory Investigation | This study was excluded because it did not provide the exposure on Tg/glucose or Tg/HDL index values. |
| 349 | Association study of candidate genes with obesity and metabolic traits in antipsychotic-treated patients with first-episode psychosis over a 2-year period | 2020 | Journal of psychopharmacology | This study was excluded because it did not provide the exposure on Tg/glucose or Tg/HDL index values. |
| 350 | The triglyceride-glucose index, an insulin resistance marker in newborns? | 2018 | European Journal of Pediatrics | This study was excluded because it did not provide the exposure on Tg/glucose or Tg/HDL index values. |
| 351 | Cord‑blood lipoproteins, homocysteine, insulin sensitivity/resistance marker profile, and concurrence of dysglycaemia and dyslipaemia in full‑term neonates of the Mérida Study. | 2013 | European journal of pediatrics | This study was excluded because it did not provide the exposure on Tg/glucose or Tg/HDL index values. |
| 352 | Cord‑blood lipoproteins, homocysteine, insulin sensitivity/resistance marker profile, and concurrence of dysglycaemia and dyslipaemia in full‑term neonates of the Mérida Study. | 2013 | European Journal of Pediatrics | This study was excluded because it did not provide the exposure on Tg/glucose or Tg/HDL index values. |
| 353 | Low birth weight for gestational age associates with reduced glucose concentrations at birth, infancy and childhood | 2007 | Hormone research | This study was excluded because it did not provide the exposure on Tg/glucose or Tg/HDL index values. |
| 354 | Blood pressure, dyslipidemia and inflammatory factors are related to body mass index in scholar adolescents | 2017 | Archives of Medical Science | This study was excluded because it did not provide the exposure on Tg/glucose or Tg/HDL index values. |
| 355 | The triglyceride-to-HDL cholesterol ratio: Association with insulin resistance in obese youths of different ethnic backgrounds | 2011 | Diabetes care | Included |
| 356 | The triglyceride-HDL cholesterol ratio: A potentially useful marker of insulin resistance in obese children and adolescents of different racial/ethnic background | 2011 | Diabetes | This study was excluded because it did not provide the exposure on Tg/glucose or Tg/HDL index values. |
| 357 | Adolescent and adult African Americans have similar metabolic dyslipidemia | 2015 | Journal of clinical lipidology | This study was excluded because it did not provide the exposure on Tg/glucose or Tg/HDL index values. |
| 358 | RBP4 and subclinical inflammation in childhood obesity | 2010 | Obesity reviews | This study was excluded because it did not provide the exposure on Tg/glucose or Tg/HDL index values. |
| 359 | Cardiometabolic outcomes in children and adolescents with West syndrome | 2021 | Bmc pediatrics | This study was excluded because it did not provide the exposure on Tg/glucose or Tg/HDL index values. |
| 360 | Family history of diabetes, autoimmunity, and risk factors for cardiovascular disease among children with diabetes in the SEARCH for Diabetes in Youth Study | 2007 | Pediatric diabetes | This study was excluded because it did not provide the exposure on Tg/glucose or Tg/HDL index values. |
| 361 | Infection with Helicobacter pylori, coronary heart disease, cardiovascular risk factors, and systemic inflammation: The Third National Health and Nutrition Examination Survey | 2004 | Journal of the national medical association | This study was excluded because it did not provide the exposure on Tg/glucose or Tg/HDL index values. |
| 362 | Insulin resistance and triglycerides | 2009 | Journal of Investigative Medicine | This study was excluded because it did not provide the exposure on Tg/glucose or Tg/HDL index values. |
| 363 | Ethnic differences in serum lipoproteins and their determinants in South African women | 2010 | Metabolism-clinical and experimental | This study was excluded because it did not provide the exposure on Tg/glucose or Tg/HDL index values. |
| 364 | Visceral adipose tissue and metabolic complications of obesity are reduced in Prader-Willi syndrome female adults: Evidence for novel influences on body fat distribution | 2001 | Journal of clinical endocrinology & metabolism | This study was excluded because it did not provide the exposure on Tg/glucose or Tg/HDL index values. |
| 365 | Nine-year longitudinal study of cardiovascular risk factors in spanish children and adolescents with type 1 diabetes | 2015 | Anales de pediatria | This study was excluded because it did not provide the exposure on Tg/glucose or Tg/HDL index values. |
| 366 | Early adiposity rebound is associated with metabolic risk in 7-year-old children | 2014 | International Journal of Obesity | This study was excluded because it did not provide the exposure on Tg/glucose or Tg/HDL index values. |
| 367 | Early adiposity rebound is associated with increased metabolic risk at age 7y in chilean children | 2013 | Annals of Nutrition and Metabolism | This study was excluded because it did not provide the exposure on Tg/glucose or Tg/HDL index values. |
| 368 | Early adiposity rebound and metabolic risk at 7 years | 2014 | Pediatric research | This study was excluded because it did not provide the exposure on Tg/glucose or Tg/HDL index values. |
| 369 | Intra-abdominal and subcutaneous abdominal fat as predictors of cardiometabolic risk in a sample of Mexican children | 2017 | European Journal of Clinical Nutrition | This study was excluded because it did not provide the exposure on Tg/glucose or Tg/HDL index values. |
| 370 | Effect of malnutrition during the first year of life on adult plasma insulin and glucose tolerance | 2003 | Metabolism: Clinical and Experimental | This study was excluded because it did not provide the exposure on Tg/glucose or Tg/HDL index values. |
| 371 | Myokine-adipokine cross-talk: Potential mechanisms for the association between plasma irisin and adipokines and cardiometabolic risk factors in Mexican children with obesity and the metabolic syndrome | 2019 | Diabetology and Metabolic Syndrome | This study was excluded because it did not provide the exposure on Tg/glucose or Tg/HDL index values. |
| 372 | Serum transaminases concentrations in obese children and adolescents | 2009 | Journal of physiology and biochemistry | This study was excluded because it did not provide the exposure on Tg/glucose or Tg/HDL index values. |
| 373 | Effectiveness, safety and metabolic effects of carbohydrate restriction in the management of obesity in adolescents after 1 year follow-up | 2017 | Hormone Research in Paediatrics | This study was excluded because it did not provide the exposure on Tg/glucose or Tg/HDL index values. |
| 374 | Influence of neonatal anthropometry on the comorbidities of the obese patient | 2019 | Anales de Pediatria | This study was excluded because it did not provide the exposure on Tg/glucose or Tg/HDL index values. |
| 375 | C-Peptide Is a Sensitive Indicator for the Diagnosis of Metabolic Syndrome in Subjects from Central Mexico | 2016 | Metabolic syndrome and related disorders | This study was excluded because it did not provide the exposure on Tg/glucose or Tg/HDL index values. |
| 376 | TG/HDL index in adolescents of Salta, Argentina | 2014 | Revista de la Federacion Argentina de Cardiologia | This study was excluded because it did not provide the exposure on Tg/glucose or Tg/HDL index values. |
| 377 | Assessment of Serum Vitamin D and Irisin Levels in Obese Patients | 2018 | Clinical laboratory | This study was excluded because it did not provide the exposure on Tg/glucose or Tg/HDL index values. |
| 378 | The intravenous insulin tolerance test is an accurate method for screening a general population for insulin resistance and related abnormalities | 1999 | Journal of Endocrinological Investigation | This study was excluded because it did not provide the exposure on Tg/glucose or Tg/HDL index values. |
| 379 | Chronic intake of energy drinks and their sugar free substitution similarly promotes metabolic syndrome | 2021 | Nutrients | This study was excluded because it did not provide the exposure on Tg/glucose or Tg/HDL index values. |
| 380 | Insulin resistance better predicts increased arterial stiffness in obese children than dyslipidemia or the degree of obesity | 2017 | Hormone Research in Paediatrics | This study was excluded because it did not provide the exposure on Tg/glucose or Tg/HDL index values. |
| 381 | ENHO, RXRA, and LXRA polymorphisms and dyslipidaemia, related comorbidities and survival in haemodialysis patients | 2018 | Bmc medical genetics | This study was excluded because it did not provide the exposure on Tg/glucose or Tg/HDL index values. |
| 382 | Metabolic syndrome, an important issue in patients with congenital generalized lipodystrophy | 2009 | European heart journal | This study was excluded because it did not provide the exposure on Tg/glucose or Tg/HDL index values. |
| 383 | Reduction in d-lactate correlates with reductions in liver fat and de novo lipogenesis (DNL) after fructose restriction in obese children and adolescents: A new link between fructose and non-alcoholic fatty liver disease (NAFLD)? | 2018 | Hepatology | This study was excluded because it did not provide the exposure on Tg/glucose or Tg/HDL index values. |
| 384 | Adiponectin paradox in lean versus obese adolescents: Highest serum levels are found in lean adolescents with insulin resistance | 2021 | Atherosclerosis | This study was excluded because it did not provide the exposure on Tg/glucose or Tg/HDL index values. |
| 385 | Short-term isocaloric fructose restriction lowers apoC-III levels and yields less atherogenic lipoprotein profiles in children with obesity and metabolic syndrome | 2016 | Atherosclerosis | This study was excluded because it did not provide the exposure on Tg/glucose or Tg/HDL index values. |
| 386 | Ethinylestradio-Chlormadinone Acetate Combination for the Treatment of Hirsutism and Hormonal Alterations of Normal-Weight Women With Polycystic Ovary Syndrome: Evaluation of the Metabolic Impact | 2010 | Reproductive sciences | This study was excluded because it did not provide the exposure on Tg/glucose or Tg/HDL index values. |
| 387 | Prevalence of carbohydrate metabolism disturbances in a population of children and adolescents with severe obesity | 2010 | Endocrinologia y Nutricion | This study was excluded because it did not provide the exposure on Tg/glucose or Tg/HDL index values. |
| 388 | Prevalence of metabolic syndrome in a population of obese children and adolescents | 2012 | Endocrinologia y Nutricion | This study was excluded because it did not provide the exposure on Tg/glucose or Tg/HDL index values. |
| 389 | Early Evidence of Increased Risk for Metabolic Syndrome in Young Men With Latent Obstructive Sleep Apnea | 2010 | Metabolic syndrome and related disorders | This study was excluded because it did not provide the exposure on Tg/glucose or Tg/HDL index values. |
| 390 | Prenatal exposure to persistent organic pollutants and markers of obesity and cardiometabolic risk in Spanish adolescents | 2021 | Environment international | This study was excluded because it did not provide the exposure on Tg/glucose or Tg/HDL index values. |
| 391 | Insulin resistance and clustering of cardiometabolic risk factors in Brazilian adolescents: Effect of body weight | 2008 | Circulation | This study was excluded because it did not provide the exposure on Tg/glucose or Tg/HDL index values. |
| 392 | Advanced glycation endproduct ligand and it's membrane-bound receptor (RAGE) are inversely associated in obese adolescents | 2015 | Endocrine reviews | This study was excluded because it did not provide the exposure on Tg/glucose or Tg/HDL index values. |
| 393 | Fasting serum IGFBP-1 with fasting serum insulin is a more sensitive marker of insulin resistance and hypertriglyceridemia in children | 2010 | Hormone Research in Paediatrics | This study was excluded because it did not provide the exposure on Tg/glucose or Tg/HDL index values. |
| 394 | Prevalence of elevated alanine aminotransferase levels in obese children and its association with their age, gender and metabolic profile | 2010 | Hormone Research in Paediatrics | This study was excluded because it did not provide the exposure on Tg/glucose or Tg/HDL index values. |
| 395 | A confirmatory factor analysis of the metabolic syndrome in adolescents: an examination of sex and racial/ethnic differences | 2012 | Cardiovascular diabetology | This study was excluded because it did not provide the exposure on Tg/glucose or Tg/HDL index values. |
| 396 | Lean Adolescents with Insulin Resistance Display Higher Angiopoietin-like Protein 3, apoC-III and Triglyceride Rich Lipoprotein Dyslipidemia | 2020 | American heart journal | This study was excluded because it did not provide the exposure on Tg/glucose or Tg/HDL index values. |
| 397 | A randomized, placebo-controlled, double-blind study on the effects of (-)- epicatechin on the triglyceride/HDLc ratio and cardiometabolic profile of subjects with hypertriglyceridemia: Unique in vitro effects | 2016 | International journal of cardiology | This study was excluded because it did not provide the exposure on Tg/glucose or Tg/HDL index values. |
| 398 | A randomized, placebo-controlled, double-blind study on the effects of (âˆ’)-epicatechin on the triglyceride/HDLc ratio and cardiometabolic profile of subjects with hypertriglyceridemia: Unique in vitro effects | 2016 | International Journal of Cardiology | This study was excluded because it did not provide the exposure on Tg/glucose or Tg/HDL index values. |
| 399 | [Prevalence of metabolic syndrome in children with and without obesity] | 2016 | Med clin (barc) | This study was excluded because it did not provide the exposure on Tg/glucose or Tg/HDL index values. |
| 400 | Mediating effects of motor performance, cardiorespiratory fitness, physical activity, and sedentary behaviour on the associations of adiposity and other cardiometabolic risk factors with academic achievement in children | 2018 | Journal of sports sciences | This study was excluded because it did not provide the exposure on Tg/glucose or Tg/HDL index values. |
| 401 | Associations of Genetic Susceptibility to Alzheimer's Disease with Adiposity and Cardiometabolic Risk Factors among Children in a 2-Year Follow-up Study | 2018 | Journal of Alzheimer's Disease | This study was excluded because it did not provide the exposure on Tg/glucose or Tg/HDL index values. |
| 402 | Are the ratio triglycerides to high density lipoprotein cholesterol (TG/HDLC) as indicator for insulin resistance (IR) affected by age? The PEP family heart study | 2008 | European heart journal | This study was excluded because it did not provide the exposure on Tg/glucose or Tg/HDL index values. |
| 403 | Associations of skinfold thickness with cardiovascular risk factors in 7076 children and adolescents participating in the PEP Family Heart Study | 2018 | European heart journal | This study was excluded because it did not provide the exposure on Tg/glucose or Tg/HDL index values. |
| 404 | Metabolic syndrome in Egyptian adolescents | 2011 | Hormone Research in Paediatrics | This study was excluded because it did not provide the exposure on Tg/glucose or Tg/HDL index values. |
| 405 | ASK1 (MAP3K5) is transcriptionally upregulated by E2F1 in adipose tissue in obesity, molecularly defining a human dys-metabolic obese phenotype | 2017 | Molecular metabolism | This study was excluded because it did not provide the exposure on Tg/glucose or Tg/HDL index values. |
| 406 | Association between C-reactive protein and alanine aminotransferase as cardiovascular risk factors in adolescents | 2009 | Journal of Diabetes | This study was excluded because it did not provide the exposure on Tg/glucose or Tg/HDL index values. |
| 407 | Metabolic syndrome and leptin concentrations in obese children | 2009 | Indian j pediatr | This study was excluded because it did not provide the exposure on Tg/glucose or Tg/HDL index values. |
| 408 | The cutoff values of indirect indices for measuring insulin resistance in Korean children and adolescents | 2017 | International Journal of Pediatric Endocrinology | This study was excluded because it did not provide the exposure on Tg/glucose or Tg/HDL index values. |
| 409 | Use of markers of dyslipidemia to identify overweight youth with insulin resistance | 2006 | Pediatric diabetes | Included |
| 410 | Weekly variation in markers of cardiometabolic health - the possible effect of weekend behavior - a cross-sectional study | 2020 | Bmc cardiovascular disorders | This study was excluded because it did not provide the exposure on Tg/glucose or Tg/HDL index values. |
| 411 | Higher serum uric acid is associated with arterial stiffening and cardiac hypertrophy in childhood obesity | 2014 | Circulation | This study was excluded because it did not provide the exposure on Tg/glucose or Tg/HDL index values. |
| 412 | Higher triglyceride to high density lipoprotein cholesterol ratio is associated with arterial-ventricular stiffening in childhood obesity | 2017 | Circulation | This study was excluded because it did not provide the exposure on Tg/glucose or Tg/HDL index values. |
| 413 | Importance of cystatin C and uric acid levels in the association of cardiometabolic risk factors in Japanese junior high school students | 2017 | J cardiol | This study was excluded because it did not provide the exposure on Tg/glucose or Tg/HDL index values. |
| 414 | Lipoprotein subspecies predict arterial stiffness | 2016 | Circulation | This study was excluded because it did not provide the exposure on Tg/glucose or Tg/HDL index values. |
| 415 | Prevalence of metabolic syndrome and insulin resistance among aged 3-9 children | 2014 | Hormone Research in Paediatrics | This study was excluded because it did not provide the exposure on Tg/glucose or Tg/HDL index values. |
| 416 | Neck circumference as a novel parameter to determine non-alcoholic fatty liver disease in obese children | 2011 | Hormone Research in Paediatrics | This study was excluded because it did not provide the exposure on Tg/glucose or Tg/HDL index values. |
| 417 | Prevalence of metabolic syndrome and insulin resistance among aged children 3 to 9 | 2011 | Obesity reviews | This study was excluded because it did not provide the exposure on Tg/glucose or Tg/HDL index values. |
| 418 | Growth in total height and its components and cardiometabolic health in childhood | 2016 | Plos ONE | This study was excluded because it did not provide the exposure on Tg/glucose or Tg/HDL index values. |
| 419 | Moderate alcohol consumption is associated with a decreased prevalence of some but not all cardio-metabolic risk factors in young adults | 2016 | Circulation | This study was excluded because it did not provide the exposure on Tg/glucose or Tg/HDL index values. |
| 420 | Cardiometabolic burden in adolescents is associated with DNA methylation in genes related to cardiovascular disease risk | 2017 | Circulation | This study was excluded because it did not provide the exposure on Tg/glucose or Tg/HDL index values. |
| 421 | Cardiometabolic risks during anabolic hormone supplementation in older men | 2013 | Obesity | This study was excluded because it did not provide the exposure on Tg/glucose or Tg/HDL index values. |
| 422 | The role of prenatal hyperandrogenism on lipid metabolism during adult life in a rat model | 2012 | Medicina-buenos aires | This study was excluded because it did not provide the exposure on Tg/glucose or Tg/HDL index values. |
| 423 | Comparison of Cardiovascular and Metabolic Risk Factors in Professional Baseball Players Versus Professional Football Players | 2010 | American journal of cardiology | This study was excluded because it did not provide the exposure on Tg/glucose or Tg/HDL index values. |
| 424 | Metabolic determinants are much more important than genetic polymorphisms in determining the PAI-1 activity and antigen plasma concentrations - A family study with part of the Stanislas Cohort | 1998 | Arteriosclerosis thrombosis and vascular biology | This study was excluded because it did not provide the exposure on Tg/glucose or Tg/HDL index values. |
| 425 | Metabolic determinants are much more important than genetic polymorphisms in determining the PAI-1 activity and antigen plasma concentrations: A family study with part of the stanislas cohort | 1998 | Arteriosclerosis, Thrombosis, and Vascular Biology | This study was excluded because it did not provide the exposure on Tg/glucose or Tg/HDL index values. |
| 426 | Parental brevity linked to cardiometabolic risk in diabetic descendants | 2014 | Journal of diabetes and its complications | This study was excluded because it did not provide the exposure on Tg/glucose or Tg/HDL index values. |
| 427 | Strength training improves insulin sensitivity and plasma lipid levels without altering body composition in overweight and obese subjects | 2011 | Endocrinologia y nutricion : organo de la Sociedad Espanola de Endocrinologia y Nutricion | This study was excluded because it did not provide the exposure on Tg/glucose or Tg/HDL index values. |
| 428 | PEDIATRIC VISCERAL ADIPOSITY INDEX ADAPTATION CORRELATES with HOMA-IR, MATSUDA, and TRANSAMINASES | 2018 | Endocrine practice | This study was excluded because it did not provide the exposure on Tg/glucose or Tg/HDL index values. |
| 429 | Association of metabolic syndrome with reduced central serotonergic activity | 2011 | Metabolic brain disease | This study was excluded because it did not provide the exposure on Tg/glucose or Tg/HDL index values. |
| 430 | Metabolic Syndrome in the Military Health System Based on Electronic Health Data, 2009-2012 | 2015 | Military medicine | This study was excluded because it did not provide the exposure on Tg/glucose or Tg/HDL index values. |
| 431 | Relationship of cardiometabolic risk biomarkers with DXA and pQCT bone health outcomes in young girls | 2019 | Bone | This study was excluded because it did not provide the exposure on Tg/glucose or Tg/HDL index values. |
| 432 | Weight change and changes in the metabolic syndrome as the French population moves towards overweight: The DESIR Cohort | 2006 | International journal of epidemiology | This study was excluded because it did not provide the exposure on Tg/glucose or Tg/HDL index values. |
| 433 | Association between triglyceride-to-HDL-C ratio and insulin-resistance among argentine indian and mixed population school children | 2013 | Diabetes | This study was excluded because it did not provide the exposure on Tg/glucose or Tg/HDL index values. |
| 434 | Association between school children's overweight and maternal obesity and perception of their children's weight status | 2008 | Journal of Pediatric Endocrinology and Metabolism | This study was excluded because it did not provide the exposure on Tg/glucose or Tg/HDL index values. |
| 435 | Comparison of different markers of insulin resistance in indigenous Argentinian school children | 2014 | Pediatric diabetes | This study was excluded because it did not provide the exposure on Tg/glucose or Tg/HDL index values. |
| 436 | Triglyceride to HDL-C ratio levels in indigenous Argentinean children living at different altitudes | 2017 | Pediatric diabetes | This study was excluded because it did not provide the exposure on Tg/glucose or Tg/HDL index values. |
| 437 | Association between triglyceride to HDL-C ratio and insulin resistance in indigenous Argentinean children | 2015 | Pediatric diabetes | This study was excluded because it did not provide the exposure on Tg/glucose or Tg/HDL index values. |
| 438 | Association between glomerular filtration rate and body fat among normal adolescent boys | 2010 | Diabetes | This study was excluded because it did not provide the exposure on Tg/glucose or Tg/HDL index values. |
| 439 | Low physical activity level and short sleep duration are associated with an increased cardio-metabolic risk profile: A longitudinal study in 8-11 year old Danish children | 2014 | Plos ONE | This study was excluded because it did not provide the exposure on Tg/glucose or Tg/HDL index values. |
| 440 | [Reduction of abdominal obesity and cardiometabolic health risks in obese adolescents in response to a short-term spa weight management program] | 2010 | Casopã­s lã©kaå™Å¯ Äeskã½ch | This study was excluded because it did not provide the exposure on Tg/glucose or Tg/HDL index values. |
| 441 | Relationship between TG/HDL-C ratio and metabolic syndrome risk factors with chronic kidney disease in healthy adult population | 2015 | Clinical nutrition | This study was excluded because it did not provide the exposure on Tg/glucose or Tg/HDL index values. |
| 442 | Monophasic glucose response curve is associated with increased c-reactive protein in obese adolescents | 2021 | Diabetes | This study was excluded because it did not provide the exposure on Tg/glucose or Tg/HDL index values. |
| 443 | Oral glucose tolerance response curve predicts disposition index but not other cardiometabolic risk factors in healthy adolescents | 2021 | Journal of Pediatric Endocrinology and Metabolism | This study was excluded because it did not provide the exposure on Tg/glucose or Tg/HDL index values. |
| 444 | Cardio-metabolic risk profiling for multiethnic early-onset type 2 diabetes: A case-control study | 2018 | Journal of Diabetes Investigation | This study was excluded because it did not provide the exposure on Tg/glucose or Tg/HDL index values. |
| 445 | The prevalence of polycystic ovary syndrome (PCOS) in type 2 diabetic women in reprodutive ages | 2006 | Iranian Journal of Diabetes and Lipid Disorders | This study was excluded because it did not provide the exposure on Tg/glucose or Tg/HDL index values. |
| 446 | Improvement of Insulin Resistance and Reduction of Cardiovascular Risk Among Obese Patients with Type 2 Diabetes with the Duodenojejunal Bypass Liner | 2011 | Obesity surgery | This study was excluded because it did not provide the exposure on Tg/glucose or Tg/HDL index values. |
| 447 | Physical activity is related to insulin sensitivity and the metabolic syndrome in adolescents | 2007 | Circulation | This study was excluded because it did not provide the exposure on Tg/glucose or Tg/HDL index values. |
| 448 | Are there socioeconomic inequalities in cardiovascular risk factors in childhood, and are they mediated by adiposity? Findings from a prospective cohort study | 2010 | International journal of obesity | This study was excluded because it did not provide the exposure on Tg/glucose or Tg/HDL index values. |
| 449 | The association between maternal 25-hydroxyvitamin D concentration during gestation and early childhood cardiometabolic outcomes: Is there interaction with pre-pregnancy BMI? | 2015 | Plos ONE | This study was excluded because it did not provide the exposure on Tg/glucose or Tg/HDL index values. |
| 450 | Insulin, proinsulin and insulin resistance status in relation to lipid profiles among school children in Taiwan - The Taipei Children Heart Study | 2003 | Clinical biochemistry | This study was excluded because it did not provide the exposure on Tg/glucose or Tg/HDL index values. |
| 451 | Abnormal liver function test results are related to metabolic syndrome and BMI in Taiwanese adults without chronic hepatitis B or C | 2009 | International journal of obesity | This study was excluded because it did not provide the exposure on Tg/glucose or Tg/HDL index values. |
| 452 | Supplementary therapy with omega‑3 polyunsaturated fatty acids improves metabolic indices in children with non‑alcoholic fatty liver disease: a meta-analysis | 2020 | Chinese general practice | This study was excluded because it did not provide the exposure on Tg/glucose or Tg/HDL index values. |
| 453 | A randomized, 12-week study of the effects of extended-release paliperidone (paliperidone ER) and olanzapine on metabolic profile, weight, insulin resistance, and β-cell function in schizophrenic patients | 2013 | Psychopharmacology | This study was excluded because it did not provide the exposure on Tg/glucose or Tg/HDL index values. |
| 454 | Relationship between triglyceride glucose index and the incidence of non-alcoholic fatty liver disease in the elderly: a retrospective cohort study in China | 2020 | Bmj open | This study was excluded because it did not provide the exposure on Tg/glucose or Tg/HDL index values. |
| 455 | No difference in serum adiponectin, resistin, TNFR1 and TNFR2 in obese adolescents with or without insulin resistance | 2013 | Hormone Research in Paediatrics | This study was excluded because it did not provide the exposure on Tg/glucose or Tg/HDL index values. |
| 456 | Effect of weight loss induced by 6-month lifestyle intervention on adipokines in obese adolescents | 2011 | Annals of Nutrition and Metabolism | This study was excluded because it did not provide the exposure on Tg/glucose or Tg/HDL index values. |
| 457 | Weight loss induced by 6-month lifestyle intervention improves early endothelial activation and fibrinolysis in obese adolescents | 2011 | Child care health and development | This study was excluded because it did not provide the exposure on Tg/glucose or Tg/HDL index values. |
| 458 | Sensitivity, specificity, and predictive values of pediatric metabolic syndrome components in relation to adult metabolic syndrome: The princeton LRC follow-up study | 2008 | Journal of pediatrics | This study was excluded because it did not provide the exposure on Tg/glucose or Tg/HDL index values. |
| 459 | Evaluation of two single-factor models of metabolic syndrome: a confirmatory factor analysis for an adult population in Beijing | 2013 | Lipids in health and disease | This study was excluded because it did not provide the exposure on Tg/glucose or Tg/HDL index values. |
| 460 | Associations between Sugar Intake from Different Food Sources and Adiposity or Cardio-Metabolic Risk in Childhood and Adolescence: The Korean Child-Adolescent Cohort Study | 2016 | Nutrients | This study was excluded because it did not provide the exposure on Tg/glucose or Tg/HDL index values. |
| 461 | Sleep Duration Predicts Cardiometabolic Risk in Obese Adolescents | 2014 | Journal of pediatrics | This study was excluded because it did not provide the exposure on Tg/glucose or Tg/HDL index values. |
| 462 | Proton pump inhibitors as risk factor for metabolic syndrome and hepatic steatosis in coeliac disease patients on gluten-free diet | 2018 | Journal of gastroenterology | This study was excluded because it did not provide the exposure on Tg/glucose or Tg/HDL index values. |
| 463 | Prevalence of the metabolic syndrome and its component abnormalities among school age Pakistani children | 2018 | J ayub med coll abbottabad | This study was excluded because it did not provide the exposure on Tg/glucose or Tg/HDL index values. |
| 464 | Role of social network behavior and medical issues faced by hemophilic patients: A cross sectional study of children hospital multan city, pakistan | 2021 | Rawal medical journal | This study was excluded because it did not provide the exposure on Tg/glucose or Tg/HDL index values. |
| 465 | Metabolomics of Prader Willi syndrome: Macronutrient regulation of incretin secretion and lipid metabolism | 2017 | Hormone Research in Paediatrics | This study was excluded because it did not provide the exposure on Tg/glucose or Tg/HDL index values. |
| 466 | Hormonal and metabolic effects of carbohydrate restriction in children with Prader-Willi syndrome | 2019 | Clinical endocrinology | This study was excluded because it did not provide the exposure on Tg/glucose or Tg/HDL index values. |
| 467 | Triglyceride to HDL-C Ratio is Associated with Insulin Resistance in Overweight and Obese Children | 2017 | Scientific reports | This study was excluded because it did not provide the exposure on Tg/glucose or Tg/HDL index values. |
| 468 | Impact of lifestyle Intervention on branched-chain amino acid catabolism and insulin sensitivity in adolescents with obesity | 2021 | Endocrinology, Diabetes and Metabolism | This study was excluded because it did not provide the exposure on Tg/glucose or Tg/HDL index values. |
| 469 | Prevalence of the metabolic syndrome among a racially/ethnically diverse group of U.S. eighth-grade adolescents and associations with fasting insulin and homeostasis model assessment of insulin resistance levels | 2008 | Diabetes care | This study was excluded because it did not provide the exposure on Tg/glucose or Tg/HDL index values. |
| 470 | The effect of rosiglitazone on plasma adiponectin and resistin levels in obese PCO woman--preliminary report | 2007 | Przeglaì§d lekarski | This study was excluded because it did not provide the exposure on Tg/glucose or Tg/HDL index values. |
| 471 | A case-control observational study of insulin resistance and metabolic syndrome among the four phenotypes of polycystic ovary syndrome based on Rotterdam criteria | 2015 | Reproductive health | This study was excluded because it did not provide the exposure on Tg/glucose or Tg/HDL index values. |
| 472 | A case-control observational study of insulin resistance and metabolic syndrome among the four phenotypes of polycystic ovary syndrome based on Rotterdam criteria Female Fertility | 2015 | Reproductive health | This study was excluded because it did not provide the exposure on Tg/glucose or Tg/HDL index values. |
| 473 | Association of circulating irisin levels with metabolic and metabolite profiles of Korean adolescents | 2017 | Metabolism: Clinical and Experimental | This study was excluded because it did not provide the exposure on Tg/glucose or Tg/HDL index values. |
| 474 | Association of circulating irisin levels with metabolic and metabolite profiles of Korean adolescents Metabolism Clinical and Experimental | 2017 | Metabolism-clinical and experimental | This study was excluded because it did not provide the exposure on Tg/glucose or Tg/HDL index values. |
| 475 | Phenotypic Characterization of Polycystic Ovary Syndrome in Adolescents Based on Menstrual Irregularity | 2015 | Hormone research in paediatrics | This study was excluded because it did not provide the exposure on Tg/glucose or Tg/HDL index values. |
| 476 | Therapy resistant diabetes mellitus and lipodystrophy: leptin therapy leads to improvement | 2013 | Nederlands tijdschrift voor geneeskunde | This study was excluded because it did not provide the exposure on Tg/glucose or Tg/HDL index values. |
| 477 | In brief: Metabolic syndrome: watch out for teens! | 2019 | Revue Francophone des Laboratoires | This study was excluded because it did not provide the exposure on Tg/glucose or Tg/HDL index values. |
| 478 | The Effect of 4-Week Health Promotion Summer Camp on the Metabolic Syndrome and Insulin Resistance among Obese Elementary Students | 2021 | Journal of the Korean Applied Science and Technology | This study was excluded because it did not provide the exposure on Tg/glucose or Tg/HDL index values. |
| 479 | High triglyceride, low HDL-cholesterol and risk of coronary heart disease | 1996 | Ugeskrift for laeger | This study was excluded because it did not provide the exposure on Tg/glucose or Tg/HDL index values. |
| 480 | The metabolic syndrome among Danish seafarers: a follow-up study | 2016 | International maritime health | This study was excluded because it did not provide the exposure on Tg/glucose or Tg/HDL index values. |
| 481 | Amount of hepatic fat predicts cardiovascular risk independent of insulin resistance among Hispanic-American adolescents | 2015 | Lipids in Health and Disease | This study was excluded because it did not provide the exposure on Tg/glucose or Tg/HDL index values. |
| 482 | Prevalence of metabolic syndrome in Tunisian overweight and obese children | 2015 | Tunis med | This study was excluded because it did not provide the exposure on Tg/glucose or Tg/HDL index values. |
| 483 | Development of age-specific adolescent metabolic syndrome criteria that are linked to the adult treatment panel III and international diabetes federation criteria | 2007 | Journal of the american college of cardiology | This study was excluded because it did not provide the exposure on Tg/glucose or Tg/HDL index values. |
| 484 | Lipid regulation in lipodystrophy versus the obesity-associated metabolic syndrome: The dissociation of HDL-C and triglycerides | 2014 | Journal of Clinical Endocrinology and Metabolism | This study was excluded because it did not provide the exposure on Tg/glucose or Tg/HDL index values. |
| 485 | Novel indexes for diagnosing metabolic syndrome in apparently healthy Gujarati Asian Indians: a cross-sectional study | 2016 | Qjm-an international journal of medicine | This study was excluded because it did not provide the exposure on Tg/glucose or Tg/HDL index values. |
| 486 | Cardiometabolic parameters are associated with interleukin-6 levels in healthy 4-yearold children | 2018 | Endocrine reviews | This study was excluded because it did not provide the exposure on Tg/glucose or Tg/HDL index values. |
| 487 | Childhood metabolic syndrome, inflammation and carotid intima-media thickness. The Aboriginal Birth Cohort Study | 2018 | Int j cardiol | This study was excluded because it did not provide the exposure on Tg/glucose or Tg/HDL index values. |
| 488 | Endothelial dysfunction occurs independently of adipose tissue inflammation and insulin resistance in ovariectomized Yucatan miniature-swine | 2018 | Adipocyte | This study was excluded because it did not provide the exposure on Tg/glucose or Tg/HDL index values. |
| 489 | Triglyceride-to-high density lipoprotein cholesterol ratio and triglyceride-glucose index in the perinatal period of neonates | 2021 | Journal of Maternal-Fetal and Neonatal Medicine | This study was excluded because it did not provide the exposure on Tg/glucose or Tg/HDL index values. |
| 490 | Alternative waist-to-height ratios associated with risk biomarkers in youth with diabetes: comparative models in the SEARCH for Diabetes in Youth Study | 2021 | Int j obes (lond) | This study was excluded because it did not provide the exposure on Tg/glucose or Tg/HDL index values. |
| 491 | Insulin resistance and elevated levels of tissue plasminogen activator in first-degree relatives of South Asian patients with ischemic cerebrovascular disease | 2001 | Stroke | This study was excluded because it did not provide the exposure on Tg/glucose or Tg/HDL index values. |
| 492 | Tracking and determinants of LDL particle size in healthy children from 7 to 11 years of age: the STRIP Study | 2009 | European journal of pediatrics | This study was excluded because it did not provide the exposure on Tg/glucose or Tg/HDL index values. |
| 493 | In search of measures to improve the detection of increased cardiometabolic risk in children using second-generation antipsychotic medications | 2021 | Nordic Journal of Psychiatry | This study was excluded because it did not provide the exposure on Tg/glucose or Tg/HDL index values. |
| 494 | Triglycerides/glucose index is a useful surrogate marker of insulin resistance among adolescents | 2017 | International Journal of Obesity | Included |
| 495 | Triglycerides/glucose index is a useful surrogate marker of insulin resistance in adolescents | 2016 | Diabetes | This study was excluded because it did not provide the exposure on Tg/glucose or Tg/HDL index values. |
| 496 | The Association of Insulin Resistance, Adipocytokine, Cardiovascular Fitness and Heart Rate Recovery after Exercise in Adolescent | 2012 | The Asian Journal of Kinesiology | This study was excluded because it did not provide the exposure on Tg/glucose or Tg/HDL index values. |
| 497 | Prevalence of metabolic syndrome and its associations with other metabolic disorders and cardiovascular changes in health examination population in Beijing | 2012 | Chin med sci j | This study was excluded because it did not provide the exposure on Tg/glucose or Tg/HDL index values. |
| 498 | The triglyceride-to-high density lipoprotein cholesterol ratio in overweight Korean children | 2015 | Hormone Research in Paediatrics | This study was excluded because it did not provide the exposure on Tg/glucose or Tg/HDL index values. |
| 499 | Metabolic syndrome in children and adolescents with phenylketonuria | 2015 | J pediatr (rio j) | This study was excluded because it did not provide the exposure on Tg/glucose or Tg/HDL index values. |
| 500 | Low vitamin D is associated with hypertension in paediatric obesity | 2015 | Journal of paediatrics and child health | This study was excluded because it did not provide the exposure on Tg/glucose or Tg/HDL index values. |
| 501 | Evaluation of micronutrient levels in children and adolescents with obesity and their correlation with the components of metabolic syndrome | 2021 | Turkish Journal of Pediatrics | This study was excluded because it did not provide the exposure on Tg/glucose or Tg/HDL index values. |
| 502 | Insulin resistance is not necessarily an essential element of metabolic syndrome | 2013 | Endocrine | This study was excluded because it did not provide the exposure on Tg/glucose or Tg/HDL index values. |
| 503 | The roles of triglyceride/high-density lipoprotein cholesterol ratio and uric acid as predisposing factors for metabolic syndrome in healthy children | 2019 | Annals of pediatric endocrinology & metabolism | This study was excluded because it did not provide the exposure on Tg/glucose or Tg/HDL index values. |
| 504 | Early appearance of the metabolic syndrome in socially reared bonnet macaques | 2005 | Journal of Clinical Endocrinology and Metabolism | This study was excluded because it did not provide the exposure on Tg/glucose or Tg/HDL index values. |
| 505 | Triglyceride: HDL-Cholesterol Ratio as a Surrogate Marker for Insulin Resistance and Inflammation in Pediatric Obesity | 2010 | Endocrine reviews | This study was excluded because it did not provide the exposure on Tg/glucose or Tg/HDL index values. |
| 506 | Low density lipoprotein particle diameter in young, nonobese, normolipidemic Japanese men | 1999 | Atherosclerosis | This study was excluded because it did not provide the exposure on Tg/glucose or Tg/HDL index values. |
| 507 | A Systematic Review of Single Nucleotide Polymorphisms Associated With Metabolic Syndrome in Children and Adolescents | 2018 | Journal of pediatrics review | This study was excluded because it did not provide the exposure on Tg/glucose or Tg/HDL index values. |
| 508 | Serum adiponectin is related to plasma high-density lipoprotein cholesterol but not to plasma insulin-concentration in healthy children: the FLVS II study | 2006 | Metabolism: Clinical and Experimental | This study was excluded because it did not provide the exposure on Tg/glucose or Tg/HDL index values. |
| 509 | Metabolic Risk Factors and Arterial Stiffness in Indian Children of Parents with Metabolic Syndrome | 2012 | Journal of the american college of nutrition | This study was excluded because it did not provide the exposure on Tg/glucose or Tg/HDL index values. |
| 510 | Lipid Biomarkers as Predictors of Diastolic Dysfunction in Diabetes with Poor Glycemic Control | 2020 | International journal of molecular sciences | This study was excluded because it did not provide the exposure on Tg/glucose or Tg/HDL index values. |
| 511 | Frequency of Dyslipidemia in non-obese Adolescence and its Association with family history of Diabetes | 2018 | Pakistan journal of medical & health sciences | This study was excluded because it did not provide the exposure on Tg/glucose or Tg/HDL index values. |
| 512 | Metabolic syndrome and insulin resistance in obese adolescent boys | 2016 | Monatsschrift fur Kinderheilkunde | This study was excluded because it did not provide the exposure on Tg/glucose or Tg/HDL index values. |
| 513 | Metabolic syndrome in adolescent boys: Diagnostics and treatment | 2009 | Journal of Diabetes | This study was excluded because it did not provide the exposure on Tg/glucose or Tg/HDL index values. |
| 514 | Associations between Fatness, Fitness, IGF and IMT among Obese Korean Male Adolescents | 2011 | Diabetes & metabolism journal | This study was excluded because it did not provide the exposure on Tg/glucose or Tg/HDL index values. |
| 515 | Association Between Diet Quality and Prevalence of Obesity, Dyslipidemia, and Insulin Resistance Among Filipino Immigrant Women in Korea: The Filipino Women's Diet and Health Study | 2021 | Frontiers in public health | This study was excluded because it did not provide the exposure on Tg/glucose or Tg/HDL index values. |
| 516 | 25-Hydroxyvitamin D status, metabolic syndrome and insulin resistance in preadolescent child in Korea | 2013 | International Journal of Pediatric Endocrinology | This study was excluded because it did not provide the exposure on Tg/glucose or Tg/HDL index values. |
| 517 | A Study of Serum Adiponectin and Insulin Resistance in Children and Adolescents | 2007 | Annals of Pediatirc Endocrinology & Metabolism | This study was excluded because it did not provide the exposure on Tg/glucose or Tg/HDL index values. |
| 518 | Effect of zinc supplementation on insulin resistance and metabolic risk factors in obese Korean women | 2012 | Nutrition research and practice | This study was excluded because it did not provide the exposure on Tg/glucose or Tg/HDL index values. |
| 519 | Association of abdominal fat distribution and cardiometabolic risk factors among obese Korean adolescents | 2008 | Diabetes and Metabolism | This study was excluded because it did not provide the exposure on Tg/glucose or Tg/HDL index values. |
| 520 | The cutoff values of indirect indices for measuring insulin resistance in Korean children and adolescents | 2016 | Hormone Research in Paediatrics | This study was excluded because it did not provide the exposure on Tg/glucose or Tg/HDL index values. |
| 521 | The cutoff values of indirect indices for measuring insulin resistance for metabolic syndrome in Korean children and adolescents | 2016 | Annals of Pediatric Endocrinology and Metabolism | This study was excluded because it did not provide the exposure on Tg/glucose or Tg/HDL index values. |
| 522 | Sex difference in levels of lipopolysaccharide binding protein as a marker of insulin resistance in Korean adolescents | 2018 | Endocrine reviews | This study was excluded because it did not provide the exposure on Tg/glucose or Tg/HDL index values. |
| 523 | Efficacy and Safety of Lobeglitazone Monotherapy in Patients with Type 2 Diabetes Mellitus over 24-Weeks: A Multicenter, Randomized, Double-Blind, Parallel-Group, Placebo Controlled Trial | 2014 | Plos one | This study was excluded because it did not provide the exposure on Tg/glucose or Tg/HDL index values. |
| 524 | Triglycerides/HDL cholesterol ratio and total cholesterol/HDL cholesterol ratio : ssurrogate markers for metabolic syndrome in adolescents | 2017 | International Journal of Pediatric Endocrinology | This study was excluded because it did not provide the exposure on Tg/glucose or Tg/HDL index values. |
| 525 | Higher Appendicular Skeletal Muscle Mass Protects Metabolically Healthy Obese Boys but Not Girls from Cardiometabolic Abnormality | 2019 | International journal of environmental research and public health | This study was excluded because it did not provide the exposure on Tg/glucose or Tg/HDL index values. |
| 526 | Association of physical activity and body mass index with metabolic syndrome among U.S. adolescents with disabilities | 2019 | Disabil health j | This study was excluded because it did not provide the exposure on Tg/glucose or Tg/HDL index values. |
| 527 | Should triglycerides and the triglycerides to high-density lipoprotein cholesterol ratio be used as surrogates for insulin resistance? | 2010 | Metabolism-clinical and experimental | This study was excluded because it did not provide the exposure on Tg/glucose or Tg/HDL index values. |
| 528 | PAI-1 gene 4G/5G polymorphism, cytokine levels and their relations with metabolic parameters in obese children | 2008 | Thrombosis and Haemostasis | This study was excluded because it did not provide the exposure on Tg/glucose or Tg/HDL index values. |
| 529 | Associations between serum uric acid concentrations and cardiometabolic risk and renal injury in obese and overweight children | 2019 | JCRPE Journal of Clinical Research in Pediatric Endocrinology | This study was excluded because it did not provide the exposure on Tg/glucose or Tg/HDL index values. |
| 530 | Can gamma-glutamil transferase serve as a biomarker of insulin resistance in overweight/obese adolescent girls? | 2017 | Archives of Disease in Childhood | This study was excluded because it did not provide the exposure on Tg/glucose or Tg/HDL index values. |
| 531 | Serum cystatin C levels are associated with triglycerides/high-density lipoprotein cholesterol ratio in adolescent girls ages between 16-19 years old | 2020 | European Review for Medical and Pharmacological Sciences | This study was excluded because it did not provide the exposure on Tg/glucose or Tg/HDL index values. |
| 532 | Acanthosis nigricans and insulin resistance in overweight children and adolescents | 2012 | Anais Brasileiros de Dermatologia | This study was excluded because it did not provide the exposure on Tg/glucose or Tg/HDL index values. |
| 533 | Lower circulating b12 is associated with higher obesity and insulin resistance during pregnancy in a non-diabetic white british population | 2015 | Plos ONE | This study was excluded because it did not provide the exposure on Tg/glucose or Tg/HDL index values. |
| 534 | Feasibility study of a school-based health center intervention to decrease metabolic syndrome risks in overweight/obese teens | 2011 | Journal of Diabetes | This study was excluded because it did not provide the exposure on Tg/glucose or Tg/HDL index values. |
| 535 | School-based health center intervention improves body mass index in overweight and obese adolescents | 2012 | Clinical and Translational Science | This study was excluded because it did not provide the exposure on Tg/glucose or Tg/HDL index values. |
| 536 | School-based health center intervention improves body mass index in overweight and obese adolescents | 2013 | Journal of Obesity | This study was excluded because it did not provide the exposure on Tg/glucose or Tg/HDL index values. |
| 537 | Acanthosis nigricans predicts the clustering of metabolic syndrome components in Hispanic elementary school-aged children | 2012 | Journal of Pediatric Endocrinology and Metabolism | This study was excluded because it did not provide the exposure on Tg/glucose or Tg/HDL index values. |
| 538 | [Clustering of metabolic risk factors and its related risk factors in young schoolchildren] | 2012 | J prev med public health | This study was excluded because it did not provide the exposure on Tg/glucose or Tg/HDL index values. |
| 539 | Comparison of the relative contributions of intra-abdominal and liver fat to components of the metabolic syndrome | 2011 | Obesity | This study was excluded because it did not provide the exposure on Tg/glucose or Tg/HDL index values. |
| 540 | Usefulness of the Triglycerides to High-Density Lipoprotein Cholesterol ratio (TG/HDL-C) in prediction of metabolic syndrome in Polish obese children and adolescents | 2018 | Acta biochimica Polonica | This study was excluded because it did not provide the exposure on Tg/glucose or Tg/HDL index values. |
| 541 | Obesity in African women in the North West Province, South Africa is associated with an increased risk of non-communicable diseases: The THUSA study | 2001 | British Journal of Nutrition | This study was excluded because it did not provide the exposure on Tg/glucose or Tg/HDL index values. |
| 542 | The effect of a one-year weight reduction program on serum uric acid in overweight/obese children and adolescents | 2011 | Clinical Chemistry and Laboratory Medicine | This study was excluded because it did not provide the exposure on Tg/glucose or Tg/HDL index values. |
| 543 | Serum TC/HDL-C, TG/HDL-C and LDL-C/HDL-C in predicting the risk of myocardial infarction in normolipidaemic patients in South Asia: A case-control study | 2008 | Asian pacific journal of tropical medicine | This study was excluded because it did not provide the exposure on Tg/glucose or Tg/HDL index values. |
| 544 | Clinical significance of neuregulin 4 (NRG4) in gestational diabetes mellitus | 2018 | Gynecological endocrinology | This study was excluded because it did not provide the exposure on Tg/glucose or Tg/HDL index values. |
| 545 | Neck circumference as a novel parameter to determine metabolic risk factors in obese children | 2012 | European Journal of Clinical Investigation | This study was excluded because it did not provide the exposure on Tg/glucose or Tg/HDL index values. |
| 546 | Association of Visceral Fat and Risk Factors for Metabolic Syndrome in Children and Adolescents | 2011 | Yonsei medical journal | This study was excluded because it did not provide the exposure on Tg/glucose or Tg/HDL index values. |
| 547 | YKL-40 is a marker of obesity and insulin resistance in obese prepubertal children | 2010 | Diabetes, Obesity and Metabolism | This study was excluded because it did not provide the exposure on Tg/glucose or Tg/HDL index values. |
| 548 | Effects of exercise in addition to a family-based lifestyle intervention program on hepatic fat in children with overweight | 2020 | Diabetes care | This study was excluded because it did not provide the exposure on Tg/glucose or Tg/HDL index values. |
| 549 | Liver enzymes and clustering cardiometabolic risk factors in European adolescents: the HELENA study | 2015 | Pediatric obesity | This study was excluded because it did not provide the exposure on Tg/glucose or Tg/HDL index values. |
| 550 | Heart rate recovery is associated with obesity traits and related cardiometabolic risk factors in children and adolescents | 2013 | Nutrition, Metabolism and Cardiovascular Diseases | This study was excluded because it did not provide the exposure on Tg/glucose or Tg/HDL index values. |
| 551 | Marked dyslipidemia in human immunodeficiency virus-infected children on protease inhibitor-containing antiretroviral therapy | 2002 | Pediatrics | This study was excluded because it did not provide the exposure on Tg/glucose or Tg/HDL index values. |
| 552 | Mild gestational diabetes mellitus and long-term child health | 2015 | Diabetes care | This study was excluded because it did not provide the exposure on Tg/glucose or Tg/HDL index values. |
| 553 | Evaluation of insulin resistance and metabolic syndrome in patients with polycystic ovary syndrome | 2009 | Gynecological endocrinology | This study was excluded because it did not provide the exposure on Tg/glucose or Tg/HDL index values. |
| 554 | Change in plasma atherogenic index after 1-year intervention based on Mediterranean diet in obese children | 2017 | Journal of Pediatric Gastroenterology and Nutrition | This study was excluded because it did not provide the exposure on Tg/glucose or Tg/HDL index values. |
| 555 | Cardiovascular risks in adolescents with different degrees of obesity | 2011 | Arquivos Brasileiros de Cardiologia | This study was excluded because it did not provide the exposure on Tg/glucose or Tg/HDL index values. |
| 556 | The association of life course socio-economic position with diagnosis, treatment, control and survival of women with diabetes: findings from the British Women's Heart and Health Study | 2007 | Diabetic medicine | This study was excluded because it did not provide the exposure on Tg/glucose or Tg/HDL index values. |
| 557 | The association of fasting insulin, glucose, and lipids with bone mass in adolescents: Findings from a cross-sectional study | 2012 | Journal of Clinical Endocrinology and Metabolism | This study was excluded because it did not provide the exposure on Tg/glucose or Tg/HDL index values. |
| 558 | Thyrotropin Levels Are Associated with Cardiometabolic Risk Factors in Euthyroid Adolescents | 2016 | Thyroid | This study was excluded because it did not provide the exposure on Tg/glucose or Tg/HDL index values. |
| 559 | Traditional anthropometric parameters still predict metabolic disorders in women with severe obesity | 2010 | Obesity | This study was excluded because it did not provide the exposure on Tg/glucose or Tg/HDL index values. |
| 560 | Association of serum retinol binding protein 4 and insulin resistance in apparently healthy adolescents | 2007 | Metabolism: Clinical and Experimental | This study was excluded because it did not provide the exposure on Tg/glucose or Tg/HDL index values. |
| 561 | 25-Hydroxyvitamin D and metabolic risk in children | 2012 | Hormone Research in Paediatrics | This study was excluded because it did not provide the exposure on Tg/glucose or Tg/HDL index values. |
| 562 | Lipid-derived surrogate markers to identify insulin resistance and metabolic syndrome in children and adolescents | 2018 | Endocrine reviews | This study was excluded because it did not provide the exposure on Tg/glucose or Tg/HDL index values. |
| 563 | What is the appropriate strategy for diagnosing NAFLD using ultrasonography in obese children? | 2017 | World journal of pediatrics | This study was excluded because it did not provide the exposure on Tg/glucose or Tg/HDL index values. |
| 564 | Sex-specific relationships between insulin resistance and bone mineral content in Korean adolescents | 2013 | Journal of Bone and Mineral Metabolism | This study was excluded because it did not provide the exposure on Tg/glucose or Tg/HDL index values. |
| 565 | Metabolic Syndrome Parameters in adolescents may be determinants for the future periodontal diseases | 2015 | Journal of clinical periodontology | This study was excluded because it did not provide the exposure on Tg/glucose or Tg/HDL index values. |
| 566 | Relationship between Childhood and Adolescent Obesity and Remnant Lipoprotein | 2006 | Endocrinology and Metabolism | This study was excluded because it did not provide the exposure on Tg/glucose or Tg/HDL index values. |
| 567 | Waist circumference, blood pressure, and lipid components of the metabolic syndrome | 2006 | Journal of pediatrics | This study was excluded because it did not provide the exposure on Tg/glucose or Tg/HDL index values. |
| 568 | Elevated serum gamma-glutamyltransferase is a strong marker of insulin resistance in obese children | 2013 | International Journal of Endocrinology | This study was excluded because it did not provide the exposure on Tg/glucose or Tg/HDL index values. |
| 569 | Elevated serum gamma-glutamyltransferase is a stronger predictor of insulin resistance among hepatic enzymes in obese children | 2010 | Obesity reviews | This study was excluded because it did not provide the exposure on Tg/glucose or Tg/HDL index values. |
| 570 | Changes in renal glucose transporters in an animal model of metabolic syndrome | 2013 | Hormone and Metabolic Research | This study was excluded because it did not provide the exposure on Tg/glucose or Tg/HDL index values. |
| 571 | Frequency of metabolic syndrome in children and adolescents from public schools of Curitiba - Brazil | 2011 | Journal of Diabetes | This study was excluded because it did not provide the exposure on Tg/glucose or Tg/HDL index values. |
| 572 | Effect of Aqueous Terminalia sericea Leaf Extract on Visceral Obesity in Fructose-fed Wistar Rats | 2019 | Journal of Herbs, Spices and Medicinal Plants | This study was excluded because it did not provide the exposure on Tg/glucose or Tg/HDL index values. |
| 573 | Lung Function Impairment and Metabolic Syndrome The Critical Role of Abdominal Obesity | 2009 | American journal of respiratory and critical care medicine | This study was excluded because it did not provide the exposure on Tg/glucose or Tg/HDL index values. |
| 574 | The Fatty Liver Index (FLI) Relates to Diabetes-Specific Parameters and an Adverse Lipid Profile in a Cohort of Nondiabetic, Dyslipidemic Patients | 2017 | Journal of the american college of nutrition | This study was excluded because it did not provide the exposure on Tg/glucose or Tg/HDL index values. |
| 575 | Adipokines and incident type 2 diabetes in an aboriginal canadian population: The sandy lake health and diabetes project | 2008 | Diabetes care | This study was excluded because it did not provide the exposure on Tg/glucose or Tg/HDL index values. |
| 576 | Prediction of metabolically unhealthy phenotype by the visceral adiposity index, lipid accumulation product and triglycerideglucose index in Chinese women with polycystic ovary syndrome | 2017 | Human reproduction | This study was excluded because it did not provide the exposure on Tg/glucose or Tg/HDL index values. |
| 577 | Green tea leaf extract improves lipid and glucose homeostasis in a fructose-fed insulin-resistant hamster model | 2006 | Journal of Ethnopharmacology | This study was excluded because it did not provide the exposure on Tg/glucose or Tg/HDL index values. |
| 578 | Triglyceride to high-density lipoprotein cholesterol ratio and carotid intima-medial thickness in Chinese adolescents with newly diagnosed type 2 diabetes mellitus | 2016 | Pediatric diabetes | This study was excluded because it did not provide the exposure on Tg/glucose or Tg/HDL index values. |
| 579 | Association between triglyceride glucose-body mass index and non-alcoholic fatty liver disease in the non-obese Chinese population with normal blood lipid levels: a secondary analysis based on a prospective cohort study | 2020 | Lipids in Health and Disease | This study was excluded because it did not provide the exposure on Tg/glucose or Tg/HDL index values. |
| 580 | TriGlycerides and high-density lipoprotein cholesterol ratio compared with homeostasis model assessment insulin resistance indexes in screening for metabolic syndrome in the chinese obese children: A cross section study | 2015 | Bmc pediatrics | This study was excluded because it did not provide the exposure on Tg/glucose or Tg/HDL index values. |
| 581 | Ratio of triglycerides to high-density lipoprotein cholesterol compared with homeostasis model assessment insulin resistance indexes in screening the metabolic syndrome in Chinese obese children | 2014 | Hormone Research in Paediatrics | This study was excluded because it did not provide the exposure on Tg/glucose or Tg/HDL index values. |
| 582 | Abdominal adipose tissue distribution and metabolic syndrome burden in adolescents-Penn state child cohort (PSCC) study | 2014 | Circulation | This study was excluded because it did not provide the exposure on Tg/glucose or Tg/HDL index values. |
| 583 | Efficacy of anthropometric measures for identifying cardiovascular disease risk in adolescents: review and meta-analysis | 2018 | Minerva pediatrica | This study was excluded because it did not provide the exposure on Tg/glucose or Tg/HDL index values. |
| 584 | Association of thyroid stimulating hormone and free thyroxine with cardiometabolic risk factors in euthyroid Korean children and adolescents aged 10-18 years: The Korean national health and nutrition examination survey 2015 | 2018 | Hormone Research in Paediatrics | This study was excluded because it did not provide the exposure on Tg/glucose or Tg/HDL index values. |
| 585 | Weight change and cardiometabolic outcomes in postpartumwomen with history of gestational diabetes | 2019 | Nutrients | This study was excluded because it did not provide the exposure on Tg/glucose or Tg/HDL index values. |
| 586 | Efficacy of UB0316, a multi-strain probiotic formulation in patients with type 2 diabetes mellitus: A double blind, randomized, placebo controlled study | 2017 | International Journal of Cardiovascular Sciences | This study was excluded because it did not provide the exposure on Tg/glucose or Tg/HDL index values. |
| 587 | [Physical activities and dietary intervention on metabolic syndrome in children] | 2017 | Zhonghua liu xing bing xue za zhi | This study was excluded because it did not provide the exposure on Tg/glucose or Tg/HDL index values. |
| 588 | Peripheral metabolic state and immune system in first-episode psychosis - A gene expression study with a prospective one-year follow-up | 2021 | Journal of psychiatric research | This study was excluded because it did not provide the exposure on Tg/glucose or Tg/HDL index values. |
| 589 | Peripheral metabolic state and immune system in first-episode psychosis - A gene expression study with a prospective one-year follow-up | 2021 | Journal of Psychiatric Research | This study was excluded because it did not provide the exposure on Tg/glucose or Tg/HDL index values. |
| 590 | Effects of R219K polymorphism of ATP-binding cassette transporter 1 gene on serum lipids ratios induced by a high-carbohydrate and low-fat diet in healthy youth | 2014 | Biological research | This study was excluded because it did not provide the exposure on Tg/glucose or Tg/HDL index values. |
| 591 | Effects of apolipoprotein e genotypes on metabolic profile and oxidative stress in south-west Chinese women with polycystic ovary syndrome | 2013 | European Journal of Obstetrics and Gynecology and Reproductive Biology | This study was excluded because it did not provide the exposure on Tg/glucose or Tg/HDL index values. |
| 592 | The predictive ability of two triglyceride-associated indices for gestational diabetes mellitus and large for gestational age infant among Chinese pregnancies: A preliminary cohort study | 2020 | Diabetes, Metabolic Syndrome and Obesity: Targets and Therapy | This study was excluded because it did not provide the exposure on Tg/glucose or Tg/HDL index values. |
| 593 | Do all components of the metabolic syndrome cluster together in US Hispanics/Latinos? Results from the Hispanic Community Health study/Study of Latinos | 2015 | Annals of epidemiology | This study was excluded because it did not provide the exposure on Tg/glucose or Tg/HDL index values. |
| 594 | Triglyceride/glucose index is a reliable alternative marker for insulin resistance in South American overweight and obese children and adolescents | 2019 | Journal of Pediatric Endocrinology and Metabolism | Included |
| 595 | Does Nox2 Overactivate in Children with Nonalcoholic Fatty Liver Disease | 2019 | Antioxidants and Redox Signaling | This study was excluded because it did not provide the exposure on Tg/glucose or Tg/HDL index values. |
| 596 | Hyperinsulinemia and Waist Circumference in Childhood Metabolic Syndrome | 2011 | Jcpsp-journal of the college of physicians and surgeons pakistan | This study was excluded because it did not provide the exposure on Tg/glucose or Tg/HDL index values. |
| 597 | Cortisol/cortisone ratio in overweight and obese adolescents | 2017 | Endocrine reviews | This study was excluded because it did not provide the exposure on Tg/glucose or Tg/HDL index values. |
| 598 | Hemodynamic and metabolic profile in offspring of malignant hypertensive parents | 2001 | Hypertension | This study was excluded because it did not provide the exposure on Tg/glucose or Tg/HDL index values. |
| 599 | TyG in insulin resistance prediction | 2020 | Jornal de pediatria | This study was excluded because it did not provide the exposure on Tg/glucose or Tg/HDL index values. |
| 600 | Hiperandrogenemia is highly frequent in adolescent girls who are obese | 2016 | Faseb journal | This study was excluded because it did not provide the exposure on Tg/glucose or Tg/HDL index values. |
| 601 | Physical Activity, Fitness, and Metabolic Syndrome in Young Adults | 2013 | International journal of sport nutrition and exercise metabolism | This study was excluded because it did not provide the exposure on Tg/glucose or Tg/HDL index values. |
| 602 | Fractional excretion of sodium as a new component of the metabolic syndrome in pediatric population | 2012 | Hormone Research in Paediatrics | This study was excluded because it did not provide the exposure on Tg/glucose or Tg/HDL index values. |
| 603 | Nonalcoholic fatty liver disease: A novel risk factor for the development of type 2 diabetes in childhood? | 2013 | Pediatric diabetes | This study was excluded because it did not provide the exposure on Tg/glucose or Tg/HDL index values. |
| 604 | Non-alcoholic fatty liver disease: A novel risk factor for the development of type 2 diabetes in childhood? | 2013 | Hormone Research in Paediatrics | This study was excluded because it did not provide the exposure on Tg/glucose or Tg/HDL index values. |
| 605 | Hepatic steatosis as diabetes type 2 predictor | 2014 | Nutricion hospitalaria | This study was excluded because it did not provide the exposure on Tg/glucose or Tg/HDL index values. |
| 606 | Blood glucose and insulin and correlation of SLC25A13 mutations with biochemical changes in NICCD patients | 2017 | Experimental Biology and Medicine | This study was excluded because it did not provide the exposure on Tg/glucose or Tg/HDL index values. |
| 607 | Protective effect of vanillin in streptozotocin-induced diabetes in neonatal rats via attenuation of oxidative stress and inflammation | 2019 | Tropical Journal of Pharmaceutical Research | This study was excluded because it did not provide the exposure on Tg/glucose or Tg/HDL index values. |
| 608 | Genetic deletion of syndecan-4 alters body composition, metabolic phenotypes, and the function of metabolic tissues in female mice fed a high-fat diet (Running title: Sdc4 deficiency affects metabolic phenotypes) | 2019 | Nutrients | This study was excluded because it did not provide the exposure on Tg/glucose or Tg/HDL index values. |
| 609 | Association of insulin resistance with dyslipidemia in adults with type 1 diabetes | 2019 | Zhonghua yi xue za zhi | This study was excluded because it did not provide the exposure on Tg/glucose or Tg/HDL index values. |
| 610 | Dyslipidaemia was correlated to the posterior circulation infarction in non-diabetic populations | 2018 | Lipids in health and disease | This study was excluded because it did not provide the exposure on Tg/glucose or Tg/HDL index values. |
| 611 | The metabolic syndrome and related characteristics in major depression: inpatients and outpatients compared: metabolic differences across treatment settings | 2018 | Gen hosp psychiatry | This study was excluded because it did not provide the exposure on Tg/glucose or Tg/HDL index values. |
| 612 | Association of insulin sensitivity to lipids across the lifespan in people with type 1 diabetes | 2010 | Diabetes | This study was excluded because it did not provide the exposure on Tg/glucose or Tg/HDL index values. |
| 613 | Association of insulin sensitivity to lipids across the lifespan in people with Type 1 diabetes | 2011 | Diabetic medicine | This study was excluded because it did not provide the exposure on Tg/glucose or Tg/HDL index values. |
| 614 | Vitamin D Status, Cardiometabolic, Liver, and Mental Health Status in Obese Youth Attending a Pediatric Weight Management Center | 2017 | Journal of Pediatric Gastroenterology and Nutrition | This study was excluded because it did not provide the exposure on Tg/glucose or Tg/HDL index values. |
| 615 | Efficacy of UB0316, a multi-strain probiotic formulation in patients with type 2 diabetes mellitus: A double blind, randomized, placebo controlled study | 2019 | Plos one | This study was excluded because it did not provide the exposure on Tg/glucose or Tg/HDL index values. |
| 616 | Study on the prevalence and characteristics of metabolic syndrome in women of reproductive age group with polycystic ovarian syndrome | 2017 | Journal of SAFOG | This study was excluded because it did not provide the exposure on Tg/glucose or Tg/HDL index values. |
| 617 | Changes in cardiorespiratory fitness through adolescence predict metabolic syndrome in young adults | 2020 | Nutrition, Metabolism and Cardiovascular Diseases | This study was excluded because it did not provide the exposure on Tg/glucose or Tg/HDL index values. |
| 618 | Lipid and saturated fatty acids intake and cardiovascular risk factors of obese children and adolescents | 2020 | European Journal of Clinical Nutrition | This study was excluded because it did not provide the exposure on Tg/glucose or Tg/HDL index values. |
| 619 | Anthropometric measures of visceral and subcutaneous fat are important in the determination of metabolic dysregulation in boys and girls at risk for nonalcoholic fatty liver disease | 2013 | Nutrition in Clinical Practice | This study was excluded because it did not provide the exposure on Tg/glucose or Tg/HDL index values. |
| 620 | Cardiometabolic risk and body composition in youth with down syndrome | 2019 | Pediatrics | This study was excluded because it did not provide the exposure on Tg/glucose or Tg/HDL index values. |
| 621 | Increased Pancreatic Fat Fraction Is Present in Obese Adolescents With Metabolic Syndrome | 2012 | Journal of pediatric gastroenterology and nutrition | This study was excluded because it did not provide the exposure on Tg/glucose or Tg/HDL index values. |
| 622 | Effect of obesity on the plasma lipoprotein subclass profile in normoglycemic and normolipidemic men and women | 2008 | International journal of obesity | This study was excluded because it did not provide the exposure on Tg/glucose or Tg/HDL index values. |
| 623 | Associations of obesity with triglycerides and C-reactive protein are attenuated in adults with high red blood cell eicosapentaenoic and docosahexaenoic acids | 2011 | European journal of clinical nutrition | This study was excluded because it did not provide the exposure on Tg/glucose or Tg/HDL index values. |
| 624 | Sphingomyelin is associated with kidney disease in type 1 diabetes (The FinnDiane Study) | 2012 | Metabolomics | This study was excluded because it did not provide the exposure on Tg/glucose or Tg/HDL index values. |
| 625 | Non-alcoholic fatty liver disease in obese and overweight iranian children: A cross sectional study | 2019 | Archives of Disease in Childhood | This study was excluded because it did not provide the exposure on Tg/glucose or Tg/HDL index values. |
| 626 | Triglycerides-to-HDL cholesterol ratio as screening tool for impaired glucose tolerance in obese children and adolescents | 2016 | Acta diabetologica | This study was excluded because it did not provide the exposure on Tg/glucose or Tg/HDL index values. |
| 627 | Arterial Stiffness, Thickness and Association to Suitable Novel Markers of Risk at the Origin of Cardiovascular Disease in Obese Children | 2017 | International journal of medical sciences | This study was excluded because it did not provide the exposure on Tg/glucose or Tg/HDL index values. |
| 628 | Gap analysis of pediatric reference intervals for risk biomarkers of cardiovascular disease and the metabolic syndrome | 2006 | Clinical biochemistry | This study was excluded because it did not provide the exposure on Tg/glucose or Tg/HDL index values. |
| 629 | Higher serum DHEAS concentration is associated with lower plasma LDL cholesterol concentration in children | 2015 | Hormone Research in Paediatrics | This study was excluded because it did not provide the exposure on Tg/glucose or Tg/HDL index values. |
| 630 | Limited Weight Loss or Simply No Weight Gain following Lifestyle-Only Intervention Tends to Redistribute Body Fat, to Decrease Lipid Concentrations, and to Improve Parameters of Insulin Sensitivity in Obese Children | 2011 | International journal of pediatric endocrinology | This study was excluded because it did not provide the exposure on Tg/glucose or Tg/HDL index values. |
| 631 | Limited weight loss or simply no weight gain following lifestyle-only intervention tends to redistribute body fat, to decrease lipid levels and to improve parameters of insulin sensitivity in obese children | 2010 | Hormone Research in Paediatrics | This study was excluded because it did not provide the exposure on Tg/glucose or Tg/HDL index values. |
| 632 | One-hour post-load plasma glucose levels associated with decreased insulin sensitivity and secretion and early makers of cardiometabolic risk | 2017 | Journal of Endocrinological Investigation | This study was excluded because it did not provide the exposure on Tg/glucose or Tg/HDL index values. |
| 633 | Insulin resistance and cardiometabolic risk factors in obese children and adolescents | 2015 | Hormone Research in Paediatrics | This study was excluded because it did not provide the exposure on Tg/glucose or Tg/HDL index values. |
| 634 | Lean Muscle Mass in Classic or Ovulatory PCOS: Association with Central Obesity and Insulin Resistance | 2012 | Experimental and clinical endocrinology & diabetes | This study was excluded because it did not provide the exposure on Tg/glucose or Tg/HDL index values. |
| 635 | No association of the G972S polymorphism of the insulin receptor substrate-1 gene with polycystic ovary syndrome in lean PCOS women with biochemical hyperandrogenemia | 2010 | Archives of gynecology and obstetrics | This study was excluded because it did not provide the exposure on Tg/glucose or Tg/HDL index values. |
| 636 | Breakfast and fast food consumption are associated with selected biomarkers in adolescents | 2016 | Preventive medicine reports | This study was excluded because it did not provide the exposure on Tg/glucose or Tg/HDL index values. |
| 637 | Association between CAPN10 UCSNP-43 gene polymorphism and polycystic ovary syndrome in Chilean women | 2008 | Clinica chimica acta | This study was excluded because it did not provide the exposure on Tg/glucose or Tg/HDL index values. |
| 638 | Obese children displayed deterioreted reverse cholesterol transport in association with insulin resistence, vascular inflammation and altered lipid metabolism | 2020 | Atherosclerosis | This study was excluded because it did not provide the exposure on Tg/glucose or Tg/HDL index values. |
| 639 | Effect of the oral administration of ascorbic acid on lipid profile and insulin sensitivity in obese people | 2001 | Revista de investigacion clinica-clinical and translational investigation | This study was excluded because it did not provide the exposure on Tg/glucose or Tg/HDL index values. |
| 640 | [Effect of oral administration of ascorbic acid on insulin sensitivity and lipid profile in obese individuals] | 2001 | Rev invest clin | This study was excluded because it did not provide the exposure on Tg/glucose or Tg/HDL index values. |
| 641 | Sedentary behavior, adiposity and cardiovascular risk factors in adolescents. The AFINOS study | 2010 | Revista Espanola de Cardiologia | This study was excluded because it did not provide the exposure on Tg/glucose or Tg/HDL index values. |
| 642 | Toledo area study: Relationship between physical activity and cardiovascular risk factors in adolescents | 2013 | Annals of Nutrition and Metabolism | This study was excluded because it did not provide the exposure on Tg/glucose or Tg/HDL index values. |
| 643 | Validity of a single-factor model underlying the metabolic syndrome in children: a confirmatory factor analysis | 2013 | Diabetes care | This study was excluded because it did not provide the exposure on Tg/glucose or Tg/HDL index values. |
| 644 | Stability of the factorial structure of metabolic syndrome from childhood to adolescence: a 6-year follow-up study | 2013 | Cardiovasc diabetol | This study was excluded because it did not provide the exposure on Tg/glucose or Tg/HDL index values. |
| 645 | Gender differences on effectiveness of a school-based physical activity intervention for reducing cardiometabolic risk: a cluster randomized trial | 2013 | Int j behav nutr phys act | This study was excluded because it did not provide the exposure on Tg/glucose or Tg/HDL index values. |
| 646 | Is it possible a new definition of metabolic syndrome in childhood? | 2015 | European Review for Medical and Pharmacological Sciences | This study was excluded because it did not provide the exposure on Tg/glucose or Tg/HDL index values. |
| 647 | Metabolic syndrome among children and adolescents from Southern Italy: contribution from the Calabrian Sierras Community Study (CSCS) | 2015 | Int j cardiol | This study was excluded because it did not provide the exposure on Tg/glucose or Tg/HDL index values. |
| 648 | Late hyperinsulinemic response in the OGTT is associated to lower insulin sensitivity and a worse metabolic profile in obese chidren | 2017 | Hormone Research in Paediatrics | This study was excluded because it did not provide the exposure on Tg/glucose or Tg/HDL index values. |
| 649 | The triglyceride-to-HDL cholesterol ratio is associated with insulin resistance in obese boys but not in obese girls | 2015 | Hormone Research in Paediatrics | This study was excluded because it did not provide the exposure on Tg/glucose or Tg/HDL index values. |
| 650 | Association of circulating growth arrest-specific 6 protein with adiposity and metabolic syndrome in obese children and adolescents | 2018 | Obesity facts | This study was excluded because it did not provide the exposure on Tg/glucose or Tg/HDL index values. |
| 651 | Gas6 protein in adiposity and metabolic syndrome in childhood obesity | 2018 | Annals of Nutrition and Metabolism | This study was excluded because it did not provide the exposure on Tg/glucose or Tg/HDL index values. |
| 652 | Cardiovascular Disease Risk Factors in Youth With Type 1 and Type 2 Diabetes: Implications of a Factor Analysis of Clustering | 2009 | Metabolic syndrome and related disorders | This study was excluded because it did not provide the exposure on Tg/glucose or Tg/HDL index values. |
| 653 | Association of triglyceride/HDL-cholesterol ratio with insulin resistance indices in obese children | 2005 | Journal of investigative medicine | This study was excluded because it did not provide the exposure on Tg/glucose or Tg/HDL index values. |
| 654 | Health Biomarkers in Adults Are More Closely Linked to Diet Quality Attributes Than to Plant-Based Diet Categorization | 2019 | Nutrients | This study was excluded because it did not provide the exposure on Tg/glucose or Tg/HDL index values. |
| 655 | Effect of Dietary Insulinemia on All-Cause and Cause-Specific Mortality: Results From a Cohort Study | 2020 | Journal of the american college of nutrition | This study was excluded because it did not provide the exposure on Tg/glucose or Tg/HDL index values. |
| 656 | Transcriptional correlates of linear growth and lipid homeostasis in individuals with mitochondrial respiratory chain disease | 2014 | Molecular Genetics and Metabolism | This study was excluded because it did not provide the exposure on Tg/glucose or Tg/HDL index values. |
| 657 | Inflammation is an independent predictor of carotid intima media thickness progression in early young adulthood | 2013 | Circulation | This study was excluded because it did not provide the exposure on Tg/glucose or Tg/HDL index values. |
| 658 | Pathogenesis and management of dyslipidemia in obese children | 2018 | American Journal of Cardiology | This study was excluded because it did not provide the exposure on Tg/glucose or Tg/HDL index values. |
| 659 | Mexican American Children Have Differential Elevation of Metabolic Biomarkers Proportional to Obesity Status | 2013 | Journal of pediatric gastroenterology and nutrition | This study was excluded because it did not provide the exposure on Tg/glucose or Tg/HDL index values. |
| 660 | Is there a simple way to identify insulin-resistant individuals at increased risk of cardiovascular disease? | 2005 | American Journal of Cardiology | This study was excluded because it did not provide the exposure on Tg/glucose or Tg/HDL index values. |
| 661 | Persistence of improvement in insulin sensitivity following a dietary weight loss programme | 2008 | Diabetes obesity & metabolism | This study was excluded because it did not provide the exposure on Tg/glucose or Tg/HDL index values. |
| 662 | Associations of physical activity and fitness with hepatic steatosis, liver enzymes, and insulin resistance in children with overweight/obesity | 2020 | Pediatric diabetes | This study was excluded because it did not provide the exposure on Tg/glucose or Tg/HDL index values. |
| 663 | RELATION OF SERUM TRIGLYCERIDES IN CAUSING INSULIN RESISTANCE AMONG PATIENTS WITH CIRRHOSIS IN A TERTIARY CARE CENTER | 2016 | Journal of evolution of medical and dental sciences-jemds | This study was excluded because it did not provide the exposure on Tg/glucose or Tg/HDL index values. |
| 664 | Non-alcoholic fatty liver disease in Asian Indian adolescents and young adults: Prevalence and its associated risk factors | 2021 | Journal of diabetology | This study was excluded because it did not provide the exposure on Tg/glucose or Tg/HDL index values. |
| 665 | Clustering of the metabolic syndrome components in adolescence: Role of visceral fat | 2013 | Plos ONE | This study was excluded because it did not provide the exposure on Tg/glucose or Tg/HDL index values. |
| 666 | Novel modeling of reference values of cardiovascular risk factors in children aged 7 to 20 years | 2012 | Pediatrics | This study was excluded because it did not provide the exposure on Tg/glucose or Tg/HDL index values. |
| 667 | Waist circumference is more important than insulin resistance in the evaluation of the cardiometabolic risk index in adolescents | 2012 | Circulation | This study was excluded because it did not provide the exposure on Tg/glucose or Tg/HDL index values. |
| 668 | A 12-Month Randomized, Open-Label Study of the Metabolic Effects of Olanzapine and Risperidone in Psychotic Patients: Influence of Valproic Acid Augmentation | 2011 | Journal of clinical psychiatry | This study was excluded because it did not provide the exposure on Tg/glucose or Tg/HDL index values. |
| 669 | Triglyceride-glucose index is related to neonatal overgrowth in mothers with gestational diabetes | 2015 | Diabetologia | This study was excluded because it did not provide the exposure on Tg/glucose or Tg/HDL index values. |
| 670 | Association of glucose homeostasis measures and metabolic syndrome with knee cartilage defects and cartilage volume in young adults | 2019 | Annals of the Rheumatic Diseases | This study was excluded because it did not provide the exposure on Tg/glucose or Tg/HDL index values. |
| 671 | Fat and carbohydrate content in the diet induces drastic changes in gene expression in young Gottingen minipigs | 2017 | Mammalian genome | This study was excluded because it did not provide the exposure on Tg/glucose or Tg/HDL index values. |
| 672 | Steroid withdrawal in pediatric kidney transplant allows better growth, lipids and body composition: A randomized controlled trial | 2013 | Hormone Research in Paediatrics | This study was excluded because it did not provide the exposure on Tg/glucose or Tg/HDL index values. |
| 673 | Benefits of 1yr steroid withdrawal (SW) are translated into better longitudinal growth, lipid profile and trunk fat in pediatric kidney transplant recipients | 2012 | Hormone Research in Paediatrics | This study was excluded because it did not provide the exposure on Tg/glucose or Tg/HDL index values. |
| 674 | Effect of 1yr steroid withdrawal on growth, growth factors, insulin sensitivity (IS) and body composition in pediatric kidney transplantation | 2012 | Hormone Research in Paediatrics | This study was excluded because it did not provide the exposure on Tg/glucose or Tg/HDL index values. |
| 675 | Anthropometric determinants of a clustering of lipid-related metabolic risk factors in overweight and non-overweight adolescents influence of cardiorespiratory fitness - The AVENA study | 2006 | Annals of nutrition and metabolism | This study was excluded because it did not provide the exposure on Tg/glucose or Tg/HDL index values. |
| 676 | The relationship between homeostasis model assessment and cardiovascular risk factors in Iranian subjects with normal fasting glucose and normal glucose tolerance | 2006 | Clinica chimica acta | This study was excluded because it did not provide the exposure on Tg/glucose or Tg/HDL index values. |
| 677 | Prevalence of Metabolic Syndrome in US-Born Latin and Caribbean Youth | 2009 | Journal of immigrant and minority health | This study was excluded because it did not provide the exposure on Tg/glucose or Tg/HDL index values. |
| 678 | Lipid levels in former gestational diabetic mothers | 1996 | Diabetes care | This study was excluded because it did not provide the exposure on Tg/glucose or Tg/HDL index values. |
| 679 | Visceral adiposity index (VAI) in youth: A biomarker of metabolic syndrome and type 2 diabetes (T2D) | 2017 | Diabetes | This study was excluded because it did not provide the exposure on Tg/glucose or Tg/HDL index values. |
| 680 | Plasma 25-hydroxyvitamin D concentration and prevalence of metabolic syndrome in the diabetes prevention program randomized controlled trial | 2013 | Salus | This study was excluded because it did not provide the exposure on Tg/glucose or Tg/HDL index values. |
| 681 | Adiponectin: Leptin ratio reflects insulin resistance in obese adolescents | 2011 | Obesity | This study was excluded because it did not provide the exposure on Tg/glucose or Tg/HDL index values. |
| 682 | Cutoff point estimation for serum vitamin D concentrations to predict cardiometabolic risk in Brazilian children | 2020 | European journal of clinical nutrition | This study was excluded because it did not provide the exposure on Tg/glucose or Tg/HDL index values. |
| 683 | Could phthalates exposure contribute to the development of metabolic syndrome and liver disease in humans? | 2020 | Environmental science and pollution research | This study was excluded because it did not provide the exposure on Tg/glucose or Tg/HDL index values. |
| 684 | Overweight, the major determinant of metabolic syndrome among industrial workers in Kerala, India: Results of a cross-sectional study | 2019 | Diabetes & metabolic syndrome-clinical research & reviews | This study was excluded because it did not provide the exposure on Tg/glucose or Tg/HDL index values. |
| 685 | A Community-Based Study of Metabolic Syndrome and Its Components Among Women of Rural Community in Ballabgarh, Haryana | 2011 | Metabolic syndrome and related disorders | This study was excluded because it did not provide the exposure on Tg/glucose or Tg/HDL index values. |
| 686 | Plasma 25-hydroxyvitamin D concentration and prevalence of metabolic syndrome in the diabetes prevention program randomized controlled trial | 2012 | Endocrine reviews | This study was excluded because it did not provide the exposure on Tg/glucose or Tg/HDL index values. |
| 687 | The influence of obesity and metabolic risk variables on brachial-ankle pulse wave velocity in healthy adolescents | 2009 | Journal of Human Hypertension | This study was excluded because it did not provide the exposure on Tg/glucose or Tg/HDL index values. |
| 688 | The relation between visceral adipose tissue accumulation and biochemical tests in university students | 2005 | Acta medica okayama | This study was excluded because it did not provide the exposure on Tg/glucose or Tg/HDL index values. |
| 689 | Age- and sex-specific prevalence and ten-year risk for cardiovascular disease of all 16 risk factor combinations of the metabolic syndrome - A cross-sectional study | 2010 | Cardiovascular diabetology | This study was excluded because it did not provide the exposure on Tg/glucose or Tg/HDL index values. |
| 690 | Serum Ferritin Correlates With Liver Fat in Male Adolescents With Obesity | 2020 | Frontiers in endocrinology | This study was excluded because it did not provide the exposure on Tg/glucose or Tg/HDL index values. |
| 691 | Is there an increased risk of metabolic syndrome among childhood acute lymphoblastic leukemia survivors? A developing country experience | 2016 | Pediatric Hematology and Oncology | This study was excluded because it did not provide the exposure on Tg/glucose or Tg/HDL index values. |
| 692 | Triglyceride glucose index as a surrogate measure of insulin sensitivity in obese adolescents with normoglycemia, prediabetes, and type 2 diabetes mellitus: Comparison with the hyperinsulinemic-euglycemic clamp | 2015 | Pediatric diabetes | This study was excluded because it did not provide the exposure on Tg/glucose or Tg/HDL index values. |
| 693 | Triglyceride glucose index as a surrogate measure of insulin sensitivity in obese adolescents with normoglycemia, prediabetes, and type 2 diabetes mellitus: comparison with the hyperinsulinemic–euglycemic clamp | 2016 | Pediatric diabetes | This study was excluded because it did not provide the exposure on Tg/glucose or Tg/HDL index values. |
| 694 | Lean adolescents with increased risk for metabolic syndrome | 2003 | Archivos Latinoamericanos de Nutricion | This study was excluded because it did not provide the exposure on Tg/glucose or Tg/HDL index values. |
| 695 | Associations of insulin-like growth factor binding protein-3 gene polymorphisms with IGF-I activity and lipid parameters in adolescents | 2009 | International Journal of Obesity | This study was excluded because it did not provide the exposure on Tg/glucose or Tg/HDL index values. |
| 696 | Metabolic risks of oral contraception | 1988 | Revue internationale de pediatrie | This study was excluded because it did not provide the exposure on Tg/glucose or Tg/HDL index values. |
| 697 | Differences in cardiovascular risks in the aboriginal and non-aboriginal people living in Bella Coola, British Columbia | 2005 | Medical science monitor | This study was excluded because it did not provide the exposure on Tg/glucose or Tg/HDL index values. |
| 698 | The Cut-off Values of Triglycerides and Glucose Index for Metabolic Syndrome in American and Korean Adolescents | 2017 | Journal of Korean medical science | This study was excluded because it did not provide the exposure on Tg/glucose or Tg/HDL index values. |
| 699 | A common variation in the caveolin 1 gene is associated with high serum triglycerides and metabolic syndrome in an admixed Latin American population | 2018 | Metabolic Syndrome and Related Disorders | This study was excluded because it did not provide the exposure on Tg/glucose or Tg/HDL index values. |
| 700 | The triglycerides and glucose index is associated with cardiovascular risk factors in metabolically obese normal-weight subjects | 2020 | Journal of endocrinological investigation | This study was excluded because it did not provide the exposure on Tg/glucose or Tg/HDL index values. |
| 701 | PAI-1 produced ex vivo by human adipose tissue is relevant to PAI-1 blood level | 1999 | Arteriosclerosis, Thrombosis, and Vascular Biology | This study was excluded because it did not provide the exposure on Tg/glucose or Tg/HDL index values. |
| 702 | Metabolic syndrome and physical fitness in a sample of Azorean adolescents | 1999 | Metab syndr relat disord | This study was excluded because it did not provide the exposure on Tg/glucose or Tg/HDL index values. |
| 703 | Leptin and metabolic syndrome in obese and non-obese children | 2002 | Hormone and Metabolic Research | This study was excluded because it did not provide the exposure on Tg/glucose or Tg/HDL index values. |
| 704 | Dietary glycemic index, glycemic load and metabolic profile in children with phenylketonuria | 2017 | Nutrition, Metabolism and Cardiovascular Diseases | This study was excluded because it did not provide the exposure on Tg/glucose or Tg/HDL index values. |
| 705 | Homeostasis model assessment of insulin resistance*body mass index interactions at ages 9 to 10 years predict metabolic syndrome risk factor aggregate score at ages 18 to 19 years: a 10-year prospective study of black and white girls | 2009 | Metabolism: Clinical and Experimental | This study was excluded because it did not provide the exposure on Tg/glucose or Tg/HDL index values. |
| 706 | Childhood Predictors of Adult Type 2 Diabetes at 9- and 26-Year Follow-ups | 2010 | Archives of pediatrics & adolescent medicine | This study was excluded because it did not provide the exposure on Tg/glucose or Tg/HDL index values. |
| 707 | Hyperinsulmemia and metabolic syndrome at mean age of 10 years in black and white schoolgirls and development of impaired fasting glucose and type 2 diabetes mellitus by mean age of 24 years | 2011 | Metabolism-clinical and experimental | This study was excluded because it did not provide the exposure on Tg/glucose or Tg/HDL index values. |
| 708 | Lower soluble receptor for advanced glycation endproducts in adolescents with obesity from guanajuato: Associations with insulin resistance dyslipoproteinemia | 2017 | Endocrine practice | This study was excluded because it did not provide the exposure on Tg/glucose or Tg/HDL index values. |
| 709 | Cardiometabolic risk markers are associated with soluble receptor for advanced glycation end-products in adolescents with obesity | 2017 | Endocrine reviews | This study was excluded because it did not provide outcomes on the diagnostic performance of Tg/glucose or Tg/HDL index. |
| 710 | Triglycerides/HDL-cholesterol ratio in a population of adolescent overweight/obese (OW/OB): A new diagnostic marker of insulin resistance (IR) and metabolic syndrome (MS)? | 2014 | Obesity facts | This study was excluded because it did not provide outcomes on the diagnostic performance of Tg/glucose or Tg/HDL index. |
| 711 | Early testing of insulin resistance: a tale of two lipid ratios in a group of 5th graders screened by the Coronary Artery Risk Detection in Appalachian Communities Project (CARDIAC Project) | 2019 | World Journal of Pediatrics | This study was excluded because it did not provide outcomes on the diagnostic performance of Tg/glucose or Tg/HDL index. |
| 712 | Zinc and Selenium Co-supplementation Reduces Some Lipid Peroxidation and Angiogenesis Markers in a Rat Model of NAFLD-Fed High Fat Diet | 2018 | Biological trace element research | This study was excluded because it did not provide outcomes on the diagnostic performance of Tg/glucose or Tg/HDL index. |
| 713 | Predominant factors for diagnosis of metabolic syndrome in children and teenagers: a Systematic review | 2015 | Rbone-revista brasileira de obesidade nutricao e emagrecimento | This study was excluded because it did not provide outcomes on the diagnostic performance of Tg/glucose or Tg/HDL index. |
| 714 | Investigation of ischemia modified albumin and coenzyme Q10 levels in obese children with metabolic syndrome | 2016 | Turkish Journal of Biochemistry | This study was excluded because it did not provide outcomes on the diagnostic performance of Tg/glucose or Tg/HDL index. |
| 715 | Cardio-metabolic parameters are associated with genetic admixture estimates in a pediatric population from Colombia | 2016 | Bmc genetics | This study was excluded because it did not provide outcomes on the diagnostic performance of Tg/glucose or Tg/HDL index. |
| 716 | Plasma triglyceride/HDL-cholesterol ratio, insulin resistance, and cardiometabolic risk in young adults | 2013 | Journal of Lipid Research | This study was excluded because it did not provide outcomes on the diagnostic performance of Tg/glucose or Tg/HDL index. |
| 717 | The glucose response to an oral fat tolerance test in young men with a paternal history of premature myocardial infarction: possible early indication of insulin resistance. The EARS 2 study | 2005 | Annals of clinical biochemistry | This study was excluded because it did not provide outcomes on the diagnostic performance of Tg/glucose or Tg/HDL index. |
| 718 | Cardiometabolic risk factors as apolipoprotein B, triglyceride/HDL-cholesterol ratio and C-reactive protein, in adolescents with and without obesity: cross-sectional study in middle class suburban children | 2011 | Pediatric diabetes | This study was excluded because it did not provide outcomes on the diagnostic performance of Tg/glucose or Tg/HDL index. |
| 719 | Laparoscopic Adjustable Gastric Banding for Morbidly Obese Adolescents Affects Android Fat Loss, Resolution of Comorbidities, and Improved Metabolic Status | 2009 | Journal of the american college of surgeons | This study was excluded because it did not provide outcomes on the diagnostic performance of Tg/glucose or Tg/HDL index. |
| 720 | Elevated triglycerides are associated with decreased executive function among adolescents with bipolar disorder | 2016 | Acta psychiatrica scandinavica | This study was excluded because it did not provide outcomes on the diagnostic performance of Tg/glucose or Tg/HDL index. |
| 721 | Relationship Between Body Composition Parameters and Metabolic Syndrome in Young Thai Adults | 2014 | Journal of clinical research in pediatric endocrinology | This study was excluded because it did not provide outcomes on the diagnostic performance of Tg/glucose or Tg/HDL index. |
| 722 | Metabolic syndrome and insulin resistance in obese pre-pubertal children in Lebanon: A primary health concern | 2010 | Obesity reviews | This study was excluded because it did not provide outcomes on the diagnostic performance of Tg/glucose or Tg/HDL index. |
| 723 | Short-term effect of metformin treatment coupled with a low carbohydrate diet in non-diabetic overweight and obese adolescents | 2017 | Endocrine reviews | This study was excluded because it did not provide outcomes on the diagnostic performance of Tg/glucose or Tg/HDL index. |
| 724 | Relationship between ANGPTL3 and VO2max, body composition and markers of metabolic syndrome and effect of interval training on these variables in overweight and obese women | 2021 | Journal of Mazandaran University of Medical Sciences | This study was excluded because it did not provide outcomes on the diagnostic performance of Tg/glucose or Tg/HDL index. |
| 725 | Sex differences in biomarkers associated with insulin resistance in obese adolescents: Metabolomic profiling and principal components analysis | 2014 | Journal of Clinical Endocrinology and Metabolism | This study was excluded because it did not provide outcomes on the diagnostic performance of Tg/glucose or Tg/HDL index. |
| 726 | A 24-Week, Multicenter, Open-Label, Randomized Study to Compare Changes in Glucose Metabolism in Patients With Schizophrenia Receiving Treatment With Olanzapine, Quetiapine, or Risperidone | 2009 | Journal of clinical psychiatry | This study was excluded because it did not provide outcomes on the diagnostic performance of Tg/glucose or Tg/HDL index. |
| 727 | Changes in risk variables of metabolic syndrome since childhood in pre-diabetic and type 2 diabetic subjects | 2008 | Diabetes care | This study was excluded because it did not provide outcomes on the diagnostic performance of Tg/glucose or Tg/HDL index. |
| 728 | Changes in Risk Variables of Metabolic Syndrome ince Childhood in Prediabetic and Type 2 Diabetic Subjects: The Bogalusa Heart Study | 2008 | Circulation | This study was excluded because it did not provide outcomes on the diagnostic performance of Tg/glucose or Tg/HDL index. |
| 729 | Changes in risk variables of metabolic syndrome since childhood in pre-diabetic and type 2 diabetic subjects: the Bogalusa Heart Study | 2008 | Diabetes care | This study was excluded because it did not provide outcomes on the diagnostic performance of Tg/glucose or Tg/HDL index. |
| 730 | Lipid-lowering efficacy and safety after switching to atazanavir-ritonavir-based highly active Antiretroviral therapy in patients with human immunodeficiency virus | 2008 | Pharmacotherapy | This study was excluded because it did not provide outcomes on the diagnostic performance of Tg/glucose or Tg/HDL index. |
| 731 | Serum resistin positively correlates with serum lipids, but not with insulin resistance, in first-degree relatives of type-2 diabetes patients: an observational study in China | 2017 | Medicine | This study was excluded because it did not provide outcomes on the diagnostic performance of Tg/glucose or Tg/HDL index. |
| 732 | Severity of liver injury and cardiovascular risk in children with nonalcoholic fatty liver disease | 2009 | Gastroenterology | This study was excluded because it did not provide outcomes on the diagnostic performance of Tg/glucose or Tg/HDL index. |
| 733 | Severity of liver injury and atherogenic lipid profile in children with nonalcoholic fatty liver disease | 2010 | Pediatric research | This study was excluded because it did not provide outcomes on the diagnostic performance of Tg/glucose or Tg/HDL index. |
| 734 | Assessment of the correlation between the atherogenic index of plasma and cardiometabolic risk factors in children and adolescents: might it be superior to the TG/HDL-C ratio? | 2017 | Journal of pediatric endocrinology & metabolism | This study was excluded because it did not provide outcomes on the diagnostic performance of Tg/glucose or Tg/HDL index. |
| 735 | Longitudinal Study on the Lifestyle and Health of University Students (ELESEU): design, methodological procedures, and preliminary results | 2018 | Cad saude publica | This study was excluded because it did not provide outcomes on the diagnostic performance of Tg/glucose or Tg/HDL index. |
| 736 | Triglyceride glucose (TYG) index as a surrogate measure of insulin sensitivity in obese adolescents | 2015 | Endocrine reviews | This study was excluded because it did not provide outcomes on the diagnostic performance of Tg/glucose or Tg/HDL index. |
| 737 | Plasma IL-1 Receptor Antagonist Concentration Has an Inverse Association With Birth Weight in Prepubertal Children | 2018 | Journal of the endocrine society | This study was excluded because it did not provide outcomes on the diagnostic performance of Tg/glucose or Tg/HDL index. |
| 738 | Baseline metabolic disturbances and the twenty-five years risk of incident cancer in a Mediterranean population | 2016 | Nutrition metabolism and cardiovascular diseases | This study was excluded because it did not provide outcomes on the diagnostic performance of Tg/glucose or Tg/HDL index. |
| 739 | Vitamin D deficiency and insulin resistance in obese African-American adolescents | 2011 | Journal of Pediatric Endocrinology and Metabolism | This study was excluded because it did not provide outcomes on the diagnostic performance of Tg/glucose or Tg/HDL index. |
| 740 | Metabolic syndrome from adolescence to early adulthood: effect of infancy-onset dietary counseling of low saturated fat: the Special Turku Coronary Risk Factor Intervention Project (STRIP) | 2015 | Circulation | This study was excluded because it did not provide outcomes on the diagnostic performance of Tg/glucose or Tg/HDL index. |
| 741 | TG: HDL-C ratio is a good marker to identify children affected by obesity with increased cardiometabolic risk and insulin resistance | 2019 | International Journal of Endocrinology | This study was excluded because it did not provide outcomes on the diagnostic performance of Tg/glucose or Tg/HDL index. |
| 742 | Does cardiorespiratory fitness attenuate the adverse effects of severe/morbid obesity on cardiometabolic risk and insulin resistance in children? A pooled analysis | 2017 | Diabetes care | This study was excluded because it did not provide outcomes on the diagnostic performance of Tg/glucose or Tg/HDL index. |
| 743 | The visceral adiposity index as a predictor of insulin resistance in young women with polycystic ovary syndrome | 2013 | Obesity | This study was excluded because it did not provide outcomes on the diagnostic performance of Tg/glucose or Tg/HDL index. |
| 744 | Relationship between serum adiponectin level and lipid composition in each lipoprotein fraction in adolescent children | 2006 | Atherosclerosis | This study was excluded because it did not provide outcomes on the diagnostic performance of Tg/glucose or Tg/HDL index. |
| 745 | Continuous metabolic syndrome risk score, body mass index percentile, and leisure time physical activity in American children | 2010 | J clin hypertens (greenwich) | This study was excluded because it did not provide outcomes on the diagnostic performance of Tg/glucose or Tg/HDL index. |
| 746 | Validity of a continuous metabolic risk score as an index for modeling metabolic syndrome in adolescents | 2017 | Ann epidemiol | This study was excluded because it did not provide outcomes on the diagnostic performance of Tg/glucose or Tg/HDL index. |
| 747 | Oxidized low-density lipoprotein levels and carotid intima-media thickness as markers of early atherosclerosis in prepubertal obese children | 2013 | Journal of pediatric endocrinology & metabolism | This study was excluded because it did not provide outcomes on the diagnostic performance of Tg/glucose or Tg/HDL index. |
| 748 | Effect of a high-fructose diet on glucose tolerance, plasma lipid and hemorheological parameters during oral contraceptive administration in female rats | 2013 | Clinical hemorheology and microcirculation | This study was excluded because it did not provide outcomes on the diagnostic performance of Tg/glucose or Tg/HDL index. |
| 749 | Risk Factors for Chronic Diseases and Multimorbidity in a Primary Care Context of Central Argentina: A Web-Based Interactive and Cross-Sectional Study | 2017 | Int j environ res public health | This study was excluded because it did not provide outcomes on the diagnostic performance of Tg/glucose or Tg/HDL index. |
| 750 | Effect of brazil nut consumption and hypocaloric diet on the metabolic profile and skin nutritive microcirculatory patterns in obese female adolescents | 2009 | Journal of Diabetes | This study was excluded because it did not provide outcomes on the diagnostic performance of Tg/glucose or Tg/HDL index. |
| 751 | Metabolic Profile in Patients with Mild Obstructive Sleep Apnea | 2018 | Metabolic syndrome and related disorders | This study was excluded because it did not provide outcomes on the diagnostic performance of Tg/glucose or Tg/HDL index. |
| 752 | The triglyceride to HDL ratio and its relationship to insulin resistance in pre- and postpubertal children: Observation from the wausau SCHOOL project | 2012 | Cholesterol | This study was excluded because it did not provide outcomes on the diagnostic performance of Tg/glucose or Tg/HDL index. |
| 753 | The importance of liver ultarsound scores in nonalcoholic fatty liver disease in Egyptian obese children and adolescent | 2016 | Pediatric diabetes | This study was excluded because it did not provide outcomes on the diagnostic performance of Tg/glucose or Tg/HDL index. |
| 754 | The prevalence of metabolic syndrome and its components among overweight and obese Nigerian adolescents and young adults | 2017 | Nigerian journal of clinical practice | This study was excluded because it did not provide outcomes on the diagnostic performance of Tg/glucose or Tg/HDL index. |
| 755 | High-density lipoprotein apolipoprotein A-I kinetics: comparison of radioactive and stable isotope studies | 2006 | European journal of clinical investigation | This study was excluded because it did not provide outcomes on the diagnostic performance of Tg/glucose or Tg/HDL index. |
| 756 | Design of FitFor2 study: The effects of an exercise program on insulin sensitivity and plasma glucose levels in pregnant women at high risk for gestational diabetes | 2009 | BMC Pregnancy and Childbirth | This study was excluded because it did not provide outcomes on the diagnostic performance of Tg/glucose or Tg/HDL index. |
| 757 | Acanthosis nigricans as a clinical marker for insulin resistance in obese children | 2010 | Journal of Pediatric Gastroenterology and Nutrition | This study was excluded because it did not provide outcomes on the diagnostic performance of Tg/glucose or Tg/HDL index. |
| 758 | The effects of 'testosterone undecanoate' used for the treatment of delayet puberty and hypogonadism on serum glucose, insulin and lipid levels | 1997 | Istanbul tip fakultesi mecmuasi | This study was excluded because it did not provide outcomes on the diagnostic performance of Tg/glucose or Tg/HDL index. |
| 759 | Investigation of the prevalence of cardiovascular risk factors in obese patients diagnosed with metabolic syndrome in childhood and examination of left ventricular function by echocardiography | 2021 | Journal of Pediatric Endocrinology and Metabolism | This study was excluded because it did not provide outcomes on the diagnostic performance of Tg/glucose or Tg/HDL index. |
| 760 | Association of serum triglyceride-to-HDL cholesterol ratio with carotid artery intima-media thickness, insulin resistance and nonalcoholic fatty liver disease in children and adolescents | 2014 | Nutrition, Metabolism and Cardiovascular Diseases | This study was excluded because it did not provide outcomes on the diagnostic performance of Tg/glucose or Tg/HDL index. |
| 761 | Determinants of insulin resistance and nonalcoholic fatty liver disease (NAFLD) in children | 2009 | Journal of Pediatric Gastroenterology and Nutrition | This study was excluded because it did not provide outcomes on the diagnostic performance of Tg/glucose or Tg/HDL index. |
| 762 | Association of RANKL-OPG ratio with left ventricular hyperthrophy in obese children and adolescents | 2017 | Journal of Pediatric Gastroenterology and Nutrition | This study was excluded because it did not provide outcomes on the diagnostic performance of Tg/glucose or Tg/HDL index. |
| 763 | Metabolic syndrome in children and adolescents from a semirural city in southern Brazil | 2015 | Clinical Chemistry and Laboratory Medicine | This study was excluded because it did not provide outcomes on the diagnostic performance of Tg/glucose or Tg/HDL index. |
| 764 | Evaluation of metabolic syndrome after hematopoietic stem cell transplantation in children and adolescents | 2012 | Pediatr blood cancer | This study was excluded because it did not provide outcomes on the diagnostic performance of Tg/glucose or Tg/HDL index. |
| 765 | The Short-Term Effects of Risperidone-Induced Hyperprolactinemia on Lipid Metabolism in Drug-Naive Children and Adolescents | 2015 | Psychiatry investigation | This study was excluded because it did not provide outcomes on the diagnostic performance of Tg/glucose or Tg/HDL index. |
| 766 | The short‑term effects of risperidone‑induced hyperprolactinemia on lipid metabolism in drug-naive children and adolescents | 2015 | Psychiatry investigation | This study was excluded because it did not provide outcomes on the diagnostic performance of Tg/glucose or Tg/HDL index. |
| 767 | Leptin and the metabolic syndrome in Korean adolescents: Factor analysis | 2004 | Pediatrics international | This study was excluded because it did not provide outcomes on the diagnostic performance of Tg/glucose or Tg/HDL index. |
| 768 | Familial aggregation of the metabolic syndrome in Korean families with adolescents | 2006 | Atherosclerosis | This study was excluded because it did not provide outcomes on the diagnostic performance of Tg/glucose or Tg/HDL index. |
| 769 | Association between the triglyceride to high-density lipoprotein cholesterol ratio and insulin resistance in Korean adolescents: A nationwide population-based study | 2016 | Journal of Pediatric Endocrinology and Metabolism | This study was excluded because it did not provide outcomes on the diagnostic performance of Tg/glucose or Tg/HDL index. |
| 770 | The effect of exercise intervention program on the insulin resistance and the atherogenic index of the obese children | 2014 | The Korean Society of Sports Science | This study was excluded because it did not provide outcomes on the diagnostic performance of Tg/glucose or Tg/HDL index. |
| 771 | Free triiodothyronine/free thyroxine ratio rather than thyrotropin is more associated with metabolic parameters in healthy euthyroid adult subjects | 2017 | Clinical endocrinology | This study was excluded because it did not provide outcomes on the diagnostic performance of Tg/glucose or Tg/HDL index. |
| 772 | The Effect of Low Glycemic Index and Glycemic Load Diets on Hepatic Fat Mass, Insulin Resistance, and Blood Lipid Panels in Individuals with Nonalcoholic Fatty Liver Disease | 2019 | Metabolic syndrome and related disorders | This study was excluded because it did not provide outcomes on the diagnostic performance of Tg/glucose or Tg/HDL index. |
| 773 | Aspectos epidemiológicos de la prediabetes, diagnóstico y clasificación | 2011 | Revista Cubana de endocrinologã­a | This study was excluded because it did not provide outcomes on the diagnostic performance of Tg/glucose or Tg/HDL index. |
| 774 | Association of UCP1, UCP2 and UCP3 gene polymorphisms with cardiovascular disease risk factors in European adolescents: the HELENA study | 2020 | Pediatric research | This study was excluded because it did not provide outcomes on the diagnostic performance of Tg/glucose or Tg/HDL index. |
| 775 | Taste perception in massively obese and in non-obese adolescents | 2007 | International Journal of Pediatric Obesity | This study was excluded because it did not provide outcomes on the diagnostic performance of Tg/glucose or Tg/HDL index. |
| 776 | The incidence of metabolic syndrome in a group of obese children in Czech Republic | 2014 | Atherosclerosis | This study was excluded because it did not provide outcomes on the diagnostic performance of Tg/glucose or Tg/HDL index. |
| 777 | Insulin resistance Type A and short 5th metacarpals | 2003 | Diabetic medicine | This study was excluded because it did not provide outcomes on the diagnostic performance of Tg/glucose or Tg/HDL index. |
| 778 | Regional body fat and metabolic complications in children with familial partial lipodystrophy, dunnigan variety (FPLD2) | 2017 | Hormone Research in Paediatrics | This study was excluded because it did not provide outcomes on the diagnostic performance of Tg/glucose or Tg/HDL index. |
| 779 | Worsening hypertriglyceridemia with oral contraceptive pills in an adolescent with HIV-associated lipodystrophy: a case report and review of the literature | 2014 | Journal of pediatric endocrinology & metabolism | This study was excluded because it did not provide outcomes on the diagnostic performance of Tg/glucose or Tg/HDL index. |
| 780 | Regional Body Fat Changes and Metabolic Complications in Children With Dunnigan Lipodystrophy-Causing LMNA Variants | 2019 | Journal of clinical endocrinology & metabolism | This study was excluded because it did not provide outcomes on the diagnostic performance of Tg/glucose or Tg/HDL index. |
| 781 | Modification and validation of the triglyceride-to-HDL cholesterol ratio as a surrogate of insulin sensitivity in white juveniles and adults without diabetes mellitus: The single point insulin sensitivity estimator (SPISE) | 2016 | Clinical chemistry | This study was excluded because it did not provide outcomes on the diagnostic performance of Tg/glucose or Tg/HDL index. |
| 782 | Use of rosiglitazone in women with polycystic ovarian syndrome | 2005 | Akusherstvo i ginekologiia | This study was excluded because it did not provide outcomes on the diagnostic performance of Tg/glucose or Tg/HDL index. |
| 783 | Association of common variants in JAK2 gene with reduced risk of metabolic syndrome and related disorders | 2011 | Bmc medical genetics | This study was excluded because it did not provide outcomes on the diagnostic performance of Tg/glucose or Tg/HDL index. |
| 784 | Pediatric obesity and associated cardiometabolic risk factors | 2014 | Revista Cubana de Pediatria | This study was excluded because it did not provide outcomes on the diagnostic performance of Tg/glucose or Tg/HDL index. |
| 785 | Microbiota intestinal y enfermedad celíaca. Parte 1: Actualización | 2016 | Revista Cubana de pediatrã­a | This study was excluded because it did not provide outcomes on the diagnostic performance of Tg/glucose or Tg/HDL index. |
| 786 | Waist circumference shows the highest predictive value for metabolic syndrome, and waist-to-hip ratio for its components, in Spanish adolescents | 2017 | Nutrition research | This study was excluded because it did not provide outcomes on the diagnostic performance of Tg/glucose or Tg/HDL index. |
| 787 | Associations Between Circulating N-terminal pro-Brain Natriuretic Peptide (NT-proBNP) and Adiponectin Concentrations Depend on Obesity Level in Female Adolescents: Gender Dimorphic Findings | 2009 | Hormone and metabolic research | This study was excluded because it did not provide outcomes on the diagnostic performance of Tg/glucose or Tg/HDL index. |
| 788 | Estimated insulin sensitivity, cardiovascular risk, and hepatic steatosis after 12 years from the onset of T1D | 2002 | Diabetes-metabolism research and reviews | This study was excluded because it did not provide outcomes on the diagnostic performance of Tg/glucose or Tg/HDL index. |
| 789 | Estimated insulin sensitivity, cardiovascular risk, and hepatic steatosis after 12 years from the onset of T1D | 2021 | Diabetes/Metabolism Research and Reviews | This study was excluded because it did not provide outcomes on the diagnostic performance of Tg/glucose or Tg/HDL index. |
| 790 | Does insulin resistance, visceral adiposity, or a sex hormone alteration underlie the metabolic syndrome? Studies in women | 2008 | Metabolism-clinical and experimental | This study was excluded because it did not provide outcomes on the diagnostic performance of Tg/glucose or Tg/HDL index. |
| 791 | Insulin resistance and the metabolic syndrome components in obese children and adolescents | 2015 | Revista Cubana de Pediatria | This study was excluded because it did not provide outcomes on the diagnostic performance of Tg/glucose or Tg/HDL index. |
| 792 | Effects of Vitamin D on Blood Pressure and Cardiovascular Risk Factors A Randomized Controlled Trial | 2015 | Hypertension | This study was excluded because it did not provide outcomes on the diagnostic performance of Tg/glucose or Tg/HDL index. |
| 793 | Prevalence of overweight, obesity and metabolic syndrome components in children, adolescents and young adults with type 1 diabetes mellitus | 2015 | Diabetes-metabolism research and reviews | This study was excluded because it did not provide outcomes on the diagnostic performance of Tg/glucose or Tg/HDL index. |
| 794 | Data-driven dietary patterns at 7 year-old and their association with cardiometabolic health at 10 year-old | 2019 | Obesity facts | This study was excluded because it did not provide outcomes on the diagnostic performance of Tg/glucose or Tg/HDL index. |
| 795 | Optimal cut-off points of fasting and post-glucose stimulus surrogates of insulin resistance as predictors of metabolic syndrome in adolescents according to several definitions | 2018 | JCRPE Journal of Clinical Research in Pediatric Endocrinology | This study was excluded because it did not provide outcomes on the diagnostic performance of Tg/glucose or Tg/HDL index. |
| 796 | Prevalence and characteristics of the metabolically healthy obese phenotype in children and adolescents in a Mexican state | 2020 | Endocrinologia, Diabetes y Nutricion | This study was excluded because it did not provide outcomes on the diagnostic performance of Tg/glucose or Tg/HDL index. |
| 797 | Role of urinary NGAL and KIM-1 as biomarkers of early kidney injury in obese prepubertal children | 2020 | Journal of Pediatric Endocrinology and Metabolism | This study was excluded because it did not provide outcomes on the diagnostic performance of Tg/glucose or Tg/HDL index. |
| 798 | Early Changes in the Components of the Metabolic Syndrome in a Group of Smokers After Tobacco Cessation | 2014 | Metabolic syndrome and related disorders | This study was excluded because it did not provide outcomes on the diagnostic performance of Tg/glucose or Tg/HDL index. |
| 799 | Hyperuricemia and its association with the presence of metabolic syndrome among indonesian obese adolescents | 2016 | Pakistan Journal of Nutrition | This study was excluded because it did not provide outcomes on the diagnostic performance of Tg/glucose or Tg/HDL index. |
| 800 | Low HDL-cholesterol among normal weight, normoglycemic offspring of individuals with type 2 diabetes mellitus | 2011 | Hormones | This study was excluded because it did not provide outcomes on the diagnostic performance of Tg/glucose or Tg/HDL index. |
| 801 | Obesity and Metabolic Abnormalities in Offspring of Subjects with Diabetes Mellitus | 2010 | Diabetes technology & therapeutics | This study was excluded because it did not provide outcomes on the diagnostic performance of Tg/glucose or Tg/HDL index. |
| 802 | The triglyceride/HDL-cholesterol ratio as a marker of cardiovascular risk in obese children | 2008 | Pediatr diabetes | This study was excluded because it did not provide outcomes on the diagnostic performance of Tg/glucose or Tg/HDL index. |
| 803 | Correlates of Age Onset of Type 2 Diabetes Among Relatively Young Black and White Adults in a Community The Bogalusa Heart Study | 2012 | Diabetes care | This study was excluded because it did not provide outcomes on the diagnostic performance of Tg/glucose or Tg/HDL index. |
| 804 | Relation of blood pressure and body mass index during childhood to cardiovascular risk factor levels in young adults | 2009 | Journal of Hypertension | This study was excluded because it did not provide outcomes on the diagnostic performance of Tg/glucose or Tg/HDL index. |
| 805 | The relationship between hyperthyrotropinemia and metabolic and cardiovascular risk factors in a large group of overweight and obese children and adolescents | 2017 | Journal of endocrinological investigation | This study was excluded because it did not provide outcomes on the diagnostic performance of Tg/glucose or Tg/HDL index. |
| 806 | Hostility predicts metabolic syndrome risk factors in children and adolescents | 2003 | Health psychology | This study was excluded because it did not provide outcomes on the diagnostic performance of Tg/glucose or Tg/HDL index. |
| 807 | Effect of Lifestyle Modification and Metformin Therapy on Emerging Cardiovascular Risk Factors in Overweight Indian Women with Polycystic Ovary Syndrome | 2012 | Metabolic syndrome and related disorders | This study was excluded because it did not provide outcomes on the diagnostic performance of Tg/glucose or Tg/HDL index. |
| 808 | Association and interaction of the FTO rs1421085 with overweight/obesity in a sample of Pakistani individuals | 2020 | Eating and weight disorders-studies on anorexia bulimia and obesity | This study was excluded because it did not provide outcomes on the diagnostic performance of Tg/glucose or Tg/HDL index. |
| 809 | Schoolchildren born VLBW or VLGA show height-related changes in body composition and muscle function but no evidence of metabolic syndrome risk factors. Results from the NEOLONG study | 2016 | Journal of pediatric endocrinology & metabolism | This study was excluded because it did not provide outcomes on the diagnostic performance of Tg/glucose or Tg/HDL index. |
| 810 | Metabolic syndrome prevalence in Chilean children and adolescent with family history of chronic non-communicable diseases | 2012 | Archivos Latinoamericanos de Nutricion | This study was excluded because it did not provide outcomes on the diagnostic performance of Tg/glucose or Tg/HDL index. |
| 811 | Association of blood pressure and metabolic syndrome components with magnesium levels in drinking water in some Serbian municipalities | 2012 | Journal of water and health | This study was excluded because it did not provide outcomes on the diagnostic performance of Tg/glucose or Tg/HDL index. |
| 812 | Association of Baseline Characteristics With Insulin Sensitivity and Î²-Cell Function in the Glycemia Reduction Approaches in Diabetes: A Comparative Effectiveness (GRADE) Study Cohort | 2021 | Diabetes care | This study was excluded because it did not provide outcomes on the diagnostic performance of Tg/glucose or Tg/HDL index. |
| 813 | Association of Baseline Characteristics With Insulin Sensitivity and beta-Cell Function in the Glycemia Reduction Approaches in Diabetes: A Comparative Effectiveness (GRADE) Study Cohort | 2021 | Diabetes care | This study was excluded because it did not provide outcomes on the diagnostic performance of Tg/glucose or Tg/HDL index. |
| 814 | Metabolic Syndrome in Hispanic Youth: Results from the Hispanic Community Children's Health Study/Study of Latino Youth | 2017 | Metabolic syndrome and related disorders | This study was excluded because it did not provide outcomes on the diagnostic performance of Tg/glucose or Tg/HDL index. |
| 815 | Cardiovascular risk factors in overweight German children and adolescents: Relation to gender, age and degree of overweight | 2005 | Nutrition metabolism and cardiovascular diseases | This study was excluded because it did not provide outcomes on the diagnostic performance of Tg/glucose or Tg/HDL index. |
| 816 | Comparison of metabolic syndrome prevalence using eight different definitions: A critical approach | 2007 | Archives of Disease in Childhood | This study was excluded because it did not provide outcomes on the diagnostic performance of Tg/glucose or Tg/HDL index. |
| 817 | Changes of cardiovascular risk factors in obese children effects of inpatient and outpatient interventions | 2006 | Journal of pediatric gastroenterology and nutrition | This study was excluded because it did not provide outcomes on the diagnostic performance of Tg/glucose or Tg/HDL index. |
| 818 | Insulin sensitivity indices of glucose and free fatty acid metabolism in obese children and adolescents in relation to serum lipids | 2005 | Metabolism: Clinical and Experimental | This study was excluded because it did not provide outcomes on the diagnostic performance of Tg/glucose or Tg/HDL index. |
| 819 | Longitudinal association between IGFBP-1 levels and parameters of the metabolic syndrome in obese children before and after weight loss | 2011 | International Journal of Pediatric Obesity | This study was excluded because it did not provide outcomes on the diagnostic performance of Tg/glucose or Tg/HDL index. |
| 820 | Which amount of BMI-SDS reduction is necessary to improve cardiovascular risk factors in overweight and obese children? | 2016 | Hormone Research in Paediatrics | This study was excluded because it did not provide outcomes on the diagnostic performance of Tg/glucose or Tg/HDL index. |
| 821 | Which amount of BMI-SDS reduction is necessary to improve cardiovascular risk factors in overweight children? | 2016 | Journal of Clinical Endocrinology and Metabolism | This study was excluded because it did not provide outcomes on the diagnostic performance of Tg/glucose or Tg/HDL index. |
| 822 | Association between IGFBP-1 levels, fatty liver disease and metabolic syndrome in obese children before and after weight loss | 2011 | Endocrine reviews | This study was excluded because it did not provide outcomes on the diagnostic performance of Tg/glucose or Tg/HDL index. |
| 823 | Relationships between cardiovascular risk profile, ultrasonographic measurement of intra-abdominal adipose tissue, and waist circumference in obese children | 2010 | Clinical nutrition | This study was excluded because it did not provide outcomes on the diagnostic performance of Tg/glucose or Tg/HDL index. |
| 824 | Sex differences in young adulthood metabolic syndrome and physical activity: The Fels longitudinal study | 2007 | American journal of human biology | This study was excluded because it did not provide outcomes on the diagnostic performance of Tg/glucose or Tg/HDL index. |
| 825 | Longitudinal Associations Between Metabolic Syndrome Components and Telomere Shortening | 2015 | Journal of clinical endocrinology & metabolism | This study was excluded because it did not provide outcomes on the diagnostic performance of Tg/glucose or Tg/HDL index. |
| 826 | Telomere Length as a Marker of Cellular Aging Is Associated With Prevalence and Progression of Metabolic Syndrome | 2014 | Journal of clinical endocrinology & metabolism | This study was excluded because it did not provide outcomes on the diagnostic performance of Tg/glucose or Tg/HDL index. |
| 827 | Comparison of Lipid Ratios to Identify Metabolic Syndrome | 2018 | Archives of iranian medicine | This study was excluded because it did not provide outcomes on the diagnostic performance of Tg/glucose or Tg/HDL index. |
| 828 | Size for gestational age at birth and cardiometabolic health in childhood | 2018 | American Journal of Obstetrics and Gynecology | This study was excluded because it did not provide outcomes on the diagnostic performance of Tg/glucose or Tg/HDL index. |
| 829 | Pregnancy-associated hypertension and offspring cardiometabolic health | 2018 | Obstetrics and Gynecology | This study was excluded because it did not provide outcomes on the diagnostic performance of Tg/glucose or Tg/HDL index. |
| 830 | A novel approach to childhood obesity: circulating chemokines and growth factors as biomarkers of insulin resistance | 2019 | Pediatric obesity | This study was excluded because it did not provide outcomes on the diagnostic performance of Tg/glucose or Tg/HDL index. |
| 831 | A combination of circulating chemokines as biomarkers of obesity-induced insulin resistance at puberty | 2021 | Pediatric obesity | This study was excluded because it did not provide outcomes on the diagnostic performance of Tg/glucose or Tg/HDL index. |
| 832 | Relationship between the triglyceride/high-density lipoprotein-cholesterol ratio, insulin resistance index and cardiometabolic risk factors in women with polycystic ovary syndrome | 2009 | Endocrinologia y Nutricion | This study was excluded because it did not provide outcomes on the diagnostic performance of Tg/glucose or Tg/HDL index. |
| 833 | Dietary treatment in phenylketonuria does not lead to increased risk of obesity or metabolic syndrome | 2012 | Mol genet metab | This study was excluded because it did not provide outcomes on the diagnostic performance of Tg/glucose or Tg/HDL index. |
| 834 | Cardiovascular risk factors, their associations and presence of metabolic syndrome in adolescents | 2009 | Jornal de pediatria | This study was excluded because it did not provide outcomes on the diagnostic performance of Tg/glucose or Tg/HDL index. |
| 835 | Prevalence of metabolic syndrome in children and adolecents with obesity and overweight in a childhood obesity clinic in Mexico | 2016 | Hormone Research in Paediatrics | This study was excluded because it did not provide outcomes on the diagnostic performance of Tg/glucose or Tg/HDL index. |
| 836 | Association of the Triglyceride/High-Density Lipoprotein Cholesterol Index with Insulin Resistance in a Pediatric Population in Northeast Mexico | 2020 | Metabolic Syndrome and Related Disorders | Included |
| 837 | Atherogenic indices and prehypertension in obese and non-obese children | 2013 | Diabetes & vascular disease research | This study was excluded because it did not provide outcomes on the diagnostic performance of Tg/glucose or Tg/HDL index. |
| 838 | The triglyceride and glucose index is useful for recognising insulin resistance in children | 2017 | Acta Paediatrica, International Journal of Paediatrics | Included |
| 839 | Higher D-lactate levels are associated with higher prevalence of small dense low-density lipoprotein in obese adolescents | 2018 | Clinical Chemistry and Laboratory Medicine | This study was excluded because it did not provide outcomes on the diagnostic performance of Tg/glucose or Tg/HDL index. |
| 840 | Insulin immunogenicity and its various clinical aspects | 1983 | Polski tygodnik lekarski (Warsaw, Poland : 1960) | This study was excluded because it did not provide outcomes on the diagnostic performance of Tg/glucose or Tg/HDL index. |
| 841 | A New Method for Screening Diagnosis of Insulin Resistance | 2015 | Bulletin of experimental biology and medicine | This study was excluded because it did not provide outcomes on the diagnostic performance of Tg/glucose or Tg/HDL index. |
| 842 | High sensitivity C-reactive protein concentrations, birthweight and cardiovascular risk markers in Brazilian children | 2013 | European Journal of Clinical Nutrition | This study was excluded because it did not provide outcomes on the diagnostic performance of Tg/glucose or Tg/HDL index. |
| 843 | Cortisol diurnal index stability predicts changes in metabolic syndrome symptoms over 2-years in adolescent girls | 2014 | Psychosomatic medicine | This study was excluded because it did not provide outcomes on the diagnostic performance of Tg/glucose or Tg/HDL index. |
| 844 | Prevalence of metabolic syndrome and associated risk factors in Ecuadorian university students | 2015 | Nutricion hospitalaria | This study was excluded because it did not provide outcomes on the diagnostic performance of Tg/glucose or Tg/HDL index. |
| 845 | Inflammatory cytokines and metabolic risk factors during growth and maturation: Influence of physical activity | 2010 | Med sport sci | This study was excluded because it did not provide outcomes on the diagnostic performance of Tg/glucose or Tg/HDL index. |
| 846 | Home-based physical activity positively affects cardiometabolic risk factors in children with and without prader-willi syndrome | 2017 | Hormone Research in Paediatrics | This study was excluded because it did not provide outcomes on the diagnostic performance of Tg/glucose or Tg/HDL index. |
| 847 | Influence of GH therapy on vaspin concentration and correlations of vaspin with selected parameters of carbohydrate and lipid metabolism in prepubertal non-obese children with growth hormone deficiency | 2017 | Hormone Research in Paediatrics | This study was excluded because it did not provide outcomes on the diagnostic performance of Tg/glucose or Tg/HDL index. |
| 848 | Traditional and novel cardiovascular risk factors in school-aged children: A call for the further development of public health strategies with emphasis on fitness | 2007 | Journal of Public Health | This study was excluded because it did not provide outcomes on the diagnostic performance of Tg/glucose or Tg/HDL index. |
| 849 | Mediterranean-style dietary pattern, reduced risk of metabolic syndrome traits, and incidence in the Framingham Offspring Cohort | 2009 | American Journal of Clinical Nutrition | This study was excluded because it did not provide outcomes on the diagnostic performance of Tg/glucose or Tg/HDL index. |
| 850 | Changes in leukocyte profile and C-reactive protein concentration in overweight and obese adolescents after reduction of body weight | 2019 | Central European Journal of Immunology | This study was excluded because it did not provide outcomes on the diagnostic performance of Tg/glucose or Tg/HDL index. |
| 851 | Association between serum C-peptide levels and the risk of cardiovascular disease in nondiabetic individuals: Data from the national health and nutrition examination survey, 1999-2004 | 2007 | Circulation | This study was excluded because it did not provide outcomes on the diagnostic performance of Tg/glucose or Tg/HDL index. |
| 852 | Waist Circumference Provides an Indication of Numerous Cardiometabolic Risk Factors in Adults With Cerebral Palsy | 2014 | Archives of physical medicine and rehabilitation | This study was excluded because it did not provide outcomes on the diagnostic performance of Tg/glucose or Tg/HDL index. |
| 853 | Adipose Tissue and Metabolic Alterations: Regional Differences in Fat Cell Size and Number Matter, But Differently: A Cross-Sectional Study | 2014 | Journal of clinical endocrinology & metabolism | This study was excluded because it did not provide outcomes on the diagnostic performance of Tg/glucose or Tg/HDL index. |
| 854 | Metabolic syndrome in patients with systemic lupus erythematosus from Southern Spain | 2008 | Lupus | This study was excluded because it did not provide outcomes on the diagnostic performance of Tg/glucose or Tg/HDL index. |
| 855 | Parental and offspring associations of the metabolic syndrome in the Fels Longitudinal Study | 2012 | American journal of clinical nutrition | This study was excluded because it did not provide outcomes on the diagnostic performance of Tg/glucose or Tg/HDL index. |
| 856 | The association of cardiorespiratory fitness to health independent of adiposity depends upon its expression | 2016 | Annals of human biology | This study was excluded because it did not provide outcomes on the diagnostic performance of Tg/glucose or Tg/HDL index. |
| 857 | Computational Methods Are Significant Determinants of the Associations and Definitions of Insulin Resistance Using the Homeostasis Model Assessment in Women of Reproductive Age | 2011 | Clinical chemistry | This study was excluded because it did not provide outcomes on the diagnostic performance of Tg/glucose or Tg/HDL index. |
| 858 | Comparison between BMI and Inverted BMI in Evaluating Metabolic Risk and Body Composition in Iranian Children | 2016 | International journal of pediatrics-mashhad | This study was excluded because it did not provide outcomes on the diagnostic performance of Tg/glucose or Tg/HDL index. |
| 859 | Use of the triglyceride/high-density lipoprotein cholesterol ratio to identify cardiometabolic risk: impact of obesity? | 2017 | Journal of investigative medicine | This study was excluded because it did not provide outcomes on the diagnostic performance of Tg/glucose or Tg/HDL index. |
| 860 | Identifying cardiovascular disease risk and outcome: use of the plasma triglyceride/high-density lipoprotein cholesterol concentration ratio versus metabolic syndrome criteria | 2013 | Journal of internal medicine | This study was excluded because it did not provide outcomes on the diagnostic performance of Tg/glucose or Tg/HDL index. |
| 861 | Use of the plasma triglyceride/high-density lipoprotein cholesterol ratio to identify cardiovascular disease in hypertensive subjects | 2014 | Journal of the American Society of Hypertension | This study was excluded because it did not provide outcomes on the diagnostic performance of Tg/glucose or Tg/HDL index. |
| 862 | Relation among the plasma triglyceride/high-density lipoprotein cholesterol concentration ratio, insulin resistance, and associated cardio-metabolic risk factors in men and women | 2012 | American Journal of Cardiology | This study was excluded because it did not provide outcomes on the diagnostic performance of Tg/glucose or Tg/HDL index. |
| 863 | Nocturnal but not Diurnal Hypertension Is Associated to Insulin Resistance Markers in Subjects With Normal or Mildly Elevated Office Blood Pressure | 2017 | American journal of hypertension | This study was excluded because it did not provide outcomes on the diagnostic performance of Tg/glucose or Tg/HDL index. |
| 864 | Prevalence of obesity and metabolic syndrome among adolescent girls in Rafsanjan, 2007 | 2007 | Iranian Journal of Diabetes and Lipid Disorders | This study was excluded because it did not provide outcomes on the diagnostic performance of Tg/glucose or Tg/HDL index. |
| 865 | Prevalence of obesity and metabolic syndrome in adolescent girls in South East of Iran | 2009 | Pakistan Journal of Medical Sciences | This study was excluded because it did not provide outcomes on the diagnostic performance of Tg/glucose or Tg/HDL index. |
| 866 | Muscle-to-Fat Ratio for Predicting Metabolic Syndrome Components in Children with Overweight and Obesity | 2021 | Childhood obesity (Print) | This study was excluded because it did not provide outcomes on the diagnostic performance of Tg/glucose or Tg/HDL index. |
| 867 | Cardiometabolic risk: Leg fat is protective during childhood | 2016 | Pediatric diabetes | This study was excluded because it did not provide outcomes on the diagnostic performance of Tg/glucose or Tg/HDL index. |
| 868 | Metabolically unhealthy obesity and visceral adiposity in children | 2016 | Obesity reviews | This study was excluded because it did not provide outcomes on the diagnostic performance of Tg/glucose or Tg/HDL index. |
| 869 | Association of nonalcoholic fatty liver disease with cardiovascular risk factors in obese adolescents: The role of interdisciplinary therapy | 2014 | Journal of Clinical Lipidology | This study was excluded because it did not provide outcomes on the diagnostic performance of Tg/glucose or Tg/HDL index. |
| 870 | The triglyceride / glucose index as an insulin resistance marker in the pediatric population and its relation to eating habits and physical activity | 2021 | Endocrinologia, Diabetes y Nutricion | Included |
| 871 | Diagnostic accuracy of the triglyceride-glucose index for gestational diabetes screening: a practical approach | 2020 | Gynecological endocrinology | This study was excluded because it did not provide outcomes on the diagnostic performance of Tg/glucose or Tg/HDL index. |
| 872 | Leg fat might be more protective than arm fat in relation to lipid profile | 2013 | European journal of nutrition | This study was excluded because it did not provide outcomes on the diagnostic performance of Tg/glucose or Tg/HDL index. |
| 873 | Relationship between DXA measured metrics of adiposity and glucose homeostasis | 2019 | Plos One | This study was excluded because it did not provide outcomes on the diagnostic performance of Tg/glucose or Tg/HDL index. |
| 874 | Effects of curcumin on cardiovascular risk factors in obese and overweight adolescent girls: a randomized clinical trial | 2019 | Sao paulo medical journal | This study was excluded because it did not provide outcomes on the diagnostic performance of Tg/glucose or Tg/HDL index. |
| 875 | A Comparison between BMI, Waist Circumference, and Waist-To-Height Ratio for Identifying Cardio-Metabolic Risk in Children and Adolescents | 2016 | Plos One | This study was excluded because it did not provide outcomes on the diagnostic performance of Tg/glucose or Tg/HDL index. |
| 876 | Metformin maintains the weight loss and metabolic benefits following rimonabant treatment in obese women with polycystic ovary syndrome (PCOS) | 2009 | Clinical endocrinology | This study was excluded because it did not provide outcomes on the diagnostic performance of Tg/glucose or Tg/HDL index. |
| 877 | Effects of prolonged sitting and physical activity on markers of cardiometabolic risk in healthy childrenandyouth: A pilot study | 2012 | Journal of Science and Medicine in Sport | This study was excluded because it did not provide outcomes on the diagnostic performance of Tg/glucose or Tg/HDL index. |
| 878 | Prolonged sitting and markers of cardiometabolic disease risk in children and youth: A randomized crossover study | 2013 | Metabolism: Clinical and Experimental | This study was excluded because it did not provide outcomes on the diagnostic performance of Tg/glucose or Tg/HDL index. |
| 879 | Biological and biochemical characteristics of a Mediterranean population with Gestational Diabetes Mellitus | 2016 | Journal of Perinatal Medicine | This study was excluded because it did not provide outcomes on the diagnostic performance of Tg/glucose or Tg/HDL index. |
| 880 | Short-Term Regulation of Lipocalin-2 but not RBP-4 During Oral Lipid Tolerance Test and Oral Glucose Tolerance Test | 2016 | Hormone and metabolic research | This study was excluded because it did not provide outcomes on the diagnostic performance of Tg/glucose or Tg/HDL index. |
| 881 | Metabolic Effects of Paliperidone Extended Release Versus Oral Olanzapine in Patients With Schizophrenia A Prospective, Randomized, Controlled Trial | 2012 | Journal of clinical psychopharmacology | This study was excluded because it did not provide outcomes on the diagnostic performance of Tg/glucose or Tg/HDL index. |
| 882 | Weight-basedteasing in youth: Associations with metabolic and inflammatory markers | 2021 | Pediatric obesity | This study was excluded because it did not provide outcomes on the diagnostic performance of Tg/glucose or Tg/HDL index. |
| 883 | Cardiometabolic risk factors in hypertensive children and adolescents: The PEP Family Heart Study | 2018 | European heart journal | This study was excluded because it did not provide outcomes on the diagnostic performance of Tg/glucose or Tg/HDL index. |
| 884 | Can skinfold thickness predict cardio-vascular risk factors in youths? | 2011 | Atherosclerosis supplements | This study was excluded because it did not provide outcomes on the diagnostic performance of Tg/glucose or Tg/HDL index. |
| 885 | Physical fitness as a mediator between objectively measured physical activity and clustered metabolic syndrome in children and adolescents: The UP&DOWN study | 2016 | Nutr metab cardiovasc dis | This study was excluded because it did not provide outcomes on the diagnostic performance of Tg/glucose or Tg/HDL index. |
| 886 | Apo-B/AI ratio identifies cardiovascular risk in childhood: the Australian Aboriginal Birth Cohort study | 2009 | Diab vasc dis res | This study was excluded because it did not provide outcomes on the diagnostic performance of Tg/glucose or Tg/HDL index. |
| 887 | Cardiorespiratory Fitness and Adiposity in Metabolically Healthy Overweight and Obese Youth | 2013 | Pediatrics | This study was excluded because it did not provide outcomes on the diagnostic performance of Tg/glucose or Tg/HDL index. |
| 888 | Validation of surrogate markers for metabolic syndrome and cardiometabolic risk factor clustering in children and adolescents: A nationwide population-based study | 2017 | Plos one | This study was excluded because it did not provide outcomes on the diagnostic performance of Tg/glucose or Tg/HDL index. |
| 889 | Correlation of P-wave dispersion with insulin sensitivity in obese adolescents | 2017 | Cardiology in the Young | This study was excluded because it did not provide outcomes on the diagnostic performance of Tg/glucose or Tg/HDL index. |
| 890 | Low 25-hydroxyvitamin D level is not an independent risk factor for hepatosteatosis in obese children | 2016 | Journal of pediatric endocrinology & metabolism | This study was excluded because it did not provide outcomes on the diagnostic performance of Tg/glucose or Tg/HDL index. |
| 891 | Association of polymorphism genes LPL, ADRB2, AGT and AGTR1 with risk of hyperinsulinism and insulin resistance in the Kazakh population | 2020 | Biomedical reports | This study was excluded because it did not provide outcomes on the diagnostic performance of Tg/glucose or Tg/HDL index. |
| 892 | Comparison of indices of insulin resistance with metabolic syndrome classifications to predict the development of impaired fasting glucose in overweight and obese subjects: A 3-year prospective study | 2009 | International Journal of Obesity | This study was excluded because it did not provide outcomes on the diagnostic performance of Tg/glucose or Tg/HDL index. |
| 893 | Independent and Interactive Associations of Fitness and Fatness With Changes in Cardiometabolic Risk in Children: A Longitudinal Analysis | 2020 | Front endocrinol (lausanne) | This study was excluded because it did not provide outcomes on the diagnostic performance of Tg/glucose or Tg/HDL index. |
| 894 | Identifying metabolic syndrome in African American children using fasting HOMA-IR in place of glucose | 2011 | Preventing chronic disease | This study was excluded because it did not provide outcomes on the diagnostic performance of Tg/glucose or Tg/HDL index. |
| 895 | GCKR Variants Increase Triglycerides While Protecting from Insulin Resistance in Chinese Children | 2013 | Plos ONE | This study was excluded because it did not provide the exposure on Tg/glucose or Tg/HDL index values. |
| 896 | Interdisciplinary therapy had positive effects on inflammatory state, mediated by leptin, adiponectin, and quality of diet in obese women | 2020 | Nutricion hospitalaria | This study was excluded because it did not provide the exposure on Tg/glucose or Tg/HDL index values. |
| 897 | Low-density lipoprotein particle size and its regulatory factors in school children | 2004 | Journal of Clinical Endocrinology and Metabolism | This study was excluded because it did not provide the exposure on Tg/glucose or Tg/HDL index values. |
| 898 | Metabolic syndrome as a predictor of type2 diabetes, and its clinical interpretations and usefulness | 2013 | Journal of diabetes investigation | This study was excluded because it did not provide the exposure on Tg/glucose or Tg/HDL index values. |
| 899 | Effect of Caffeine Co-Ingested with Carnitine on Weight, Body-Fat Percent, Serum Leptin and Lipid Profile Changes in Male Teen Soccer Players: a Randomized Clinical Trial | 2016 | International journal of pediatrics-mashhad | This study was excluded because it did not provide the exposure on Tg/glucose or Tg/HDL index values. |
| 900 | Levels Of Vitamin D And Cardiometabolic Risk Factors In Obesity Adolescents | 2019 | World of medicine and biology | This study was excluded because it did not provide the exposure on Tg/glucose or Tg/HDL index values. |
| 901 | Serum adipokines (adiponectin and resistin) correlation in developing gestational diabetes mellitus: pilot study | 2018 | Gynecological endocrinology | This study was excluded because it did not provide the exposure on Tg/glucose or Tg/HDL index values. |
| 902 | Contribution of clinical, metabolic, and genetic factors on hypertension in obese children and adolescents | 2011 | Journal of Pediatric Endocrinology and Metabolism | This study was excluded because it did not provide the exposure on Tg/glucose or Tg/HDL index values. |
| 903 | The triglyceride and glucose index is a useful biomarker to recognize glucose disorders in apparently healthy children and adolescents | 2020 | European journal of pediatrics | This study was excluded because it did not provide the exposure on Tg/glucose or Tg/HDL index values. |
| 904 | The correct formula for the triglycerides and glucose index | 2020 | European journal of pediatrics | This study was excluded because it did not provide the exposure on Tg/glucose or Tg/HDL index values. |
| 905 | The triglycerides and glucose index is associated with elevated blood pressure in apparently healthy children and adolescents | 2019 | European Journal of Pediatrics | This study was excluded because it did not provide the exposure on Tg/glucose or Tg/HDL index values. |
| 906 | The triglycerides and glucose index is associated with cardiovascular risk factors in normal-weight children and adolescents | 2017 | Pediatric research | This study was excluded because it did not provide the exposure on Tg/glucose or Tg/HDL index values. |
| 907 | The triglycerides and glucose index is strongly associated with hepatic steatosis in children with overweight or obesity | 2021 | European journal of pediatrics | This study was excluded because it did not provide the exposure on Tg/glucose or Tg/HDL index values. |
| 908 | The product of fasting glucose and triglycerides as surrogate for identifying insulin resistance in apparently healthy subjects | 2008 | Metabolic syndrome and related disorders | This study was excluded because it did not provide the exposure on Tg/glucose or Tg/HDL index values. |
| 909 | Media use trajectories and risk of metabolic syndrome in European children and adolescents: the IDEFICS/I.Family cohort | 2021 | The international journal of behavioral nutrition and physical activity | This study was excluded because it did not provide the exposure on Tg/glucose or Tg/HDL index values. |
| 910 | Insulin resistance syndrome in childhood: Associations of the euglycemic insulin clamp and fasting insulin with fatness and other risk factors | 2001 | Journal of Pediatrics | This study was excluded because it did not provide the exposure on Tg/glucose or Tg/HDL index values. |
| 911 | Influence of insulin resistance and body mass index at age 13 on systolic blood pressure, triglycerides, and high-density lipoprotein cholesterol at age 19 | 2006 | Hypertension | This study was excluded because it did not provide the exposure on Tg/glucose or Tg/HDL index values. |
| 912 | Relation of insulin resistance to blood pressure in childhood | 2002 | Journal of Hypertension | This study was excluded because it did not provide the exposure on Tg/glucose or Tg/HDL index values. |
| 913 | Relation of body mass index and insulin resistance to cardiovascular risk factors, inflammatory factors, and oxidative stress during adolescence | 2005 | Circulation | This study was excluded because it did not provide the exposure on Tg/glucose or Tg/HDL index values. |
| 914 | Childhood obesity: Contributing factors and consequences in Indian children | 2007 | Diabetes and Metabolic Syndrome: Clinical Research and Reviews | This study was excluded because it did not provide the exposure on Tg/glucose or Tg/HDL index values. |
| 915 | Secular Trends in Obesity, Regional Adiposity and Metabolic Parameters among Asian Indian Adolescents in North India: A Comparative Data Analysis of Two Selective Samples 5 Years Apart (2003,2008) | 2010 | Annals of nutrition and metabolism | This study was excluded because it did not provide the exposure on Tg/glucose or Tg/HDL index values. |
| 916 | Prevalence of insulin resistance and association with metabolic risk factors and food consumption in adolescents - Recife/Brazil | 2020 | Revista paulista de pediatria | This study was excluded because it did not provide the exposure on Tg/glucose or Tg/HDL index values. |
| 917 | Does cardiorespiratory fitness moderate the prospective association between physical activity and cardiometabolic risk factors in children? | 2018 | International journal of obesity | This study was excluded because it did not provide the exposure on Tg/glucose or Tg/HDL index values. |
| 918 | Moderate-to-vigorous physical activity, but not sedentary time, predicts changes in cardiometabolic risk factors in 10-y-old children: The Active Smarter Kids Study | 2017 | American Journal of Clinical Nutrition | This study was excluded because it did not provide the exposure on Tg/glucose or Tg/HDL index values. |
| 919 | The prospective association between objectively measured sedentary time, moderate-to-vigorous physical activity and cardiometabolic risk factors in youth: a systematic review and meta-analysis | 2019 | Obesity reviews | This study was excluded because it did not provide the exposure on Tg/glucose or Tg/HDL index values. |
| 920 | Oxidative stress in hypertensive children before and after 1 year of antihypertensive therapy | 2012 | Pediatric nephrology | This study was excluded because it did not provide the exposure on Tg/glucose or Tg/HDL index values. |
| 921 | Change in left ventricular geometry during antihypertensive treatment in children with primary hypertension | 2011 | Pediatric nephrology | This study was excluded because it did not provide the exposure on Tg/glucose or Tg/HDL index values. |
| 922 | The effect of dietary phytosphingosine on cholesterol levels and insulin sensitivity in subjects with the metabolic syndrome | 2010 | European journal of clinical nutrition | This study was excluded because it did not provide the exposure on Tg/glucose or Tg/HDL index values. |
| 923 | Metabolic syndrome, physical activity and cardiac autonomic function | 2012 | Diabetes-metabolism research and reviews | This study was excluded because it did not provide the exposure on Tg/glucose or Tg/HDL index values. |
| 924 | Increased apolipoprotein E level and reduced high-density lipoprotein mean particle size associate with low high-density lipoprotein cholesterol and features of metabolic syndrome | 2010 | Metabolism | This study was excluded because it did not provide the exposure on Tg/glucose or Tg/HDL index values. |
| 925 | Complications of idiopathic thrombocytopenic purpura in pregnancy: a review of literature | 2021 | Jornal Brasileiro de Patologia e Medicina Laboratorial | This study was excluded because it did not provide the exposure on Tg/glucose or Tg/HDL index values. |
| 926 | Relationship between serum concentrations of uric acid, insulin resistance and metabolic alterations in adolescents | 2021 | Jornal Brasileiro de Patologia e Medicina Laboratorial | This study was excluded because it did not provide the exposure on Tg/glucose or Tg/HDL index values. |
| 927 | Validity of a Single-Factor Model Underlying the Metabolic Syndrome in Young Adults: Confirmatory Factor Analysis | 2011 | Revista espanola de cardiologia | This study was excluded because it did not provide the exposure on Tg/glucose or Tg/HDL index values. |
| 928 | Association between Triglyceride Glucose Index and Insulin Resistance among Thai Obese Adolescents | 2020 | Journal of Clinical and Diagnostic Research | This study was excluded because it did not provide the exposure on Tg/glucose or Tg/HDL index values. |
| 929 | Prediction of Insulin Resistance by Modified Triglyceride Glucose Indices in Youth | 2021 | Life-basel | Included |
| 930 | HOMA-IR as a risk factor of gestational diabetes mellitus and a novel simple surrogate index in early pregnancy | 2021 | International Journal of Gynecology and Obstetrics | This study was excluded because it did not provide the exposure on Tg/glucose or Tg/HDL index values. |
| 931 | Lipid levels and selected biomarkers of vascular changes in children with idiopathic headaches - a preliminary report | 2019 | Archives of medical science | This study was excluded because it did not provide the exposure on Tg/glucose or Tg/HDL index values. |
| 932 | Triglycerides/HDL-cholesterol ratio: in adolescents without cardiovascular risk factors | 2012 | Archivos latinoamericanos de nutriciã³n | This study was excluded because it did not provide the exposure on Tg/glucose or Tg/HDL index values. |
| 933 | Relationship between thyroid hormones, resting energy expenditure and cardiometabolic risk factors in euthyroid subjects | 2015 | Clin nutr | This study was excluded because it did not provide the exposure on Tg/glucose or Tg/HDL index values. |
| 934 | Lipoprotein and apolipoprotein differences in black and white girls - The National Heart, Lung, and Blood Institute Growth and Health Study | 1997 | Archives of pediatrics & adolescent medicine | This study was excluded because it did not provide the exposure on Tg/glucose or Tg/HDL index values. |
| 935 | Oral glucose effectiveness and metabolic risk in obese children and adolescents | 2019 | Acta diabetologica | This study was excluded because it did not provide the exposure on Tg/glucose or Tg/HDL index values. |
| 936 | Homocysteine is a marker for metabolic syndrome and atherosclerosis | 2017 | Diabetes and Metabolic Syndrome: Clinical Research and Reviews | This study was excluded because it did not provide the exposure on Tg/glucose or Tg/HDL index values. |
| 937 | Predictability of childhood adiposity and insulin for developing insulin resistance syndrome (syndrome X) in young adulthood: The Bogalusa Heart Study | 2002 | Diabetes | This study was excluded because it did not provide the exposure on Tg/glucose or Tg/HDL index values. |
| 938 | Cardiometabolic risk factors and fat distribution in children and adolescents | 2014 | Journal of Pediatrics | This study was excluded because it did not provide the exposure on Tg/glucose or Tg/HDL index values. |
| 939 | Serum uric acid in HIV infected children and correlation to cardiovascular risk factors | 2019 | HIV and AIDS Review | This study was excluded because it did not provide the exposure on Tg/glucose or Tg/HDL index values. |
| 940 | Body weight stabilization (plateau) as a treatment goal in adolescents with obesity | 2017 | Obesity facts | This study was excluded because it did not provide the exposure on Tg/glucose or Tg/HDL index values. |
| 941 | Association of blood pressure parameters to syndrome X risk factors in adolescents | 2001 | Faseb journal | This study was excluded because it did not provide the exposure on Tg/glucose or Tg/HDL index values. |
| 942 | Low muscle fitness is associated with metabolic risk in youth | 2009 | Medicine and Science in Sports and Exercise | This study was excluded because it did not provide the exposure on Tg/glucose or Tg/HDL index values. |
| 943 | Waist circumference is related to low-grade inflammation in youth | 2010 | International journal of pediatric obesity | This study was excluded because it did not provide the exposure on Tg/glucose or Tg/HDL index values. |
| 944 | Prevalence of Metabolic Syndrome Risk Factors in High School and NCAA Division I Football Players | 2013 | Journal of strength and conditioning research | This study was excluded because it did not provide the exposure on Tg/glucose or Tg/HDL index values. |
| 945 | Plasma lipid concentrations in nondiabetic African American adults: associations with insulin resistance and the metabolic syndrome | 2007 | Metabolism: Clinical and Experimental | This study was excluded because it did not provide the exposure on Tg/glucose or Tg/HDL index values. |
| 946 | Adipogenesis, mtor and ampk pathways link the growth and metabolic actions of growth hormone action in children with GH deficiency (GHD) | 2015 | Endocrine reviews | This study was excluded because it did not provide the exposure on Tg/glucose or Tg/HDL index values. |
| 947 | Antenatal micronutrient supplementation reduces metabolic syndrome in 6- to 8-year-old children in rural Nepal | 2009 | Journal of Nutrition | This study was excluded because it did not provide the exposure on Tg/glucose or Tg/HDL index values. |
| 948 | Prevalence and Risk Factors of Elevated Blood Pressure, Overweight, and Dyslipidemia in Adolescent and Young Adults in Rural Nepal | 2013 | Metabolic syndrome and related disorders | This study was excluded because it did not provide the exposure on Tg/glucose or Tg/HDL index values. |
| 949 | The metabolic syndrome defined | 2007 | Oxidative Stress and Inflammatory Mechanisms in Obesity, Diabetes, and the Metabolic Syndrome | This study was excluded because it did not provide the exposure on Tg/glucose or Tg/HDL index values. |
| 950 | Triglyceride glucose index is superior biomarker for predicting type 2 diabetes mellitus in children and adolescents | 2017 | Endocrine reviews | Included |
| 951 | Cortisol awakening response and fasting morning cortisol make discordant predictions about markers of metabolic syndrome | 2017 | Hormone Research in Paediatrics | This study was excluded because it did not provide the exposure on Tg/glucose or Tg/HDL index values. |
| 952 | Salivary Cortisol Does Not Correlate with Metabolic Syndrome Markers or Subjective Stress in Overweight Children | 2018 | Journal of childhood obesity | This study was excluded because it did not provide the exposure on Tg/glucose or Tg/HDL index values. |
| 953 | Ability of TyG Index as a Marker of Insulin Resistance in Argentinean School Children | 2018 | Journal of Clinical Endocrinology and Metabolism | Included |
| 954 | Association of Fish Consumption and Mercury Exposure During Pregnancy With Metabolic Health and Inflammatory Biomarkers in Children | 2020 | Jama network open | This study was excluded because it did not provide the exposure on Tg/glucose or Tg/HDL index values. |
| 955 | Posttraumatic Stress Disorder Augments Plasma Triglycerides in TT Homozygotes of rs495225 at Growth Hormone Secretagogue Receptor Gene | 2019 | Biochemical genetics | This study was excluded because it did not provide the exposure on Tg/glucose or Tg/HDL index values. |
| 956 | Dysregulation of Adipocytokines Related to Second-Generation Antipsychotics in Normal Fasting Glucose Patients With Schizophrenia | 2012 | Journal of clinical psychopharmacology | This study was excluded because it did not provide the exposure on Tg/glucose or Tg/HDL index values. |
| 957 | A population-based survey of Chronic REnal Disease In Turkey-the CREDIT study | 2011 | Nephrology dialysis transplantation | This study was excluded because it did not provide the exposure on Tg/glucose or Tg/HDL index values. |
| 958 | Uric acid is associated with metabolic syndrome in children and adults in a community: The bogalusa heart study e89696 | 2014 | Plos ONE | This study was excluded because it did not provide the exposure on Tg/glucose or Tg/HDL index values. |
| 959 | Uric acid is associated with metabolic syndrome in children and adults: The bogalusa heart study | 2014 | Circulation | This study was excluded because it did not provide the exposure on Tg/glucose or Tg/HDL index values. |
| 960 | Hyperinsulinemia and insulin resistance related metabolic syndrome | 2001 | Chang Gung medical journal | This study was excluded because it did not provide the exposure on Tg/glucose or Tg/HDL index values. |
| 961 | Fructose and non-fructose sugar intakes in the US population and their associations with indicators of metabolic syndrome | 2011 | Food chem toxicol | This study was excluded because it did not provide the exposure on Tg/glucose or Tg/HDL index values. |
| 962 | Association of GPx1 P198L and CAT C-262T Genetic Variations With Polycystic Ovary Syndrome in Chinese Women | 2019 | Frontiers in Endocrinology | This study was excluded because it did not provide the exposure on Tg/glucose or Tg/HDL index values. |
| 963 | Short-term high dietary fructose intake had no effects on insulin sensitivity and secretion or glucose and lipid metabolismin healthy, obese adolescents | 2008 | Journal of Pediatric Endocrinology and Metabolism | This study was excluded because it did not provide the exposure on Tg/glucose or Tg/HDL index values. |
| 964 | The triglyceride-glucose index as an indicator of insulin resistance and cardiometabolic risk in Brazilian adolescents | 2010 | Journal of Paediatrics and Child Health | Included |
| 965 | Does sleep duration predict metabolic risk in obese adolescents attending tertiary services? A cross-sectional study | 2011 | Sleep | This study was excluded because it did not provide the exposure on Tg/glucose or Tg/HDL index values. |
| 966 | Comparison of the Body Adiposity Index to Body Mass Index in Korean Women | 2014 | Yonsei medical journal | This study was excluded because it did not provide the exposure on Tg/glucose or Tg/HDL index values. |
| 967 | Influence of the polimorphism of KCNJ11, encoding for the pancreatic beta-cell adenosine 5'-triphosphatesensitive potassium channel subunit Kir6.2, on birth weight and auxological parameters, glucose, insulin and lipids secretion in prepubertal SGA children | 2010 | Pediatric diabetes | This study was excluded because it did not provide the exposure on Tg/glucose or Tg/HDL index values. |
| 968 | Metabolic syndrome components among children born small for gestational age: analysis of the first decade of life | 2010 | Pediatric endocrinology, diabetes, and metabolism | This study was excluded because it did not provide the exposure on Tg/glucose or Tg/HDL index values. |
| 969 | Effect of diet and physical exercise treatment on insulin resistance syndrome of schoolchildren | 2008 | Journal of the American College of Nutrition | This study was excluded because it did not provide the exposure on Tg/glucose or Tg/HDL index values. |
| 970 | Evaluation of the risk of diabetes in the offspring of patients with diagnosis of type 2 diabetes | 2009 | Pediatric Endocrinology, Diabetes and Metabolism | This study was excluded because it did not provide the exposure on Tg/glucose or Tg/HDL index values. |
| 971 | Visfatin as a Biomarker of Obesity in Iraqi Adolescences with Metabolic Syndrome | 2021 | Egyptian journal of chemistry | This study was excluded because it did not provide the exposure on Tg/glucose or Tg/HDL index values. |
| 972 | Insulin resistance and obesity-related factors in Prader-Willi syndrome: Comparison with obese subjects | 2005 | Clinical genetics | This study was excluded because it did not provide the exposure on Tg/glucose or Tg/HDL index values. |
| 973 | Nomogram model for the risk of insulin resistance in obese children and adolescents based on anthropomorphology and lipid derived indicators | 2021 | Archives of endocrinology and metabolism | Included |
| 974 | Traditional and emerging cardiometabolic risk profiling among Asian youth with type 2 diabetes: A case-control study | 2020 | Obesity medicine | This study was excluded because it did not provide the exposure on Tg/glucose or Tg/HDL index values. |
| 975 | Mother's pre-pregnancy BMI is an important determinant of adverse cardiometabolic risk in childhood | 2015 | Pediatric diabetes | This study was excluded because it did not provide the exposure on Tg/glucose or Tg/HDL index values. |
| 976 | Serum Bcl-2 concentrations in overweight-obese subjects with nonalcoholic fatty liver disease | 2011 | World journal of gastroenterology | This study was excluded because it did not provide the exposure on Tg/glucose or Tg/HDL index values. |
| 977 | Insulin resistance in children with juvenile systemic lupus erythematosus and investigation of the possibly responsible factors | 2022 | Clinical rheumatology | This study was excluded because it did not provide the exposure on Tg/glucose or Tg/HDL index values. |
| 978 | Insulin resistance in children with juvenile systemic lupus erythematosus and Ä±nvestigation of the possibly responsible factors | 2021 | Clinical rheumatology | This study was excluded because it did not provide the exposure on Tg/glucose or Tg/HDL index values. |
| 979 | Association of Metabolic Syndrome Parameters with TT3 and FT3/FT4 Ratio in Obese Turkish Population | 2012 | Metabolic syndrome and related disorders | This study was excluded because it did not provide the exposure on Tg/glucose or Tg/HDL index values. |
| 980 | TG/HDL-C Ratio for Predicting Insulin Resistance in Obese Children from Beijing, China | 2011 | Int j pediatr obes | Included |
| 981 | Early metabolic improvement following bariatric surgery in morbidly obese adolescents | 2012 | Pediatric Blood and Cancer | This study was excluded because it did not provide the exposure on Tg/glucose or Tg/HDL index values. |
| 982 | Associations of cardiometabolic outcomes with indices of obesity in children aged 5 years and younger | 2019 | Plos One | This study was excluded because it did not provide the exposure on Tg/glucose or Tg/HDL index values. |
| 983 | Cross-Sectional and Longitudinal Associations between Psychosocial Well-Being and Cardiometabolic Markers in European Children and Adolescents | 2020 | Psychosomatic medicine | This study was excluded because it did not provide the exposure on Tg/glucose or Tg/HDL index values. |
| 984 | Triglyceride-glucose index is associated with the risk of myocardial infarction: an 11-year prospective study in the Kailuan cohort | 2021 | Cardiovascular diabetology | This study was excluded because it did not provide the exposure on Tg/glucose or Tg/HDL index values. |
| 985 | Metabolic risk in schoolchildren is associated with low levels of cardiorespiratory fitness, obesity, and parents' nutritional profile | 2016 | J pediatr (rio j) | This study was excluded because it did not provide the exposure on Tg/glucose or Tg/HDL index values. |
| 986 | Associations between salivary cortisol patterns and cardio-metabolic risk factors in Overweight Latino Adolescents (OLAs) | 2013 | Diabetes | This study was excluded because it did not provide the exposure on Tg/glucose or Tg/HDL index values. |
| 987 | The development of risk factors for cardiovascular and metabolic morbidity in a longitudinal study of CAH | 2018 | Endocrine reviews | This study was excluded because it did not provide the exposure on Tg/glucose or Tg/HDL index values. |
| 988 | Type 2 diabetes mellitus and the metabolic syndrome, usefulness of the triglyceride/HDL cholesterol indexes in pediatrics | 2016 | Revista Cubana de Pediatria | This study was excluded because it did not provide the exposure on Tg/glucose or Tg/HDL index values. |
| 989 | Predictive markers of early cardiovascular impairment and insulin resistance in obese pediatric patients | 2021 | Diagnostics | This study was excluded because it did not provide the exposure on Tg/glucose or Tg/HDL index values. |
| 990 | Improved Insulin Sensitivity with a Healthy Low Fat or a Healthy Low Carbohydrate Weight Loss Diet: A Twelve Month Randomized Trial | 2017 | Circulation | This study was excluded because it did not provide the exposure on Tg/glucose or Tg/HDL index values. |
| 991 | Homeostatic model assessment (HOMA) index cut-off values to identify the metabolic syndrome in children | 2005 | Journal of Physiology and Biochemistry | This study was excluded because it did not provide the exposure on Tg/glucose or Tg/HDL index values. |
| 992 | Determinants and pathological role of insulin hypersecretion in nondiabetic adults and adolescents | 2018 | Diabetologia | This study was excluded because it did not provide the exposure on Tg/glucose or Tg/HDL index values. |
| 993 | Salivary markers of hepato-metabolic comorbidities in pediatric obesity | 2019 | Digestive and liver disease | This study was excluded because it did not provide the exposure on Tg/glucose or Tg/HDL index values. |
| 994 | Serum total bilirubin concentrations are inversely associated with total white blood cell counts in an adult population | 2015 | Annals of clinical biochemistry | This study was excluded because it did not provide the exposure on Tg/glucose or Tg/HDL index values. |
| 995 | Triglyceride/HDL-cholesterol ratio as a marker of insulin resistance in overweight and obese children | 2012 | Hormone Research in Paediatrics | This study was excluded because it did not provide the exposure on Tg/glucose or Tg/HDL index values. |
| 996 | Dyslipidemic adolescent and adult african americans have similar cardio-metabolic risk | 2013 | Circulation | This study was excluded because it did not provide the exposure on Tg/glucose or Tg/HDL index values. |
| 997 | Self-Perceived Emotional Distress and Diabetes Risk Among Young Men | 2016 | American journal of preventive medicine | This study was excluded because it did not provide the exposure on Tg/glucose or Tg/HDL index values. |
| 998 | Reduced atherogenic indices in prepubertal girls with precocious adrenarche born appropriate for gestational age in relation to the conundrum of DHEAS | 2013 | Endocrine connections | This study was excluded because it did not provide the exposure on Tg/glucose or Tg/HDL index values. |
| 999 | Prevalence of metabolic syndrome associated with body burden levels of dioxin and related compounds among Japan's general population | Apr | Environ health perspect | This study was excluded because it did not provide the exposure on Tg/glucose or Tg/HDL index values. |
| 1000 | Triglycerides-to-HDLC Ratio as a Marker of Cardiac Disease and Vascular Risk Factors in Adults | 2019 | Jcpsp-journal of the college of physicians and surgeons pakistan | This study was excluded because it did not provide the exposure on Tg/glucose or Tg/HDL index values. |
| 1001 | Plasminogen activator inhibitor-1 and tissue-plasminogen activator in minority adolescents with type 2 diabetes and obesity | 2005 | Pediatric research | This study was excluded because it did not provide the exposure on Tg/glucose or Tg/HDL index values. |
| 1002 | Triglyceride to HDL-C Ratio and Increased Arterial Stiffness in Children, Adolescents, and Young Adults | 2013 | Pediatrics | This study was excluded because it did not provide the exposure on Tg/glucose or Tg/HDL index values. |
| 1003 | Association between waist circumference and waist-to-height ratio with insulin resistance biomarkers in normal-weight adults working in a private educational institution | 2019 | Diabetes & metabolic syndrome-clinical research & reviews | This study was excluded because it did not provide the exposure on Tg/glucose or Tg/HDL index values. |
| 1004 | Association of serum triglyceride-to-high-density lipoprotein cholesterol ratio with insulin resistance and non-alcoholic fatty liver disease in children and adolescents | 2017 | Haseki tip bulteni | This study was excluded because it did not provide the exposure on Tg/glucose or Tg/HDL index values. |
| 1005 | Physical activity and sedentary behaviour in relation to cardiometabolic risk in children: cross-sectional findings from the Physical Activity and Nutrition in Children (PANIC) Study | 2014 | International journal of behavioral nutrition and physical activity | This study was excluded because it did not provide the exposure on Tg/glucose or Tg/HDL index values. |
| 1006 | Longitudinal associations of physical activity and sedentary time with cardiometabolic risk factors in children | 2019 | Scandinavian journal of medicine & science in sports | This study was excluded because it did not provide the exposure on Tg/glucose or Tg/HDL index values. |
| 1007 | Prevalence Of Metabolic Syndrome And Serum Profile Of Adipokines (Leptin And Adiponectin) In Children With Overweight Or Obesity | 2010 | Acta endocrinologica-bucharest | This study was excluded because it did not provide the exposure on Tg/glucose or Tg/HDL index values. |
| 1008 | Socioeconomic inequalities in lipid and glucose metabolism in early childhood in a population-based cohort: the ABCD-Study | 2012 | BMC public health | This study was excluded because it did not provide the exposure on Tg/glucose or Tg/HDL index values. |
| 1009 | Socioeconomic status in relation to lipid and glucose metabolism in early childhood. The ABCD-study | 2012 | Archives of Disease in Childhood | This study was excluded because it did not provide the exposure on Tg/glucose or Tg/HDL index values. |
| 1010 | Exposure to Endocrine Disrupting Chemicals in the Dutch general population is associated with adiposity-related traits | 2020 | Scientific reports | This study was excluded because it did not provide the exposure on Tg/glucose or Tg/HDL index values. |
| 1011 | Fasting serum blood measures of bone and lipid metabolism in children with myelomeningocele for early detection of cardiovascular and bone fragility risk factorsâ€ | 2017 | Journal of Spinal Cord Medicine | This study was excluded because it did not provide the exposure on Tg/glucose or Tg/HDL index values. |
| 1012 | Cardiometabolic risk variables in overweight and obese children: a worldwide comparison | 2011 | Cardiovascular diabetology | This study was excluded because it did not provide the exposure on Tg/glucose or Tg/HDL index values. |
| 1013 | Abdominal Radiotherapy: A Major Determinant of Metabolic Syndrome in Nephroblastoma and Neuroblastoma Survivors | 2012 | Plos one | This study was excluded because it did not provide the exposure on Tg/glucose or Tg/HDL index values. |
| 1014 | Association of Physical Activity and Cardiometabolic Risk in Children 3-12 Years | 2020 | Journal of physical activity & health | This study was excluded because it did not provide the exposure on Tg/glucose or Tg/HDL index values. |
| 1015 | Hyperuricemia in Children and Adolescents with Autism Spectrum Disorder Treated with Risperidone: The Risk Factors for Metabolic Adverse Effects | 2017 | Frontiers in pharmacology | This study was excluded because it did not provide the exposure on Tg/glucose or Tg/HDL index values. |
| 1016 | PPAR alpha and PPAR beta/delta are negatively correlated with proinflammatory markers in leukocytes of an obese pediatric population | 2020 | Journal of inflammation-london | This study was excluded because it did not provide the exposure on Tg/glucose or Tg/HDL index values. |
| 1017 | Genetic determinants of risk factors for cardiovascular disease in a population from rural Brazil | 2007 | Human biology | This study was excluded because it did not provide the exposure on Tg/glucose or Tg/HDL index values. |
| 1018 | Mediterranean-style diet reduces metabolic syndrome components in obese children and adolescents with obesity | 2014 | Bmc pediatrics | This study was excluded because it did not provide the exposure on Tg/glucose or Tg/HDL index values. |
| 1019 | Gender-specific lipid profiles in patients with bipolar disorder | 2011 | Journal of psychiatric research | This study was excluded because it did not provide the exposure on Tg/glucose or Tg/HDL index values. |
| 1020 | Change in metabolic profile after 1-year nutritional-behavioral intervention in obese children | 2015 | Nutrients | This study was excluded because it did not provide the exposure on Tg/glucose or Tg/HDL index values. |
| 1021 | Circulating salicylic acid and metabolic profile in obese children: A case-control study | 2016 | Journal of Pediatric Gastroenterology and Nutrition | This study was excluded because it did not provide the exposure on Tg/glucose or Tg/HDL index values. |
| 1022 | Effectiveness of individual vs. group-based lifestyle intervention on anthropometric and metabolic profile of obese children | 2019 | Journal of Pediatric Gastroenterology and Nutrition | This study was excluded because it did not provide the exposure on Tg/glucose or Tg/HDL index values. |
| 1023 | The importance of diagnostic criteria in the association between the metabolic syndrome and cardiovascular disease in obese subjects | 2005 | International Journal Of Obesity | This study was excluded because it did not provide the exposure on Tg/glucose or Tg/HDL index values. |
| 1024 | Nursing care in childcare services: Acantose nigricans as a marker for metabolic risk | 2013 | Revista latino-americana de Enfermagem | This study was excluded because it did not provide the exposure on Tg/glucose or Tg/HDL index values. |
| 1025 | Clinical and metabolic profile and its relationship with insulin resistance among school children | 2016 | Rev rene | This study was excluded because it did not provide the exposure on Tg/glucose or Tg/HDL index values. |
| 1026 | The TyG index cutoff point and its association with body adiposity and lifestyle in children | 2019 | Jornal de Pediatria | This study was excluded because it did not provide the exposure on Tg/glucose or Tg/HDL index values. |
| 1027 | Answer to the letter "TyG in insulin resistance prediction" | 2020 | Jornal de pediatria | This study was excluded because it did not provide the exposure on Tg/glucose or Tg/HDL index values. |
| 1028 | Factor analysis of the metabolic syndrome components in urban Asian Indian adolescents | 2009 | Asia pacific journal of clinical nutrition | This study was excluded because it did not provide the exposure on Tg/glucose or Tg/HDL index values. |
| 1029 | Inflammation and Cardiometabolic Risk in African American Women Is Reduced by a Pilot Community-Based Educational Intervention | 2016 | J womens health (larchmt) | This study was excluded because it did not provide the exposure on Tg/glucose or Tg/HDL index values. |
| 1030 | Insulin resistence, obesity and metabolic syndrome. Cohort CDC Canarian in Venezuela | 2013 | Salus | This study was excluded because it did not provide the exposure on Tg/glucose or Tg/HDL index values. |
| 1031 | Cardiovascular risk: initial estimation in the study cohort "CDC of the Canary Islands in Venezuela" | 2011 | Investigacion clinica | This study was excluded because it did not provide the exposure on Tg/glucose or Tg/HDL index values. |
| 1032 | Circulating salicylic acid and metabolic profile after 1-year nutritional-behavioral intervention in obese children | 2019 | Journal of Pediatric Gastroenterology and Nutrition | This study was excluded because it did not provide the exposure on Tg/glucose or Tg/HDL index values. |
| 1033 | Circulating salicylic acid and metabolic profile after 1-year nutritionalâ€“behavioral intervention in children with obesity | 2019 | Nutrients | This study was excluded because it did not provide the exposure on Tg/glucose or Tg/HDL index values. |
| 1034 | Circulating Salicylic Acid and Metabolic Profile after 1-Year Nutritional-Behavioral Intervention in Children with Obesity | 2019 | Nutrients | This study was excluded because it did not provide the exposure on Tg/glucose or Tg/HDL index values. |
| 1035 | One year individual or group based lifestyle intervention in obese. Impact on metabolic profile and body composition | 2020 | Obesity reviews | This study was excluded because it did not provide the exposure on Tg/glucose or Tg/HDL index values. |
| 1036 | Visceral Adiposity Index (VAI) is associated with Metabolic Syndrome (MetS) in a cohort of Caucasian obese children and adolescents | 2021 | Journal of Pediatric Gastroenterology and Nutrition | This study was excluded because it did not provide the exposure on Tg/glucose or Tg/HDL index values. |
| 1037 | Visceral adiposity index (VAI) in children and adolescents with obesity: No association with daily energy intake but promising tool to identify metabolic syndrome (MetS) | 2021 | Nutrients | This study was excluded because it did not provide the exposure on Tg/glucose or Tg/HDL index values. |
| 1038 | A Body Shape Index (ABSI) is an independent predictor of cardiovascular risk factors in obese children | 2019 | Obesity facts | This study was excluded because it did not provide the exposure on Tg/glucose or Tg/HDL index values. |
| 1039 | High prevalence of cardiovascular disease risk factors in overweight and obese children and adolescents | 2017 | Acta Paediatrica, International Journal of Paediatrics | This study was excluded because it did not provide the exposure on Tg/glucose or Tg/HDL index values. |
| 1040 | Metabolic profiles of thermal trauma | 1979 | Annals of surgery | This study was excluded because it did not provide the exposure on Tg/glucose or Tg/HDL index values. |
| 1041 | Comparison of the established definition criteria for diagnosing metabolic syndrome between overweight and obese children in Vojvodina | 2011 | Vojnosanitetski pregled | This study was excluded because it did not provide the exposure on Tg/glucose or Tg/HDL index values. |
| 1042 | Biomarkers of cardiometabolic risk in obese/overweight children: effect of lifestyle intervention | 2014 | Physiological research / Academia Scientiarum Bohemoslovaca | This study was excluded because it did not provide the exposure on Tg/glucose or Tg/HDL index values. |
| 1043 | Racial/ethnic discrepancies in the metabolic syndrome begin in childhood and persist after adjustment for environmental factors | 2012 | Nutr metab cardiovasc dis | This study was excluded because it did not provide the exposure on Tg/glucose or Tg/HDL index values. |
| 1044 | The triglyceride/HDL-cholesterol ratio as a tool to predict insulin resistance in obese pediatric patientsâˆ— | 2012 | Journal of Clinical Lipidology | This study was excluded because it did not provide the exposure on Tg/glucose or Tg/HDL index values. |
| 1045 | Interindividual variability and individual responses to exercise training in adolescents with obesity | 2020 | Applied physiology nutrition and metabolism | This study was excluded because it did not provide the exposure on Tg/glucose or Tg/HDL index values. |
| 1046 | Improvement in Glycemic and Lipid Profiles in Type 2 Diabetics with a 90-Day Ketogenic Diet | 2019 | Journal of diabetes research | This study was excluded because it did not provide the exposure on Tg/glucose or Tg/HDL index values. |
| 1047 | The Association between Triglyceride/High-Density Lipoprotein Cholesterol Ratio and All-Cause Mortality in Acute Coronary Syndrome after Coronary Revascularization | 2015 | Plos one | This study was excluded because it did not provide the exposure on Tg/glucose or Tg/HDL index values. |
| 1048 | Beneficial effects of corn silk on metabolic syndrome | 2017 | Current pharmaceutical design | This study was excluded because it did not provide the exposure on Tg/glucose or Tg/HDL index values. |
| 1049 | Yogurt consumption is associated with better diet quality and metabolic profile in American men and women | 2013 | Nutrition research | This study was excluded because it did not provide the exposure on Tg/glucose or Tg/HDL index values. |
| 1050 | Metabolic Syndrome and Its Associated Early-Life Factors among Chinese and Spanish Adolescents: A Pilot Study | 2019 | Nutrients | This study was excluded because it did not provide the exposure on Tg/glucose or Tg/HDL index values. |
| 1051 | Consumption of added sugars and development of metabolic syndrome components among a sample of youth at risk of obesity | 2014 | Applied Physiology, Nutrition and Metabolism | This study was excluded because it did not provide the exposure on Tg/glucose or Tg/HDL index values. |
| 1052 | Adiposity and glucose intolerance exacerbate components of metabolic syndrome in children consuming sugar-sweetened beverages: QUALITY cohort study | 2013 | Pediatric obesity | This study was excluded because it did not provide the exposure on Tg/glucose or Tg/HDL index values. |
| 1053 | Effect of intrauterine growth retardation on insulin sensitivity and plasma adiponectin level in neonates | 2018 | Chinese Journal of Contemporary Pediatrics | This study was excluded because it did not provide the exposure on Tg/glucose or Tg/HDL index values. |
| 1054 | Prevalence of metabolic syndrome in a cohort of Chinese schoolchildren: comparison of two definitions and assessment of adipokines as components by factor analysis | 2013 | Bmc public health | This study was excluded because it did not provide the exposure on Tg/glucose or Tg/HDL index values. |
| 1055 | Evidence for association between paraoxonase 1 gene polymorphisms and polycystic ovarian syndrome in south-west Chinese women | 2012 | European Journal of Endocrinology | This study was excluded because it did not provide the exposure on Tg/glucose or Tg/HDL index values. |
| 1056 | Hemostatic factors in Australian Aboriginal and Torres Strait Islander populations | 2007 | Metabolism-clinical and experimental | This study was excluded because it did not provide the exposure on Tg/glucose or Tg/HDL index values. |
| 1057 | Prevalence of coronary artery disease risk factors and metabolic syndrome in children with high-risk heart disease | 2015 | Congenital heart disease | This study was excluded because it did not provide the exposure on Tg/glucose or Tg/HDL index values. |
| 1058 | Acute activation of metabolic syndrome components in pediatric acute lymphoblastic leukemia patients treated with dexamethasone | 2016 | Plos ONE | This study was excluded because it did not provide the exposure on Tg/glucose or Tg/HDL index values. |
| 1059 | Increased large artery intima media thickness in adolescents with either classical or non-classical congenital adrenal hyperplasia | 2013 | Journal of Endocrinological Investigation | This study was excluded because it did not provide the exposure on Tg/glucose or Tg/HDL index values. |
| 1060 | Parental obesity influences the early onset of obesity and the overweight degree in children | 2017 | Hormone Research in Paediatrics | This study was excluded because it did not provide the exposure on Tg/glucose or Tg/HDL index values. |
| 1061 | Estimation of LDL Particle Size Using Lipid Indices: A Population-Based Study of 1578 Schoolchildren | 2015 | Metabolic Syndrome and Related Disorders | This study was excluded because it did not provide the exposure on Tg/glucose or Tg/HDL index values. |
| 1062 | Identifying Cardiovascular Risk in Survivors of Childhood Leukaemia Treated with Haematopoietic Stem Cell Transplantation and Total Body Irradiation | 2017 | Hormone research in paediatrics | This study was excluded because it did not provide the exposure on Tg/glucose or Tg/HDL index values. |
| 1063 | Aerobic exercise improves insulin sensitivity and lipid metabolism are associated with reduced BMI in obese adolescents | 2011 | Heart | This study was excluded because it did not provide the exposure on Tg/glucose or Tg/HDL index values. |
| 1064 | Obesity and the metabolic syndrome in children and adolescents | 2004 | New England Journal of Medicine | This study was excluded because it did not provide the exposure on Tg/glucose or Tg/HDL index values. |
| 1065 | Differences in the triglyceride to HDL-cholesterol ratio between Palestinian and Israeli adults | 2015 | Plos ONE | This study was excluded because it did not provide the exposure on Tg/glucose or Tg/HDL index values. |
| 1066 | The Triglyceride to High-Density Lipoprotein-Cholesterol Ratio in Adolescence and Subsequent Weight Gain Predict Nuclear Magnetic Resonance-Measured Lipoprotein Subclasses in Adulthood | 2011 | Journal of pediatrics | This study was excluded because it did not provide the exposure on Tg/glucose or Tg/HDL index values. |
| 1067 | Self-initiated changes in physical activity and incidence of Metabolic Syndrome: A longitudinal follow-up study | 2020 | Diabetes research and clinical practice | This study was excluded because it did not provide the exposure on Tg/glucose or Tg/HDL index values. |
| 1068 | Biological Maturation, Central Adiposity, and Metabolic Risk in Adolescents: A Mediation Analysis | 2016 | Childhood obesity | This study was excluded because it did not provide the exposure on Tg/glucose or Tg/HDL index values. |
| 1069 | Women and men have similar amounts of liver and intra-abdominal fat, despite more subcutaneous fat in women: implications for sex differences in markers of cardiovascular risk | 2004 | Diabetologia | This study was excluded because it did not provide the exposure on Tg/glucose or Tg/HDL index values. |
| 1070 | Risk factors for coronary heart disease in obese non-diabetic subjects | 2001 | International Journal of Obesity | This study was excluded because it did not provide the exposure on Tg/glucose or Tg/HDL index values. |
| 1071 | Metabolic and Biochemical Effects of Low-to-Moderate Alcohol Consumption | 2013 | Alcoholism-clinical and experimental research | This study was excluded because it did not provide the exposure on Tg/glucose or Tg/HDL index values. |
| 1072 | Validity of TG/HDL ratio in diagnosing insulin resistance | 2014 | Diabetes Research and Clinical Practice | This study was excluded because it did not provide the exposure on Tg/glucose or Tg/HDL index values. |
| 1073 | Associations of maternal 25-hydroxyvitamin D in pregnancy with offspring cardiovascular risk factors in childhood and adolescence: Findings from the avon longitudinal study of parents and children | 2013 | Heart | This study was excluded because it did not provide the exposure on Tg/glucose or Tg/HDL index values. |
| 1074 | Can clinical factors estimate insulin resistance in type 1 diabetes? | 2000 | Diabetes | This study was excluded because it did not provide the exposure on Tg/glucose or Tg/HDL index values. |
| 1075 | Intraperitoneal fat and insulin resistance in obese adolescents | 2010 | Obesity | This study was excluded because it did not provide the exposure on Tg/glucose or Tg/HDL index values. |
| 1076 | Intraperitoneal Fat and Insulin Resistance in Obese Adolescents Glaser Pediatric Research Network Obesity Study Group | 2010 | Obesity | This study was excluded because it did not provide the exposure on Tg/glucose or Tg/HDL index values. |
| 1077 | Adiponectin in childhood and adolescent obesity and its association with inflammatory markers and components of the metabolic syndrome | 2006 | Journal of Clinical Endocrinology and Metabolism | This study was excluded because it did not provide the exposure on Tg/glucose or Tg/HDL index values. |
| 1078 | Lipid and carbohydrate parameters in children with chronic hepatitis C | 2011 | Advances in Medical Sciences | This study was excluded because it did not provide the exposure on Tg/glucose or Tg/HDL index values. |
| 1079 | Lipid metabolism in children with chronic hepatitis C, a preliminary report | 2006 | Hepato-gastroenterology | This study was excluded because it did not provide the exposure on Tg/glucose or Tg/HDL index values. |
| 1080 | Dietary intake and lipid profile in children and adolescents with cystic fibrosis | 2017 | Journal of cystic fibrosis | This study was excluded because it did not provide the exposure on Tg/glucose or Tg/HDL index values. |
| 1081 | Nonalcoholic fatty liver disease severity is associated with the ratios of total cholesterol and triglycerides to high-density lipoprotein cholesterol | 2016 | Journal of clinical lipidology | This study was excluded because it did not provide the exposure on Tg/glucose or Tg/HDL index values. |
| 1082 | Association between triglyceride-glucose index and risk of arterial stiffness: a cohort study | 2021 | Cardiovascular diabetology | This study was excluded because it did not provide the exposure on Tg/glucose or Tg/HDL index values. |
| 1083 | Effects of different diet-induced postnatal catch-up growth on glycolipid metabolism in intrauterine growth retardation male rats | 2020 | Experimental and therapeutic medicine | This study was excluded because it did not provide the exposure on Tg/glucose or Tg/HDL index values. |
| 1084 | Intima-media thickness in obese children before and after weight loss | 2006 | Pediatrics | This study was excluded because it did not provide the exposure on Tg/glucose or Tg/HDL index values. |
| 1085 | The common rs9939609 variant of the fat mass and obesity-associated gene is associated with obesity risk in children and adolescents of Beijing, China | 2010 | Bmc medical genetics | This study was excluded because it did not provide the exposure on Tg/glucose or Tg/HDL index values. |
| 1086 | Relationship between serum lipoprotein ratios and insulin resistance in polycystic ovary syndrome | 2012 | International Journal of Endocrinology | This study was excluded because it did not provide the exposure on Tg/glucose or Tg/HDL index values. |
| 1087 | Relationship of "weekend warrior" and regular physical activity patterns with metabolic syndrome and its associated diseases among Chinese rural adults | 2018 | J sports sci | This study was excluded because it did not provide the exposure on Tg/glucose or Tg/HDL index values. |
| 1088 | Associations of metabolic disorder factors with the risk of uncontrolled hypertension: a follow-up cohort in rural China | 2017 | Sci rep | This study was excluded because it did not provide the exposure on Tg/glucose or Tg/HDL index values. |
| 1089 | Physical Activity and Sedentary Behavior Associated with Components of Metabolic Syndrome among People in Rural China | 2016 | Plos One | This study was excluded because it did not provide the exposure on Tg/glucose or Tg/HDL index values. |
| 1090 | Temporal relationship between body mass index and triglyceride-glucose index and its impact on the incident of hypertension | 2019 | Nutrition metabolism and cardiovascular diseases | This study was excluded because it did not provide the exposure on Tg/glucose or Tg/HDL index values. |
| 1091 | Clinical effect of subcutaneous insulin injection combined with metformin for type 2 diabetes mellitus in children | 2014 | World Chinese Journal of Digestology | This study was excluded because it did not provide the exposure on Tg/glucose or Tg/HDL index values. |
| 1092 | Serum adiponectin, body adiposity and metabolic parameters in obese Egyptian children with down syndrome | 2021 | Journal of Pediatric Endocrinology and Metabolism | This study was excluded because it did not provide the exposure on Tg/glucose or Tg/HDL index values. |
| 1093 | Serum retinol binding protein 4 is negatively related to beta cell function in Chinese women with non-alcoholic fatty liver disease: a cross-sectional study | 2013 | Lipids in health and disease | This study was excluded because it did not provide the exposure on Tg/glucose or Tg/HDL index values. |
| 1094 | Association of triglyceride to HDL cholesterol ratio with cardiometabolic outcomes | 2019 | Journal of investigative medicine | This study was excluded because it did not provide the exposure on Tg/glucose or Tg/HDL index values. |
| 1095 | Maternal gestational diabetes and childhood hyperlipidemia | 2021 | Diabetic medicine | This study was excluded because it did not provide the exposure on Tg/glucose or Tg/HDL index values. |
| 1096 | Pentraxin 3 and the TyG Index as Two Novel Markers to Diagnose NAFLD in Children | 2021 | Disease markers | This study was excluded because it did not provide the exposure on Tg/glucose or Tg/HDL index values. |
| 1097 | Elevated triglyceride-to-HDL cholesterol ratio is an indicator for insulin resistance in middle-aged and elderly Taiwanese population: a cross-sectional study | 2019 | Lipids in health and disease | This study was excluded because it did not provide the exposure on Tg/glucose or Tg/HDL index values. |
| 1098 | Health and saliva microbiomes of a semi-urbanized indigenous tribe in Peninsular Malaysia | 2019 | F1000Research | This study was excluded because it did not provide the exposure on Tg/glucose or Tg/HDL index values. |
| 1099 | Triglyceride to high density lipoprotein cholesterol ratio among adolescents is associated with adult hypertension: the Kangwha study | 2018 | Lipids in health and disease | This study was excluded because it did not provide the exposure on Tg/glucose or Tg/HDL index values. |
| 1100 | Diagnostic accuracy of the tri-ponderal mass index in identifying the unhealthy metabolic obese phenotype in obese patients | 2021 | Anales de Pediatria | This study was excluded because it did not provide the exposure on Tg/glucose or Tg/HDL index values. |
| 1101 | Salivary latent trait cortisol (LTC): Relation to lipids, blood pressure, and body composition in middle childhood | 2016 | Psychoneuroendocrinology | This study was excluded because it did not provide the exposure on Tg/glucose or Tg/HDL index values. |
| 1102 | Several genetic polymorphisms interact with overweight/obesity to influence serum lipid levels | 2012 | Cardiovascular diabetology | This study was excluded because it did not provide the exposure on Tg/glucose or Tg/HDL index values. |
| 1103 | High plasma concentration of remnant lipoprotein cholesterol in obese children and adolescents | 2006 | Diabetes care | This study was excluded because it did not provide the exposure on Tg/glucose or Tg/HDL index values. |
| 1104 | The Triglyceride-To-High density lipoprotein cholesterol ratio in overweight Korean children and adolescents | 2017 | Annals of Pediatric Endocrinology and Metabolism | Included |
| 1105 | Relationship between insulin resistance and serum alanine aminotransferase as a surrogate of NAFLD (nonalcoholic fatty liver disease) in obese Korean children | 2008 | Diabetes Research and Clinical Practice | This study was excluded because it did not provide the exposure on Tg/glucose or Tg/HDL index values. |
| 1106 | Antioxidant micronutrients and their performance in Hispanics infected with the human immunodeficiency virus and/or hepatitis C virus | 2009 | Faseb journal | This study was excluded because it did not provide the exposure on Tg/glucose or Tg/HDL index values. |
| 1107 | Comparison of triglyceride and glucose index and homeostatic model assessment for insulin resistance in children and adolescents with type 2 diabetes mellitus | 2021 | Hormone research in paediatrics | This study was excluded because it did not provide the exposure on Tg/glucose or Tg/HDL index values. |
| 1108 | The Correlations between Cardiorespiratory Fitness Levels and Body Mass Index, Metabolic Syndrome Risk Factors, Homeostatic Model Assessment-Insulin Resistance, and High Sensitivity C-Reactive Protein in Male High School Students | 2011 | Korean Journal of Health Promotion | This study was excluded because it did not provide the exposure on Tg/glucose or Tg/HDL index values. |
| 1109 | Obesity, central fat patterning, and their metabolic correlates among the inuit of the central Canadian Arctic | 1996 | Human biology | This study was excluded because it did not provide the exposure on Tg/glucose or Tg/HDL index values. |
| 1110 | Three clustering patterns among metabolic syndrome risk factors and their associations with dietary factors in Korean adolescents: based on the Korea National Health and Nutrition Examination Survey of 2007-2010 | 2015 | Nutrition research and practice | This study was excluded because it did not provide the exposure on Tg/glucose or Tg/HDL index values. |
| 1111 | Novel associations of serum adropin and lipopolysaccharide-binding protein versus lipid profiles in childhood obesity | 2020 | Journal of pediatric endocrinology & metabolism | This study was excluded because it did not provide the exposure on Tg/glucose or Tg/HDL index values. |
| 1112 | Association between adipocyte fatty acid-binding protein levels and childhood obesity in Korean children | 2009 | Metabolism: Clinical and Experimental | This study was excluded because it did not provide the exposure on Tg/glucose or Tg/HDL index values. |
| 1113 | Serum magnesium status among obese children and adolescents | 2016 | Egyptian pediatric association gazette | This study was excluded because it did not provide the exposure on Tg/glucose or Tg/HDL index values. |
| 1114 | Are metabolic syndrome and its components in obese children influenced by the overweight status or the insulin resistance? | 2011 | Endokrynologia polska | This study was excluded because it did not provide the exposure on Tg/glucose or Tg/HDL index values. |
| 1115 | Correlation between anthropometric measures and cardiometabolic risk factors in 13-year-old urban Malaysian | 2014 | Obesity reviews | This study was excluded because it did not provide the exposure on Tg/glucose or Tg/HDL index values. |
| 1116 | Growth arrest-specific 6 (Gas 6) protein is associated with adiposity and metabolic syndrome in obese children and adolescents | 2019 | Hormone Research in Paediatrics | This study was excluded because it did not provide the exposure on Tg/glucose or Tg/HDL index values. |
| 1117 | Association of adiponectin gene polymorphisms 276G>T with obesity and biochemical parameters in adolescents | 2014 | International Journal of Pharmacy and Pharmaceutical Sciences | This study was excluded because it did not provide the exposure on Tg/glucose or Tg/HDL index values. |
| 1118 | Insulin resistance in pulmonary arterial hypertension | 2009 | European respiratory journal | This study was excluded because it did not provide the exposure on Tg/glucose or Tg/HDL index values. |
| 1119 | Microalbuminuria and elevated blood pressure in obese children | 2010 | Pediatric nephrology | This study was excluded because it did not provide the exposure on Tg/glucose or Tg/HDL index values. |
| 1120 | Influence of physical fitness on cardio-metabolic risk factors in European children. The IDEFICS study | 2016 | International Journal of Obesity | This study was excluded because it did not provide the exposure on Tg/glucose or Tg/HDL index values. |
| 1121 | Sagittal Abdominal Diameter does not Predict Metabolic Traits Better than Waist Circumference-Related Measures of Abdominal Obesity in Obese Subjects | 2018 | Experimental and clinical endocrinology & diabetes | This study was excluded because it did not provide the exposure on Tg/glucose or Tg/HDL index values. |
| 1122 | Diagnostic Criteria for Metabolic Syndrome: Caucasians Versus African-Americans | 2010 | Metabolic syndrome and related disorders | This study was excluded because it did not provide the exposure on Tg/glucose or Tg/HDL index values. |
| 1123 | Relationship between long-term use of a typical antipsychotic medication by Chinese schizophrenia patients and the bone turnover markers serum osteocalcin and beta-CrossLaps | 2016 | Schizophrenia research | This study was excluded because it did not provide the exposure on Tg/glucose or Tg/HDL index values. |
| 1124 | Early and late postnatal accelerated growth have distinct effects on metabolic health in normal birth weight infants | 2017 | Frontiers in Endocrinology | This study was excluded because it did not provide the exposure on Tg/glucose or Tg/HDL index values. |
| 1125 | Familial aggregation of metabolic syndrome in adolescents with paternal metabolic syndrome | 2010 | Zhonghua Liu Xing Bing Xue Za Zhi | This study was excluded because it did not provide the exposure on Tg/glucose or Tg/HDL index values. |
| 1126 | Apolipoprotein A-I and B levels, dyslipidemia and metabolic syndrome in south-west Chinese women with PCOS | 2012 | Human reproduction | This study was excluded because it did not provide the exposure on Tg/glucose or Tg/HDL index values. |
| 1127 | Prevalence of metabolically obese but normal weight (MONW) and metabolically healthy but obese (MHO) in Chinese Beijing urban subjects | 2017 | Biosci trends | This study was excluded because it did not provide the exposure on Tg/glucose or Tg/HDL index values. |
| 1128 | Association between triglyceride glucose index and peak growth hormone in children with short stature | 2021 | Scientific reports | This study was excluded because it did not provide the exposure on Tg/glucose or Tg/HDL index values. |
| 1129 | Effects of R-HGH replacement therapy on glucose and lipid metabolism and thyroid function in children with idiopathic short stature | 2014 | Chinese Journal of Contemporary Pediatrics | This study was excluded because it did not provide the exposure on Tg/glucose or Tg/HDL index values. |
| 1130 | Relationship between blood lipid profiles and pancreatic islet Î² cell function in Chinese men and women with normal glucose tolerance: a cross-sectional study | 2012 | BMC public health | This study was excluded because it did not provide the exposure on Tg/glucose or Tg/HDL index values. |
| 1131 | Effects of metabolic syndrome on intestinal flora, inflammatory factors, and infants of pregnant patients | 2020 | Clinical laboratory | This study was excluded because it did not provide the exposure on Tg/glucose or Tg/HDL index values. |
| 1132 | Clustering and determinants of cardiometabolic risk factors among Filipino young adults | 2014 | Asia Pacific Journal of Clinical Nutrition | This study was excluded because it did not provide the exposure on Tg/glucose or Tg/HDL index values. |
| 1133 | Androgen replacement therapy in turner syndrome: A pilot study | 2009 | Journal of Clinical Endocrinology and Metabolism | This study was excluded because it did not provide the exposure on Tg/glucose or Tg/HDL index values. |
| 1134 | Serum Insulin, Insulin-like Growth Factor-I and Insulin-like Growth Factor Binding Protein-3 Levels in Obese Adolescents | 2005 | Clinical and Experimental Pediatrics | This study was excluded because it did not provide the exposure on Tg/glucose or Tg/HDL index values. |
| 1135 | The Prevalence of Metabolic Syndrome in Children and Adolescents Born Small for Gestational Age | 2007 | Annals of Pediatirc Endocrinology & Metabolism | This study was excluded because it did not provide the exposure on Tg/glucose or Tg/HDL index values. |
