# Supplementary material for: Diagnostic accuracy of triglyceride to glucose index and triglyceride/high-density lipoprotein index for insulin resistance among children and adolescents: A systematic review
Source: PLoS One. 2025 Jun 25;20(6):e0326179. doi: 10.1371/journal.pone.0326179 (PMC12192287; doi:10.1371/journal.pone.0326179)
Supplement: S5 Table — Full list of excluded studies with justification. (DOCX) [file pone.0326179.s005.docx]

**S5 Table. Studies excluded.**

| **FIRST AUTHOR** | **TITLE** | **YEAR** | **REASON FOR EXCLUSION** |
| --- | --- | --- | --- |
| Alemzadeh | Comparison of Apolipoprotein (ApoB/ApoA-1) and Lipoprotein (Total Cholesterol/HDL) Ratios in Obese Adolescents | 2018 | Other outcome |
| Ansari | Study of Insulin Resistance in Patients with B Thalassemia Major and Validity of Triglyceride Glucose (TYG) Index | 2018 | This study was excluded because it did not have a population composed only of children or adolescents. |
| Aslan | Triglycerides/high-density lipoprotein cholesterol is a predictor similar to the triglyceride-glucose index for the diagnosis of metabolic syndrome using International Diabetes Federation criteria of insulin resistance in obese adolescents: A cross-sectional study | 2020 | Other outcome |
| Cabello | Comparison of HOMA-IR ratio and TG/HDL-C ratio for the diagnosis of metabolic syndrome in obese 3-5 years old children | 2016 | This study was excluded because it was a conference abstract. |
| Calcaterra | Sex-Specific Differences in the Relationship between Insulin Resistance and Adiposity Indexes in Children and Adolescentes with Obesity | 2021 | No evaluate diagnostic accuracy |
| Chiarpenello | Evaluation index triglycerides and glucose as a marker of insulin resistnace and its comparison with other markers of insulin resistance in obese children | 2016 | This study was excluded because it was a conference abstract. |
| Chiarpenello, | Trglycerides/HDL index in a pediatric population from the city of Rosario and its surrounding area | 2018 | Other outcome |
| Chu | Risk assessment of metabolic syndrome in adolescents using the triglyceride/high-density lipoprotein cholesterol ratio and the total choelsterol/high-density lipoprotein cholesterol ratio | 2019 | Other outcome |
| Cuartero | An easy way to identify obese children and adolescents with metabolic risk | 2017 | This study was excluded because it was a conference abstract. |
| De Giorgis | Triglycerides-to-HDL ratio as a new marker of endothelial dysfunction in obese prepubertal children | 2014 | This study was excluded because it did not assess diagnostic accuracy. |
| Elrayess | Prevalence and Predictor of Insulin Resistance in Non-Obese Healthy Young Females in Qatar | 2020 | This study was excluded because it did not assess diagnostic accuracy. |
| Eng | TG/HDL ratio as a predictor in insulin resistance in U.S.A adolescents | 2012 | This study was excluded because it was a conference abstract. |
| Gesteiro | The triglyceride-glucose index, an insulin resistance marker in newborns? | 2018 | Other outcome |
| Gidding | Adolescent and adult African Americans have similar metabolic dyslipidemia | 2015 | Other outcome |
| Hirschler | Association between triglyceride-to-HDL-C ratio and insulin resistance among argentine indian and mixed population school children | 2014 | Other outcome |
| Hirchsler | Triglyceride to HDL-C ratio levels in indigenous Argentinean childrean living at different altitudes | 2017 | This study was excluded because it was a conference abstract. |
| Iwani | Triglyceride to HDL-C Ratio is Associated with Insulin Resistance in Overweight and Obese Children | 2017 | Other outcome |
| Kabakoglu | Triglyceride-to-high density lipoprotein cholesterol ratio and triglyceride-glucose index in the perinatal period of neonates | 2021 | Other outcome |
| Kang | The triglyceride-to-high density lipoprotein cholesterol ratio in overweight Korean children | 2015 | This study was excluded because it did not assess diagnostic accuracy. |
| Kawadry | Triglyceride: HDL-Cholesterol Ratio as a Surrogate Marker for Insulin Resistance and Inflammation in Pediatrick Obesity | 2010 | Other outcome |
| Kim | Triglycerides/HDL cholesterol ratio and total cholesterol/HDL cholesterol ratio: surrogate marker for metabolic syndrome in adolescents | 2017 | This study was excluded because it was a conference abstract. |
| Krawchyzk | Usefulness of the Triglycerides yo High-Density Lipoprotein Cholesterol ratio (TG/HDL-C) in prediction of metabolic syndrome in Polish obese children and adolescents | 2018 | Other outcome |
| Lopes | TyG in insulin resistance prediction | 2020 | Letter to the editor |
| Massa | The triglyceride-to-HDL cholesterol ratio is associated with insulin resistance in obese boys but not in obese girls | 2015 | This study was excluded because it was a conference abstract. |
| Mayman | Association of triglyceride/HDL-cholesterol ratio with insulin resistance indices in obese children | 2005 | This study was excluded because it was a conference abstract. |
| Mosca | Triglycerides/HDL-cholesterol ratio in a population of adolescent overweight/obese (OW/OB): A new diagnosis marker of insulin resistance (IR) and metabolic syndrome (MS)? | 2014 | This study was excluded because it was a conference abstract. |
| Murguía | Plasma triglyceride/HDL-cholesterol ratio, insulin resistance, and cardiometabolic risk in young adults | 2013 | This study was excluded because it had a young adult population that did not consist only of children or adolescents. |
| Olson | The triglyceride to HDL ratio and its relationship to insulin resistance in pre- and postpubertal children: Observation from the wausau SCHOOL project | 2012 | Other outcome |
| Park | Association between the triglyceride to high-density lipoprotein cholesterol ratio and insulin resistance in Korean adolescentes: A nationwide population-base study | 2016 | Other outcome |
| Simental | The product of fasting glucose and triglycerides as surrogate for identifying insulin resistance in apparently healthy subjects | 2008 | This study was excluded because it did not have a population composed only of children or adolescents. |
| Somdee | Association between Triglyceride Glucose Index and Insulin Resistance among Thai Obese Adolescents | 2020 | Other outcome |
| Soutelo | Triglycerides/HDL-cholesterol ratio: in adolescents without cardiovascular risk factors | 2012 | Other outcome |
| Tsiroukidou | Triglyceride/HDL-cholesterol ratio as a marker of insulin resistance in overweight and obese children | 2012 | This study was excluded because it was a conference abstract. |
| Uystol | Association of serum triglyceride-to-high-density lipoprotein cholesterol ratio with insulin resistance and non-alcoholic fatty liver disease in children and adolescents | 2017 | This study was excluded because it did not assess diagnostic accuracy. |
| Walkup | The triglyceride/HDL-cholesterol ratio as a tool to predict insulin resistance in obese pediatric patients | 2012 | This study was excluded because it was a conference abstract. |
| Wickramasinghe | Validity of TG/HDL ratio in diagnosing insulin resistance | 2014 | This study was excluded because it was a conference abstract. |
| Yoon | Comparison of triglyceride and glucose index and homeostatic model assessment for insulin resistance in children and adolescents with type 2 diabetes mellitus | 2021 | Other outcome |
