# Supplementary material for: Diagnostic accuracy of triglyceride to glucose index and triglyceride/high-density lipoprotein index for insulin resistance among children and adolescents: A systematic review
Source: PLoS One. 2025 Jun 25;20(6):e0326179. doi: 10.1371/journal.pone.0326179 (PMC12192287; doi:10.1371/journal.pone.0326179)
Supplement: S6 Table — Detailed results of QUADAS-2 assessments for each study included. (DOCX) [file pone.0326179.s006.docx]

**S6 Table. Risk of bias (QUADAS-2) according to each domain**

| **STUDY** | **PATIENT SELECTION** | **INDEX TEST** | **REFERENCE STANDARD** | **FLOW AND TIMING** |
| --- | --- | --- | --- | --- |
| Hannon, 2006 [31] | Low | Low | Low | Low |
| Giannini, 2011 [30] | Low | Low | Low | Low |
| Bridges, 2016 [25] | Low | Low | High^a^ | Low |
| Kang, 2017 [9] | Low | Low | High^a^ | Low |
| Yoo, 2017 [32] | Low | Low | High^a^ | Low |
| Rodriguez, 2017 [35] | Low | Unclear^b^ | High^a^ | Low |
| Alvim, 2018 [23] | Low | Low | High^a^ | Low |
| Behiry, 2019 [24] | Low | Unclear^b^ | High^a^ | Low |
| Calcaterra, 2019 [27] | Low | Unclear^b^ | High^a^ | Low |
| Locateli, 2019 [33] | High^c^ | Low | High^a^ | Low |
| Brito, 2020 [26] | Low | Low | High^a^ | Low |
| Dikaiakou, 2020 [13] | Low | Low | High^a^ | Low |
| García, 2020 [29] | Low | Unclear | High^a^ | Low |
| Rodriguez, 2020 [34] | Low | Low | High^a^ | Low |
| Sanchez, 2020 [36] | Low | Low | High^a^ | Low |
| Song, 2021 [37] | Low | Low | High^a^ | Low |
| Hirschler, 2022 [38] | Low | Low | High^a^ | Low |
| Yoong, 2022 [41] | Low | Low | High^a^ | Low |
| You-Xiang, 2023 [39] | Low | Low | High^a^ | Low |
| Reckziegel, 2023 [40] | Low | Low | High^a^ | Low |
| Zhang, 2024 [28] | Low | Low | High^a^ | Low |
| Explications:  The HOMA-IR test was used instead of the euglycemic-hyperinsulinemic clamp test as the reference test  It was not described whether the results of the index test were interpreted without knowledge of the results of the reference test  A consecutive sample of patients was not enrolled. | | | | |
