# Supplementary material for: Diagnostic accuracy of triglyceride to glucose index and triglyceride/high-density lipoprotein index for insulin resistance among children and adolescents: A systematic review
Source: PLoS One. 2025 Jun 25;20(6):e0326179. doi: 10.1371/journal.pone.0326179 (PMC12192287; doi:10.1371/journal.pone.0326179)
Supplement: Attachment — Submitted filename: Comments to editor.docx [file pone.0326179.s008.docx]

Comments to the Editor

This manuscript is a meaningful attempt to elucidate the diagnostic value of triglyceride to glucose index(TyG) and triglyceride/high-density lipoprotein index(Tg/HDL) as a surrogate marker of HOMA-IR or insulin resistance itself.

As a clinician, evaluating insulin resistance or calculating HOMA-IR is tricky task because they require some conditions to calculate. Considering that, try to find new simple marker is really meaningful attempt. I think.

However, the attempt was not successful because of high heterogeneity of included studies. Actually, the authors could not even perform meta-analysis.

However, these inconclusive and somehow limited results still indicate TyG and Tg/HDL to be a promising simple screening marker of metabolic syndrome.

As I commented author too, although the results were negative, it is worth to be published, I think.

Thank you for giving me an opportunity to review such an interesting manuscript.
